# Isolation and characterization of a novel *Sphingobium yanoikuyae* strain variant that uses biohazardous saturated hydrocarbons and aromatic compounds as sole carbon sources

**DOI:** 10.12688/f1000research.25284.1

**Published:** 2020-07-24

**Authors:** Mautusi Mitra, Kevin Manoap-Anh-Khoa Nguyen, Taylor Wayland Box, Jesse Scott Gilpin, Seth Ryan Hamby, Taylor Lynne Berry, Erin Harper Duckett

**Affiliations:** 1 Biology Department, University of West Georgia, Carrollton, GA, 30118, USA; 2 Department of Mechanical Engineering, Kennesaw State University, Marietta, GA, 30060, USA; 3 Carrollton High School, Carrollton, GA, 30117, USA; 4 Department of Chemistry and Biochemistry, University of North Georgia, Dahlonega, GA, 30597, USA

**Keywords:** Chlamydomonas, Sphingobium yanoikuyae, TAP medium, CC4533, bioremediation, 16S rRNA gene, neomycin-sensitivity, beta-carotene

## Abstract

**Background:** Green micro-alga, *Chlamydomonas reinhardtii* (a Chlorophyte), can be cultured in the laboratory heterotrophically or photo-heterotrophically in **T**ris- **P**hosphate- **A**cetate (TAP) medium, which contains acetate as the carbon source. *Chlamydomonas* can convert acetate in the TAP medium to glucose via the glyoxylate cycle, a pathway present in many microbes and higher plants. A novel bacterial strain, CC4533, was isolated from a contaminated TAP agar medium culture plate of a *Chlamydomonas *wild type strain. In this article, we present our research on the isolation, and biochemical and molecular characterizations of CC4533.

**Methods:** We conducted several microbiological tests and spectrophotometric analyses to biochemically characterize CC4533. The 16S rRNA gene of CC4533 was partially sequenced for taxonomic identification. We monitored the growth of CC4533 on Tris-Phosphate (TP) agar medium (lacks a carbon source) containing different sugars, aromatic compounds and saturated hydrocarbons, to see if CC4533 can use these chemicals as the sole source of carbon.

**Results:** CC4533 is a Gram-negative, non-enteric yellow pigmented, aerobic, mesophilic bacillus. It is alpha-hemolytic and oxidase-positive. CC4533 can ferment glucose, sucrose and lactose, is starch hydrolysis-negative, resistant to penicillin, polymyxin B and chloramphenicol. CC4533 is sensitive to neomycin. Preliminary spectrophotometric analyses indicate that CC4533 produces b-carotenes. NCBI-BLAST analyses of the partial 16S rRNA gene sequence of CC4533 show 99.55% DNA sequence identity to that of *Sphingobium yanoikuyae *strain PR86 and *S. yanoikuyae *strain NRB095. CC4533 can use cyclo-chloroalkanes, saturated hydrocarbons present in car motor oil, polyhydroxyalkanoate, and mono- and poly-cyclic aromatic compounds, as sole carbon sources for growth.

**Conclusions:** Taxonomically, CC4533 is very closely related to the alpha-proteobacterium *S. yanoikuyae*, whose genome has been sequenced. Future research is needed to probe the potential of CC4533 for environmental bioremediation. Whole genome sequencing of CC4533 will confirm if it is a novel strain of *S. yanoikuyae *or a new *Sphingobium* species.

## Introduction

The bacterium *Sphingomonas* belongs to the class Alphaproteobacteria, order Sphingomonadales and class Sphingomonadaceae. The genus *Sphingomonas* was first described by Yabuuchi *et al.* (1990) as a strictly aerobic, chemoheterotrophic, yellow-pigmented, Gram-negative, rod-shaped bacterium containing glycosphingolipids in cell-envelope ^1^. Takeuchi *et al.* (1994) described four phylogenetic clusters in the genus based on 16S rRNA gene sequence data, and later, combined phylogenetic, chemotaxonomic and physiological analyses to split the genus into four genera: *Sphingomonas*, *Sphingobium*, *Novosphingobium* and *Sphingopyxis* ^2, 3^. Recently, the genus *Sphingosinicella* has been added to the family Sphingomonadaceae ^4^. The type species of the ever-expanding genus *Sphingobium* is *Sphingobium yanoikuyae* and species with valid published names are *S. amiense*, *S. aromaticiconvertens*, *S. chlorophenolicum*, *S. chungbukense*, *S. cloacae*, *S. francense*, *S. fuliginis, S. herbicidovorans*, *S. indicum*, *S. japonicum*, *S. olei*, *S. xenophagum*, *S. yanoikuyae*, *S. ummariense* and *S. rhizovicinum* ^5– 12^.

In our research laboratory, we employ the green micro-alga *Chlamydomonas reinhardtii,* a member of the family Chlorophyceae, as a model system to study oxygenic photosynthesis. *C. reinhardtii* is grown at the laboratory in the presence (photo-heterotrophically) or absence of light (heterotrophically) in the acetate-containing **T**ris- **P**hosphate- **A**cetate (TAP) medium ^13^. TAP contains 0.1% acetate as the sole carbon source ^13^. The acetate in TAP medium is used by *Chlamydomonas* for net biosynthesis of glucose via the glyoxylate/C2 cycle ^14^. This allows *Chlamydomonas* to make sugar heterotrophically in the dark or photo-heterotrophically in the light, without being completely dependent on photosynthesis for glucose biosynthesis ^14^. Many bacteria possess glyoxylate cycle and can utilize acetate as a carbon source, like *Chlamydomonas* ^15, 16^. Hence, we often encounter bacterial contamination on *Chlamydomonas* TAP-agar medium culture plates. We observed a yellow-pigmented bacterial contamination on the TAP-agar medium culture plate of the *Chlamydomonas* wild type strain, CC4533. This bacterium was able to grow on the TAP-agar medium because it was able to use acetate as the carbon source. We named this bacterium as CC4533 because it contaminated the *Chlamydomonas* wild type strain CC4533. A team of four undergraduate research students from the University of West Georgia and, one high school student from the Carrollton High School in Georgia (USA) were assigned a research project to test four antibiotics that were available in our laboratory to determine which one of these four antibiotics would be suitable for use to minimize *Chlamydomonas* contamination by CC4533. After the antibiotic-sensitivity of CC4533 was determined, we performed additional microbiological and molecular experiments to better characterize the bacterial strain, CC4533. 

We employed a modified version of the Kirby-Bauer (KB) disc diffusion antibiotic susceptibility test to determine antibiotic sensitivity of CC4533. Two different doses of four antibiotics (penicillin, neomycin, chloramphenicol and polymyxin B) were tested in the KB experiment. The results from the KB test show that CC4533 is resistant to penicillin and chloramphenicol but is sensitive to neomycin. Although CC4533 is more sensitive to polymyxin B at higher dose, polymyxin B cannot be used for controlling *Chlamydomonas* contamination, as at higher dose Chlamydomonas growth is affected. We found that 50 µg/mL of neomycin in TAP medium was potent for eradicating CC4533 contamination on *Chlamydomonas* media plates in our research laboratory, without hindering *Chlamydomonas* growth.

We performed standard microbiological tests to biochemically characterize CC4533. These tests show that CC4533 is an aerobic, non-enteric, Gram-negative long rod shaped bacterium (bacillus). CC4533 does not grow on Mannitol Salt Agar and MacConkey agar. CC4533 is starch hydrolysis-negative, alpha-hemolytic and, is cytochrome c oxidase-positive. CC4533 has a bright yellow pigmentation on **L**ysogeny **B**roth (LB) agar and a pale yellow pigmentation on TAP agar. CC4533 grows best at temperatures ranging from 22°C-30°C. CC4533 cannot grow at 37°C on LB agar or on TAP agar medium. Preliminary spectrophotometric analyses of extracted yellow pigment of CC4533 strongly indicate that CC4533 produces β-carotenes. Preliminary results indicate growth medium type (LB Vs. TAP) and, temperature affects β-carotene production in CC4533.

0.1% acetate in the TAP medium can be substituted with alternative carbon sources (e.g. different sugars) to test if these alternative carbon sources can be used by bacteria for energy production. We monitored the ability of CC4533 to grow on **T**ris- **P**hosphate (TP) agar medium (lacks acetate) containing monosaccharide (glucose) and two disaccharides (sucrose and lactose). CC4533 was able to use these three sugars as the sole carbon source and was able to ferment these sugars to produce acid.

To determine the taxonomic identity of CC4533, we amplified the 16S rRNA gene partially by DNA polymerase Chain Reaction (PCR). We have submitted this partial 16S rRNA gene sequence to the NCBI GenBank in November 2019, with the definition: *Sphingobium yanoikuyae* strain PR86 variant; 16S ribosomal RNA gene, partial sequence (Accession number: MN633285.1). Our definition was based on the nearest relative identified by the NCBI-nucleotide BLAST analyses of the partial 16S rRNA gene sequence of CC4533 in 2019, which was *Sphingobium yanoikuyae* strain PR86 (Accession number: MN232173.1). In April 2020, we found another close relative of CC4533 in the NCBI database based on the partial 16S rRNA gene sequence, which was not detected in our earlier BLAST analyses in 2019. This new relative is *Sphingobium yanoikuyae* strain NRB095 (Accession number: MK543001.1). 16S rRNA partial gene sequences of these two *Sphingobium yanoikuyae* strains have identical: score, percentage of sequence identity, E-values and nucleotide changes in the C4 region at identical locations in the 16S rRNA gene, relative to that of CC4533.

*Sphingomonas sp.* and *Sphingobium sp.* are of particular interest due to their abilities to degrade cycloalkanes and polycyclic aromatic hydrocarbons (PAH) and polyhydroxyalkanoates (PHA) and their roles in environmental bioremediation ^17– 40^. Hence, we monitored the growth of CC4533 on TP-agar medium plates containing common biohazardous chemicals that are found in our natural environment. We tested the following chemicals: saturated hydrocarbons, cyclohexyl chloride, aromatic acid like benzoate, aromatic ester like phenyl acetate, polyesters (polyhydroxybutyrate, a biodegradable plastic) and poly-cyclic aromatic hydrocarbons. Growth analyses revealed that CC4533 is capable of utilizing these toxic organic compounds as the sole carbon and energy source. In future, we plan to sequence the whole genome of CC4533. This will provide important insights into the metabolic diversity of CC4533 and, will reveal the genes that are recruited by this bacterium to generate biochemical pathways for degradation of xenobiotics. In this article, we present our student-driven research on the isolation of CC4533 ( *Sphingobium yanoikuyae* strain PR86 variant) and its characterizations at the biochemical and molecular level.

## Methods

### Growth media and cultures

*Chlamydomonas* wild type strain 4A+ (CC- 4051 4A+ mt+) was maintained in the lab on TAP agar medium in dim light intensities (15-20 µmol m ^-2^s ^-1^) at 22°C (room temperature). Standard TAP medium recipe can be found at the website of Chlamydomonas Resource Center. Hutner’s trace element solution is an ingredient in the TAP medium. Hutner’s trace element recipe can also be found on the Chlamydomonas Resource Center website. Our lab’s TAP medium recipe is slightly different from the standard one and can be found at https://doi.org/10.17504/protocols.io.bgzujx6w ^41^. Liquid 4A+ cultures were grown in liquid TAP medium under low light (50-60 µmol m ^-2^s ^-1^) for 3 days on a New Brunswick Scientific Excella E5 platform shaker (Enfield, CT) at 150 rpm for aeration. CC4533 bacterial strain stock was maintained in the lab under dim light (15-20 µmol m ^-2^s ^-1^) at 22°C on either TAP or LB agar medium. Liquid cultures of CC4533 were grown in culture tubes in 3 mL of TAP medium at 22°C on a MaxQ420HP incubator shaker (Thermo Fisher Scientific, Waltham, MA) at 200 rpm for aeration. Light intensities were measured using a LI-250A light meter (LI-COR, Inc., Lincoln, NE). 

### Imaging

Images of all media plates used in the experiments were imaged with a Samsung Galaxy S5 cell phone camera. Image cropping and adjustments were made using Photos app in Windows 10. DNA gels were visualized and imaged with a Bio-Rad Molecular Imager Gel Doc XR+ (Bio-Rad, Hercules, CA).

### Antibiotic susceptibility test using the modified disc diffusion method

Antibiotics that were tested are: penicillin; neomycin, chloramphenicol and polymyxin B. Two doses (50 µg and 100 µg) of each of these four antibiotics were tested in the modified Kirby-Bauer (KB) disc diffusion antibiotic susceptibility tests. KB tests were performed on TAP-agar plates as described in Mitra *et al.* 2020 ^41– 43^. Antibiotic plates were incubated at 22°C. CC4533 plates were imaged after 3 days of incubation and *Chlamydomonas* plates were imaged after 4 days of incubation. Diameters of zone of inhibitions were measured using a ruler. Statistical analyses ^44^ and images of all antibiotic plates ^45^ are available as *Underlying data*.

### Data analyses

Standard deviations and means of the zones of inhibitions from the KB test were calculated using Microsoft Excel. Statistical analyses of the data from the KB disc diffusion test were performed using Microsoft Excels’ t-Test: Paired Two Sample for Means tool in the analysis ToolPak.

### Standard microbiological tests

1) *Growth at different temperatures*: CC4533 was streaked on fresh LB and TAP agar medium. Streaked CC4533 media plates were incubated at different temperatures: a) 22°C, b) 30°C, and c) 37°C, for 5 days and then imaged.

2) *Growth assays on MacConkey agar (MAC) and Mannitol Salt Agar (MSA)*: CC4533 was streaked on MAC and MSA plates purchased from Carolina Biological (Burlington, NC). Streaked plates were incubated at 30°C for 3 days. After the incubation period, plates were imaged to monitor CC4533 growth and pH change in the media.

3) *Testing CC4533’s ability to secrete hemolysins*: Growth was monitored at 30°C on tryptic soy agar medium plates containing 5% sheep agar (Carolina Biological; Burlington, NC) for a period of 72 hours. Images were taken after every 24 hours over a period of three days. Classification of hemolysis were assigned according to https://www.asm.org/Protocols/Blood-Agar-Plates-and-Hemolysis-Protocols. Images of all tryptic soy blood agar plates are available as *Underlying data* ^46^.

4) *Growth assays of CC4533 on Tris-Phosphate (TP)-phenol red-sugar- agar medium:* TP medium has all the ingredients of the TAP medium except the acetate ( https://doi.org/10.17504/protocols.io.bgzujx6w) ^41^. CC4533 was streaked on three types of TP-phenol red-agar medium containing 1% glucose or 1% sucrose or 1% lactose. pH of the TP medium was 7.2. TP-sugar medium plates were imaged after 5 days of growth at 22°C. Results were interpreted as described in Mitra *et al.* 2020 ^41^.

5) *Growth assays of CC4533 on TP agar media plates containing saturated hydrocarbons and aromatic compounds:* Growth assays were performed on TP-agar plates coated with different doses of cyclohexyl chloride, polyhydroxybutyrate, phenanthrene, naphthalene, benzoic acid, phenyl acetate and fresh and combusted 10W30 oil, using a modified technique as described in Mitra *et al.* 2020 ^41, 47^. CC4533 was streaked on the chemical-coated TP plates and incubated at 22°C and media plates were imaged after two weeks. Images of all media plates are available as *Underlying data* ^48^.

6) *Gram Staining, cytochrome c oxidase test and starch hydrolysis test:* Gram staining was performed using CC4533 cells from LB-agar medium plate, as described in Mitra *et al.* 2020 ^41^. Oxidase test was performed using CC4533 and a *Microbacterium sp.* cells from tryptic soy agar medium plates as described (with explanations) in Mitra *et al.* 2020 and ASM, Protocols ( https://www.asm.org/Protocols/Oxidase-Test-Protocol; https://microbeonline.com/oxidase-test-principle-procedure-and-oxidase-positive-organisms/) ^41^. For starch hydrolysis test, CC4533 and *Escherichia coli* were streaked on Mueller-Hinton agar medium plates purchased from Carolina Biological (Burlington, NC) and incubated for 48 hours at 30°C. Starch hydrolysis test was performed as described (with explanations) in Mitra *et al.* 2020 ^41^.

### Pigment analyses

LB-grown CC4533 cells and mashed baby carrots were used for pigment extraction. Pigments were extracted with 4 mL of 100% acetone by incubation in dark for 3.5 hours at room temperature. After incubation, the sample tubes were vortexed and then centrifuged at X 4000g for 5 minutes. The yellow supernatant from CC4533 and carrot sample tubes were collected and the cell pellet/tissue debris were discarded. The yellow supernatant was then filtered using a 5 mL syringe fitted to a nylon membrane filters with a cut-off of 0.45 µm. Pigments were analyzed by direct absorbance measurements using the wavelength scan program ranging from 400-600 nm in a Beckman Coulter DU 730 Life science UV/Vis spectrophotometer (Brea, CA). The carrot supernatant absorbance scan was used as a reference overlay against the CC4533 measured scan. To detect carotenoids in the samples, absorption peaks in the 420-490 nm region were monitored ( https://assets.publishing.service.gov.uk/media/57a08cbae5274a31e00013d4/tech02.pdf) ^49, 50^.

### Genomic DNA isolation and PCR amplification of the partial 16S rRNA gene

Genomic DNA was isolated from CC4533 using Qiagen’s blood and cell culture DNA mini kit (Qiagen, Valencia, CA) according to the protocol given in the technical manual. Purity of the isolated genomic DNA and its concentration were measured using a Nanodrop 2000 spectrophotometer (Thermo Fisher Scientific, Waltham, MA). Genomic DNA quality was determined by visualization of the genomic DNA after it was separated by DNA agarose gel electrophoresis.

16S rRNA gene forward (16SF) and reverse (16SR) PCR primers were designed based on primer sequences given in article by Klindworth *et al*. (2013) ^51^. Primer sequences, details about PCR cycling conditions, specific DNA polymerase used in the PCR, separation and visualization of PCR samples can be found in the article by Mitra *et al.* 2020 ^41^.

### PCR product cloning and DNA sequencing

The partial 16S rRNA genomic PCR product was extracted and purified from the DNA agarose gel using the QIAquick Gel Extraction Kit (Qiagen, Valencia, CA). The purified PCR product was cloned as described in Mitra *et al.* 2020 ^41^. One clone was sequenced using Sanger Dideoxy sequencing at the UC Berkeley DNA Sequencing Facility. Chromas Lite and nucleotide BLAST program were used to analyze the partial 16S rRNA gene sequences. Raw electropherogram files and DNA sequence text files are available as *Underlying data* ^52^.

## Results

Antibiotic sensitivity data of the bacterial strain CC4533 (Sphingobium yanoikuyae strain PR86 variant; GenBank Accession number: MN633285.1) and Chlamydomonas reinhardtii strain 4A+. CC4533 Data S1 contain information about means of the zones of inhibitions of the bacterial strain CC4533 (*Sphingobium yanoikuyae* strain PR86 variant) and *Chlamydomonas* and corresponding standard deviations. CC4533 Data S2 file contains information on statistical analyses of the zones of inhibitions of the bacterial strain CC4533 (*Sphingobium yanoikuyae* strain PR86 variant) and *Chlamydomonas*, induced by four antibiotics. Antibiotics used are: penicillin, chloramphenicol, neomycin and polymyxin B. Three biological replicates were used for the generation of the data. Two different doses of antibiotics were used: 50 and 100 micrograms of each antibiotics. Data was collected for CC4533 (*Sphingobium yanoikuyae* strain PR86 variant) after 3 days of growth at room temperature. Data was collected for *Chlamydomonas* after 4 days of growth at room temperature (22C). Click here for additional data file. Copyright: © 2020 Mitra M et al. 2020

Images of antibiotic plates of the bacterial strain CC4533 (Sphingobium yanoikuyae PR86 strain variant partial 16S rRNA sequence; GenBank Accession # MN633285.1) and green micro-alga Chlamydomonas from the antibiotic susceptibility disc diffusion tests. The file contains 16 images of antibiotic plates used for the antibiotic susceptibility tests using the disc diffusion method for *Chlamydomonas* and the bacterial strain, CC4533 (*Sphingobium yanoikuyae* PR86 strain variant). Antibiotics tested are: penicillin, chloramphenicol, polymyxin B and neomycin. Two different doses of antibiotics were used: 50 and 100 micrograms of each antibiotics.** On the antibiotic plates, the filter paper disc on the left contains the antibiotic and that on the right contains sterile water (control)**. CC4533 (*Sphingobium yanoikuyae* PR86 strain variant) plates were imaged after 3 days of growth and *Chlamydomonas *plates were imaged after 4 days of growth at room temperature (22C).**** **** ****
Copyright: © 2020 Mitra M et al. 2020

Growth of the bacterial strain CC4533 (Sphingobium yanoikuyae PR86 strain variant partial 16S rRNA sequence; GenBank Accession # MN633285.1) and Staphylococcus aureus on Tryptic Soy agar medium containing 5% sheep blood. Six figures in this file show the growth of the bacterial strain CC4533 (Sphingobium yanoikuyae PR86 strain variant) and *Staphylococcus aureus* on Tryptic Soy agar medium plates containing 5% sheep blood (Carolina Biological, Burlington, NC) over a period of 3 days at 30C. Plates were imaged after every 24 hours. *Sphingobium yanoikuyae* PR86 strain variant did not show any alpha hemolysis after 24 hours. Dark brown coloration on the blood agar medium around the growth of *Sphingobium yanoikuyae* PR86 strain variant was observed after 48 hours of growth. This brown coloration became more pronounced after 72 hours of growth. *S. aureus *is beta hemolytic and showed clear zones around its growth on blood agar after 24 hours of growth. Click here for additional data file. Copyright: © 2020 Mitra M et al. 2020

Tests using Tris-Phosphate medium (TP) to see if hydrocarbons, aromatic compounds and polyhydroxyalkanoates can be used by the bacterium CC4533 (Sphingobium yanoikuyae PR86 strain variant, partial 16S rRNA sequence; GenBank Accession # MN633285.1) as the sole carbon source. The file contains 21 images of TP (Tris-Phosphate) medium plates containing different alternative carbon sources. Bacterium CC4533 (*Sphingobium yanoikuyae* PR86 strain variant) was streaked on these chemical plates to test if CC4533 can utilize these chemicals as the sole carbon source for energy and growth. 1% stocks of the following chemicals were tested: cyclohexyl chloride, phenanthrene, napthalene, benzoic acid and phenyl acetate. 2% (v/v) stocks of fresh and used car motor oil 10W30 were also tested. Chemical doses used are given in mL in the file name. medium plates were imaged after two weeks of growth at room temperature (22C) Click here for additional data file. Copyright: © 2020 Mitra M et al. 2020

16S rRNA partial gene sequences of the bacterial strain CC4533 (Sphingobium yanoikuyae PR86 strain variant partial 16S rRNA sequence; GenBank Accession # MN633285.1). Dataset contains 2 abi extension files and corresponding sequence text files obtained from partial sequencing of the 16S rRNA gene of CC4533 strain (*Sphingobium yanoikuyae* PR86 strain variant). There are 2 electropherogram files and corresponding text files of DNA sequences in the dataset. The 16S rRNA gene was partially amplified from CC4533 and cloned in the TOPO-TA vector (Thermo Scientific Fisher) for DNA sequencing. Sanger dideoxy sequencing technology was used for DNA sequencing. Click here for additional data file. Copyright: © 2020 Mitra M et al. 2020

### CC4533 can use acetic acid for growth

**Figure 1.**
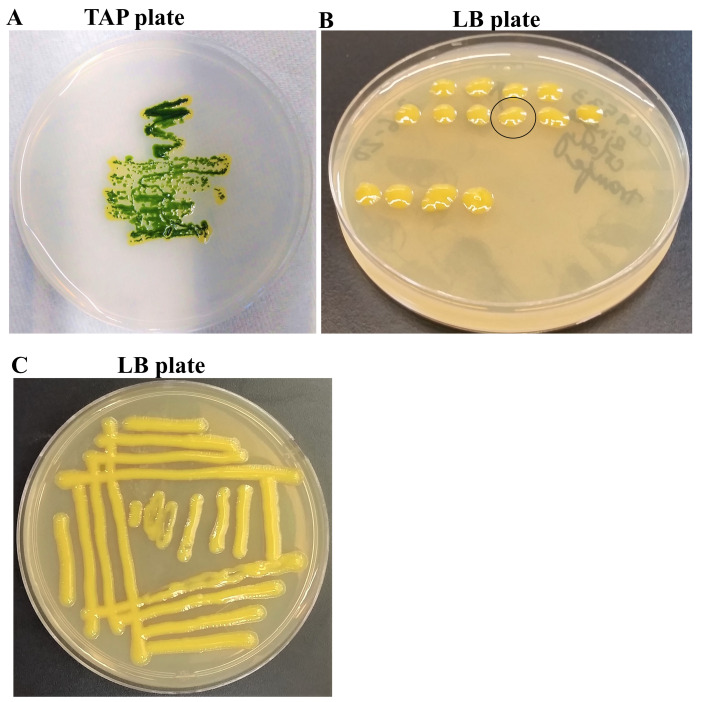
Isolation of a bacterial strain from a contaminated *Chlamydomonas* CC4533 strain TAP-agar medium plate. ( **A**) **T**ris- **A**cetate- **P**hosphate (TAP)-agar medium plate showing bacterial contamination of a *Chlamydomonas* strain, CC4533 at room temperature (22°C). ( **B**) A LB ( **L**ysogeny **B**roth)-agar medium plate showing some of the single colonies of CC4533 that were picked. Colony # 28 outlined by the black circle was selected for culture stock maintenance and further analyses. ( **C**) LB-agar medium plate of purified CC4533 strain. Culture plate shown in ( **C**) was imaged after 5 days of growth at 22°C.

We found a yellow pigmented-bacterial contamination on a TAP medium plate of a *Chlamydomonas* wild type strain, CC4533, at our laboratory ( Figure 1A). We purified the bacterial strain from the contaminated TAP-agar medium plate by picking single colonies on fresh TAP agar medium. We picked 40 single colonies and transferred these colonies to fresh LB agar medium. Colony # 28 was selected for our studies ( Figure 1B). Colony # 28 stock was maintained in the lab on LB agar medium at 22°C ( Figure 1C). We named this bacterial strain as CC4533, as the bacterium was isolated from the *Chlamydomonas* strain, CC4533 TAP-agar medium plate. CC4533 bacterial strain can use acetate as a carbon/energy source. Hence it was able to grow on the TAP-agar medium ( Figure 1A).

### CC4533 is resistant to polymyxin B, penicillin and chloramphenicol and is sensitive to neomycin

We performed two different sets of experiments for testing antibiotic-sensitivity. In the first experiment, we determined the antibiotic-sensitivity of CC4533 and *Chlamydomonas* 4A+ wild type strain to identify a suitable antibiotic and the required dose that would inhibit growth of CC4533 but will not affect the growth of *Chlamydomonas*. In the second set of experiments, we streaked *Chlamydomonas* and bacterium CC4533 together on the TAP-agar medium plate containing the antibiotic at the proper dose that we determined based on the results obtained from the first experiment set. We used two different doses (50 µg and 100 µg) of each of the following four antibiotics: penicillin, chloramphenicol, neomycin and polymyxin B. Mean diameter of the zone of inhibition for each antibiotic dose with the standard deviations are shown in Table 1. Detailed statistical analyses of the data from three biological replicates (each of which had three internal replicates) are available as *Underlying data* ^44^. Images of TAP antibiotic-agar plates from the KB experiments are available as *Underlying data* ^45^.

**Table 1.** Mean diameters of zones of inhibitions obtained using the disc-diffusion antibiotic susceptibility test. Zones of inhibitions induced by four different antibiotics were studied for *Chlamydomonas reinhardtii* and the bacterial strain, CC4533. Grey and white rows represent 50 µg and 100 µg dose of each antibiotics applied on the filter paper discs, respectably. Three biological replicates with three internal replicates were used to calculate the mean and standard deviations shown in the table. Statistical analyses ^44^ and images of all antibiotic plates ^45^ are available as *Underlying data*.

Antibiotic	*C. reinhardtii*	**CC4533**
**Penicillin**	0 mm ± 0	0 mm ± 0
	0 mm ± 0	0 mm ± 0
**Polymyxin *B***	8.5 mm ± 0.2	8.7 mm ± 0.1
	9.6 mm ± 0.4	10.4 mm ± 0.4
**Neomycin**	9.5 mm ± 0.5	13.5 mm ± 0.5
	11.1 mm ± 0.1	14.8 mm ± 0.2
**Chloramphenicol**	0 mm ± 0	0 mm ± 0
	0 mm ± 0	0 mm ± 0

At 50 µg dose, bacterium CC4533 was more sensitive to polymyxin B than *Chlamydomonas* and, both *Chlamydomonas* and CC4533 were sensitive to the 100 µg dose of polymyxin B ( Table 1; *Underlying data* ^44, 45^). Statistical analyses supported that the 50 µg dose of polymyxin B is not very effective in inhibiting growth of CC4533, without drastically affecting *Chlamydomonas* growth ( *Underlying data* ^44, 45^). Both *Chlamydomonas* and CC4533 were sensitive to 100 µg (1000 units) dose of polymyxin B, but there was a significant statistical difference between the polymyxin B-sensitivity of *Chlamydomonas* and CC4533 at the 100 µg dose ( *Underlying data* ^44, 45^; Table 1). Both CC4533 and *Chlamydomonas* were resistant to the two doses of penicillin namely, 50 µg (82.5 IU units) and 100 µg (165 IU units) as no zone of inhibition was visible for both doses ( *Underlying data* ^44, 45^; Table 1).

*Chlamydomonas* and CC4533 were both resistant to the 50 µg and 100 µg of chloramphenicol as no zone of inhibition was obtained in the KB test ( *Underlying data* ^44, 45^; Table 1). *Chlamydomonas* was less sensitive to both 50 µg and 100 µg dose of neomycin than bacterium CC4533 ( *Underlying data* ^44, 45^; Table 1) and the sensitivity was statistically highly significant ( *Underlying data* ^44, 45^; Table 1). Taken together, our KB test results showed that neomycin would be the best antibiotic choice to eliminate CC4533 contamination of *Chlamydomonas*. Polymyxin B at a higher dose (see results) could be the second choice of antibiotic for minimizing CC4533 contamination, but *Chlamydomonas* growth will be also affected at the higher dose of polymyxin B.

**Figure 2.**
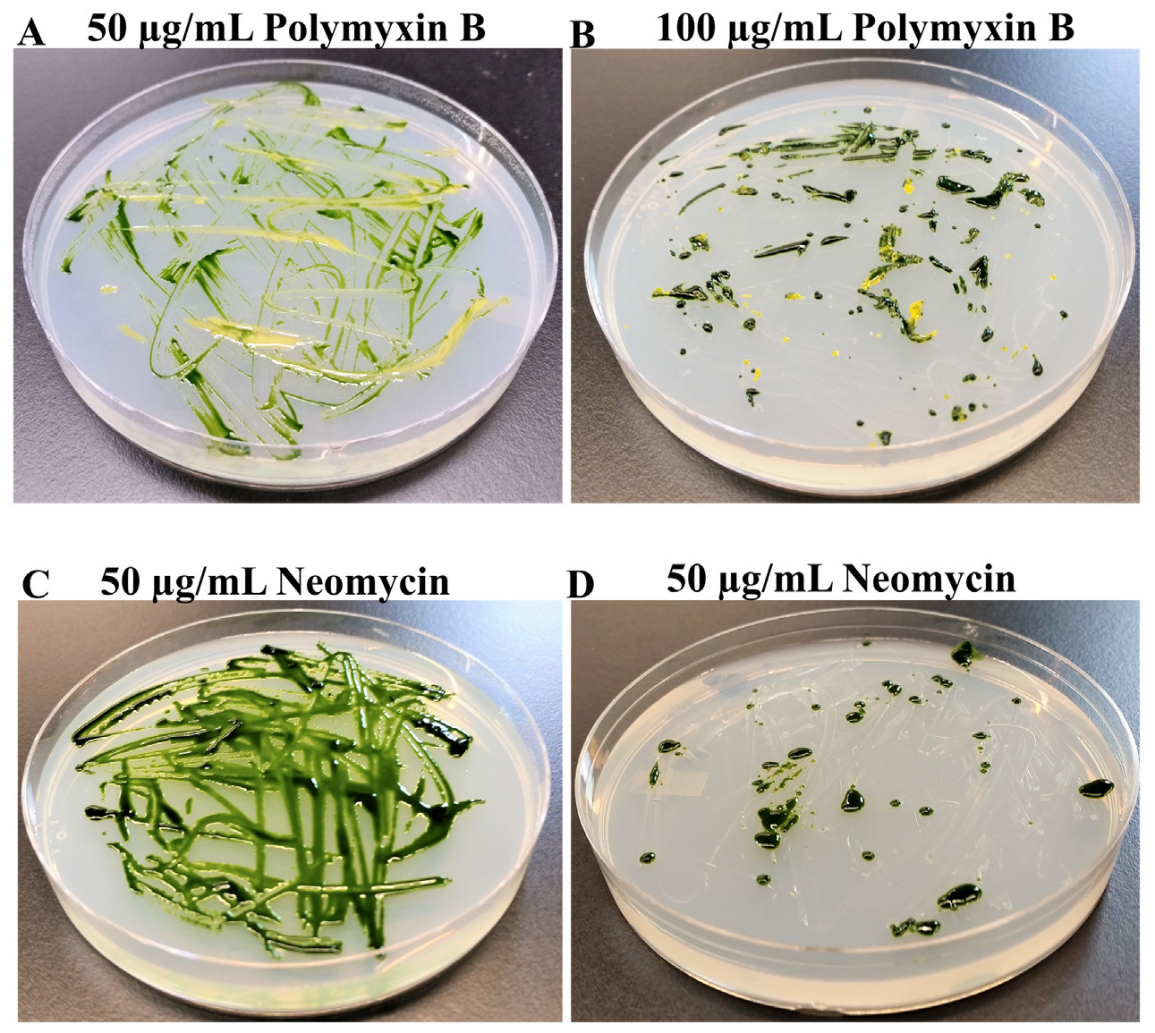
Testing the efficacy of polymyxin B and neomycin in eliminating CC4533 contamination. ( **A**) *Chlamydomonas* strain 4A+ and CC4533 strain streaked on TAP-agar plate containing 50 µg of polymyxin B/mL of medium. ( **B**) *Chlamydomonas* strain 4A+ and CC4533 strain streaked on TAP-agar plate containing 100 µg polymyxin B/mL of medium. ( **C**) *Chlamydomonas* strain 4A+ and CC4533 strain TAP-agar plate containing 50 µg of neomycin/mL of medium. ( **D**) *Chlamydomonas* strain 4A+ and CC4533 strain streaked on TAP-agar plate containing 100 µg neomycin/mL of medium. TAP-agar antibiotic plates were incubated at room temperature for 2.5 weeks before they were imaged.

In the second experiment, we tested combined growth of CC4533 and the wild type *Chlamydomonas* strain 4A+ on TAP agar plates containing 50 µg and 100 µg of polymyxin B and neomycin per mL of the TAP medium ( Figure 2). CC4533 was able to grow along with *Chlamydomonas* on TAP media plates containing 50 µg and 100 µg of polymyxin B per mL of the TAP medium ( Figure 2A and Figure 2B). CC4533 did not grow on TAP plates containing 50 µg and 100 µg of neomycin per mL of the TAP medium ( Figure 2C and Figure 2D). *Chlamydomonas* grew slowly on the TAP medium plate containing 100 µg neomycin/mL of TAP but grew at a normal rate on the TAP medium plate containing 50 µg neomycin/mL of TAP ( Figure 2C and Figure 2D). Hence neomycin at a concentration of 50 µg/mL in the TAP medium is most effective in inhibiting the growth of bacterium CC4533 on *Chlamydomonas* culture plates without affecting the growth of *Chlamydomonas*. 

### Gram staining

**Figure 3.**
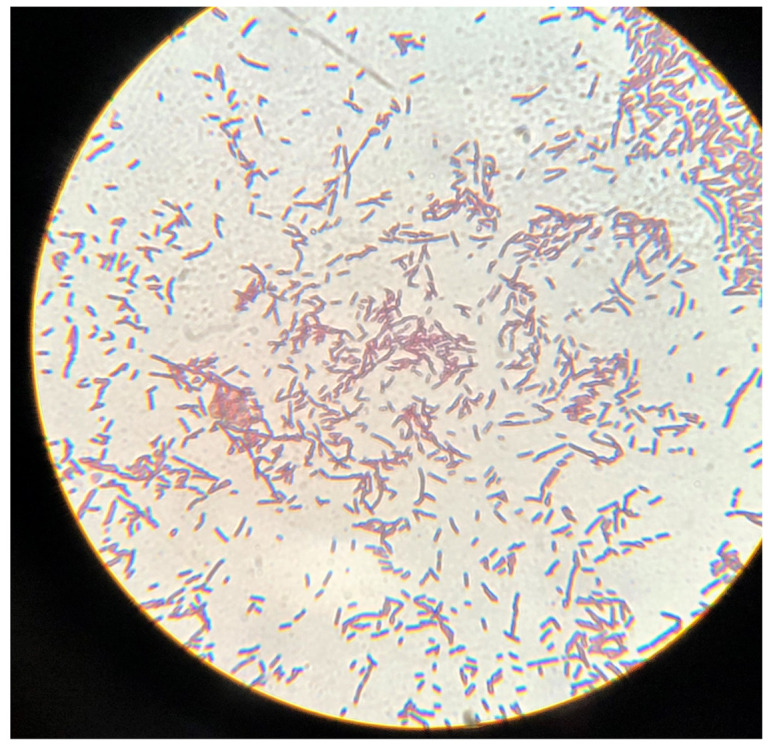
Gram-stained CC4533 imaged under 100X magnification. CC4533 cells from a LB-agar medium plate was used for gram-staining. Gram-stained cells were visualized and imaged under an oil immersion lens of a bright-field microscope.

Gram staining of CC4533 revealed that CC4533 is a Gram-negative bacillus. Cells are straight long rods which join to form chains ( Figure 3).

### C4533 grows best at 22-30°C but fails to grow at 37°C

**Figure 4.**
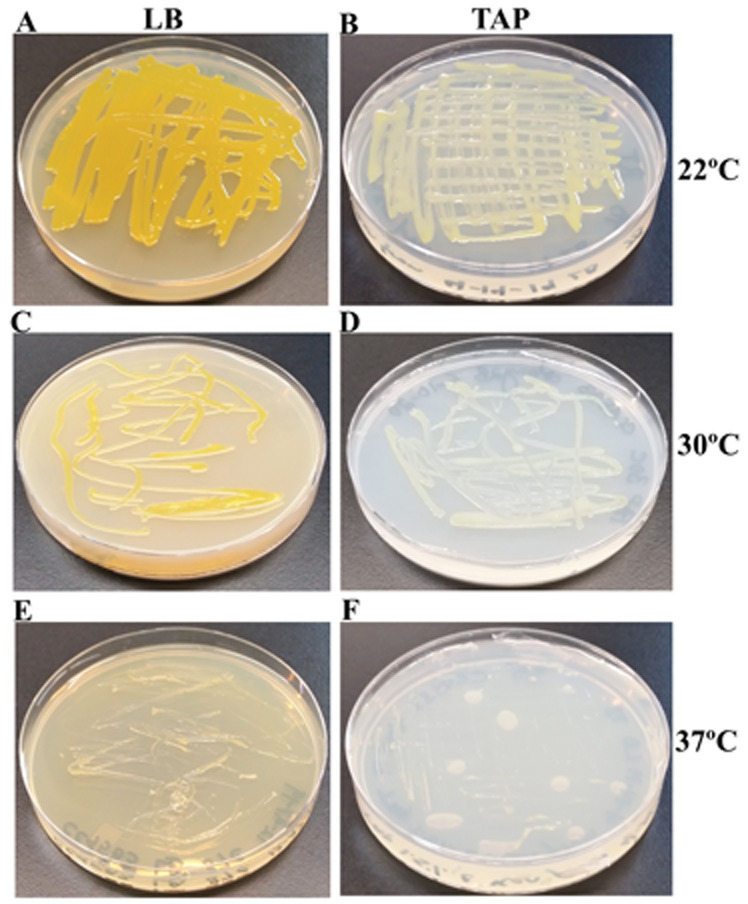
Growth of CC4533 growth on TAP- and LB-agar medium plates at different temperatures. ( **A**) Growth on LB-agar medium plate at room temperature (22°C). ( **B**) Growth on TAP-agar medium plate at 22°C. ( **C**) Growth on LB-agar medium plate at 30°C. ( **D**) Growth on TAP-agar medium plate at 30°C. ( **E**) Growth on LB-agar medium plate at 37°C. ( **F**) Growth on TAP-agar medium plate at 37°C. Culture plates were imaged after 5 days of growth.

We have monitored the growth of CC4533 on LB-agar and TAP-agar over 5 days at different temperatures namely 22°C, 30°C and 37°C. CC4533 grew well on LB and TAP-agar at 22°C and 30°C but could not grow at 37°C ( Figure 4). Yellow pigmentation of CC4533 was visibly reduced on TAP medium compared to that on the LB medium. Growth at 30°C reduced pigment accumulation compared to that at 22°C on both LB and TAP medium ( Figure 4).

### CC4533 can utilize and ferment different sugars for growth

**Figure 5.**
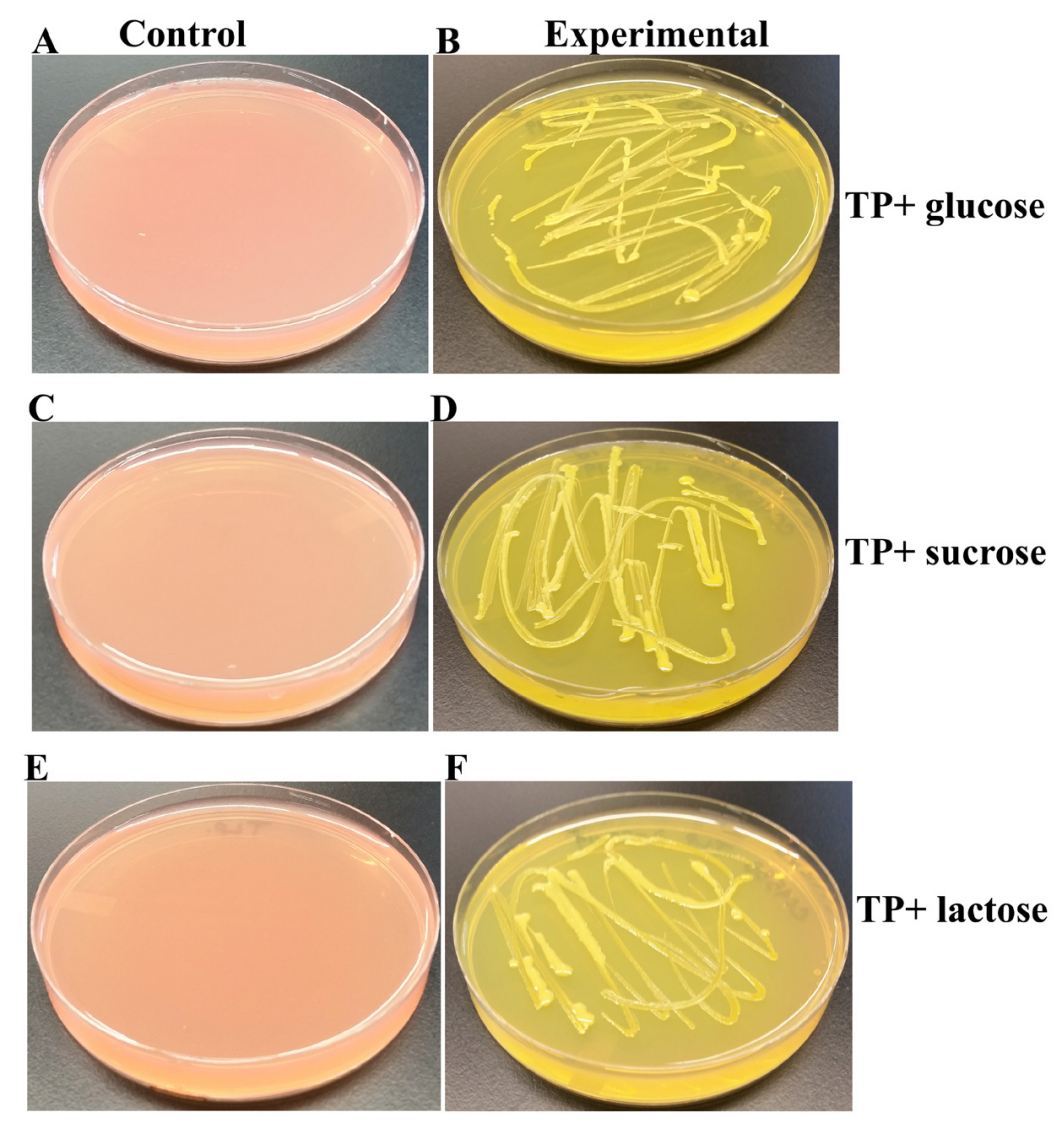
Growth of CC4533 on TP- sugar agar medium. ( **A**) Control TP + 1% glucose agar medium plate with phenol red as a pH indicator. ( **B**) CC4533 on TP +1% glucose agar medium plate with phenol red as a pH indicator. ( **C**) Control TP +1% sucrose agar medium plate with phenol red as a pH indicator. ( **D**) CC4533 growth on TP +1% sucrose agar medium plate with phenol red as a pH indicator. ( **E**) Control TP +1% lactose agar medium plate with phenol red as a pH indicator. ( **F**) CC4533 growth on TP +1% lactose agar medium plate with phenol red as a pH indicator. Culture plates were imaged after 5 days of growth at room temperature. CC4533 fermented glucose, sucrose and lactose as evident from the yellow color of phenol red.

We grew CC4533 on TP (lacks the carbon source, acetate) agar medium containing three different types of sugars (glucose, sucrose and lactose) and the pH indicator, phenol red ( Figure 5). Figure 5A, Figure 5C and Figure 5E represent control TP + 1% glucose, TP + 1% sucrose and TP + 1% lactose, plates, respectively. CC4533 grew very well on all sugar supplemented TP agar plates ( Figure 5). It fermented sugars on all TP-sugar medium plates to produce acid which lowered the pH in the TP medium. The drop in pH, changed the color of phenol red from light red color to a yellow color ( Figure 5B, Figure 5D and Figure 5F).

### CC4533 fails to grow on MacConkey Agar (MAC) and on Mannitol Salt Agar (MSA)

**Figure 6.**
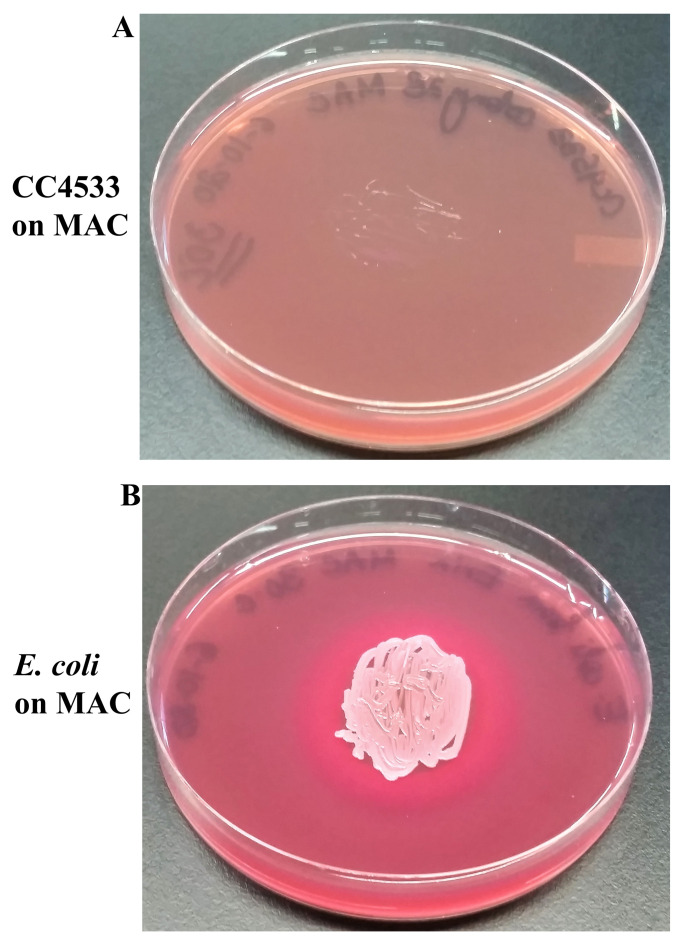
Growth of CC4533 on MacConkey Agar. ( **A**) 72 hours-growth of CC4533 on MacConkey Agar medium plate. **(B**) 72 hours growth of *Escherichia coli* on MacConkey Agar medium plate. CC4533 fails to grow on MacConkey agar medium plate. *E. coli* appears pinkish because it ferments lactose to acid, which causes the neutral red pH indicator to turn red. The dark opaque pink haze on the medium around the *E. coli* growth is the bile precipitation in acidic environment. Media plates were incubated at 30°C.

CC4533 was unable to grow on MAC and *E. coli,* a Gram-negative enteric bacterium ( Figure 6A), was able to grow on MAC. MAC contains lactose as the carbon source. The pink color of *E. coli* on MAC plate, indicated that it can ferment the sugar lactose present in the MAC medium to produce acid as the acidic pH (pH below 6.8) changed the color of neutral red to pink ( Figure 6B). The drop in the pH in the MAC medium around the growth of *E. coli*, precipitated the bile salts out of the MAC medium which caused a hazy pink zone to develop around the *E. coli* growth ( Figure 6B). CC4533 can use lactose as a carbon source ( Figure 5F). CC4533 cannot grow on MAC because it is sensitive to bile salts and crystal violet in the MAC medium ( Figure 6A). Our results indicate that CC4533 is a non-enteric bacterium unlike *E. coli*.

**Figure 7.**
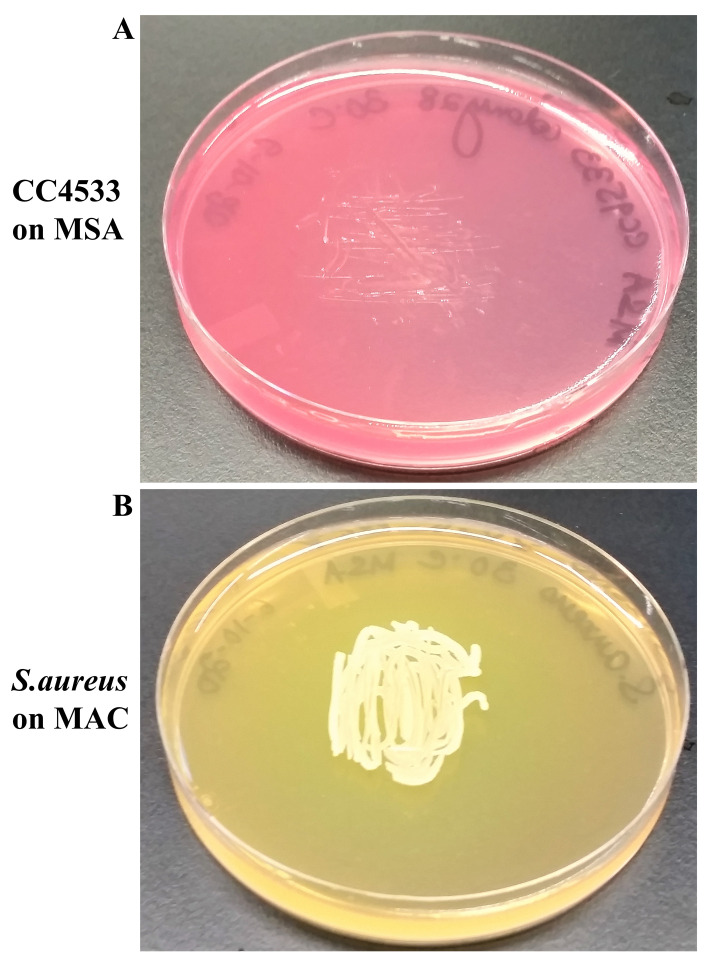
CC4533 is salt-sensitive. ( **A**) 72 hours-growth of CC4533 on Mannitol Salt Agar medium plate. ( **B**) 72 hours growth of *Staphylococcus aureus* on Mannitol Salt Agar medium plate. CC4533 fails to grow on Mannitol Salt Agar medium plate. *S. aureus* grows on Mannitol Salt Agar and can ferment mannitol. Media plates were incubated at 30°C.

CC4533 fails to grow on MSA ( Figure 7A). *Staphylococcus aureus* grew on MSA and fermented the sugar-alcohol, mannitol, present in the MSA medium to produce acid, which changed the phenol red’s color from red to yellow ( Figure 7B). Our results show that CC4533 is salt-sensitive as MSA contains about 7.5-10% of NaCl, which inhibits growth of many gram-negative bacteria and, allows selection of high salt-tolerant gram-positive bacterium like *Staphylococcus*.

### CC4533 is alpha-hemolytic

**Figure 8.**
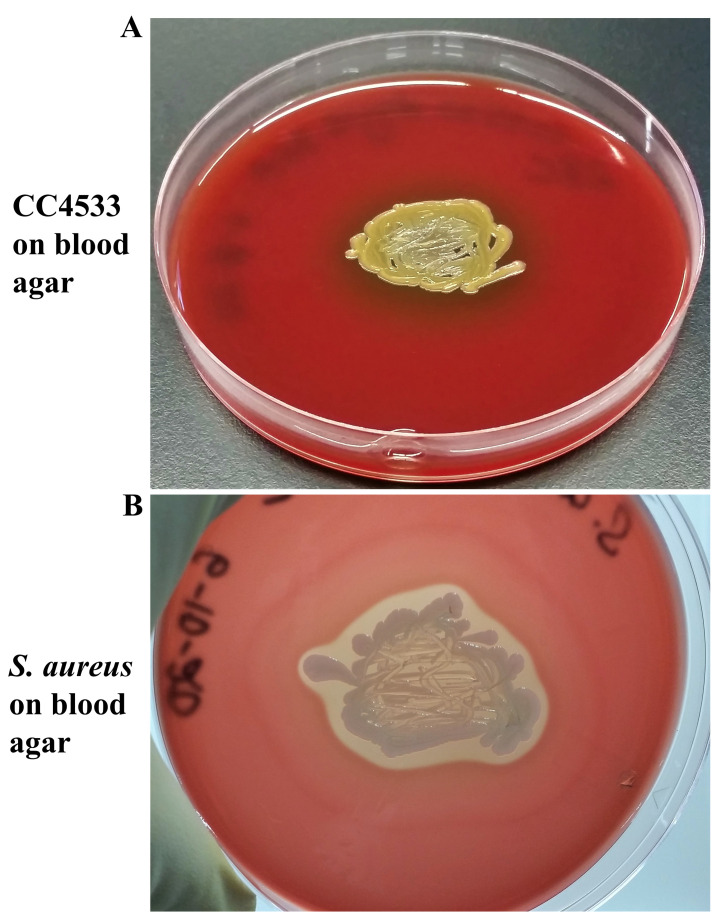
CC4533 is alpha hemolytic. ( **A**) 72-hours growth of CC4533 on tryptic soy-blood agar plate. ( **B**) 72 hours growth of *Staphylococcus aureus* on tryptic soy-blood agar plate. Media plates were incubated at 30°C. Images of 24 hours- and 48-hours growth of both strains are available as *Underlying data* ^46^.

CC4533 did not show any hemolysis or discoloration of blood agar medium after 24 hours growth ( *Underlying data* ^46^). After 48 hours, a dark brown discoloration around the cell growth was observed ( *Underlying data* ^46^), which became more pronounced after 72 hours of growth ( Figure 8A), indicating CC4533 is alpha-hemolytic. Figure 8B shows that *S. aureus* is beta-hemolytic as complete hemolysis can be seen around the *S. aureus* growth after 24 hours of growth. After 72 hours, the clearing on the blood agar medium plate is more pronounced. Images of tryptic soy blood agar plates are available as *Underlying data* ^46^ and in Figure 8B.

### CC4533 is unable to hydrolyze starch

**Figure 9.**
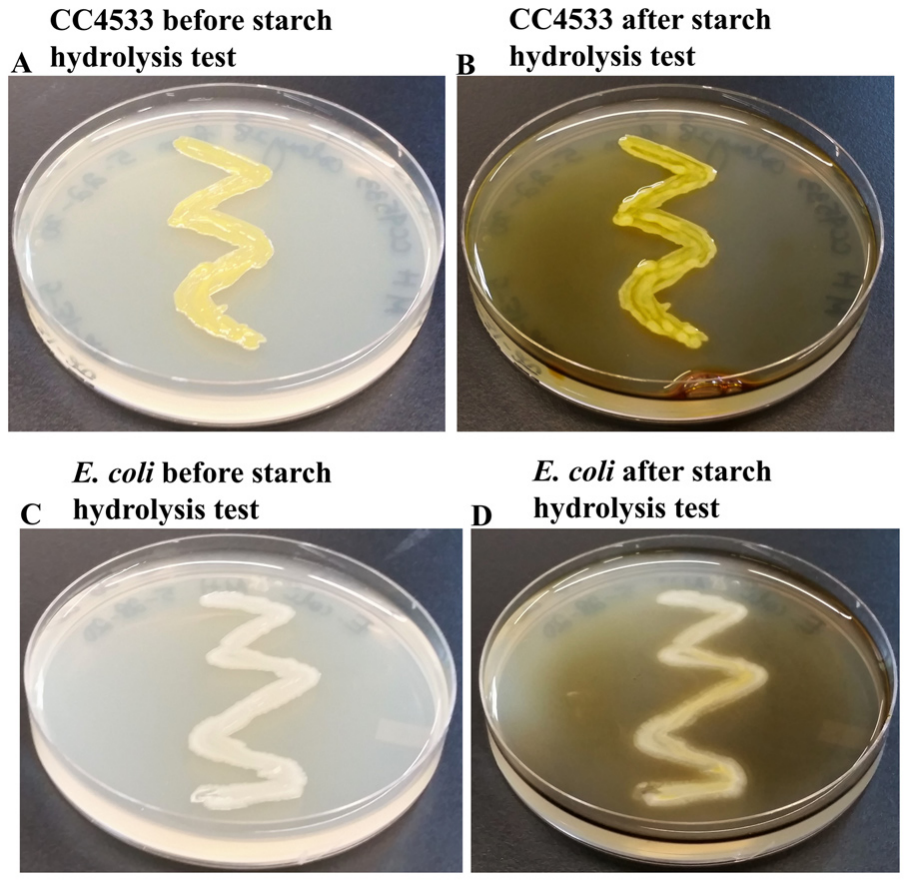
CC4533 is starch hydrolysis-negative. ( **A**) 48 hours-growth of CC4533 at 30°C on Mueller-Hinton medium which contains 0.15% starch. ( **B**) CC4533 Mueller-Hinton plate shown in ( **A**) was treated with Gram iodine. ( **C**) 48 hours-growth of *E. coli* at 30°C on Mueller-Hinton medium which contains 0.15% starch. ( **D**) *E. coli* Mueller-Hinton plate shown in ( **C**) treated with Gram iodine. *E. coli* and CC4533 fail to hydrolyze starch on Mueller-Hinton medium as there are no visible clear zones around the bacterial growth after gram iodine treatment. The brown color of the medium upon Gram iodine treatment occurs because of the reaction of starch in the medium with iodine.

We performed starch hydrolysis test on CC4533 and *E. coli* on Mueller-Hinton agar medium, which contains 0.15% starch ( Figure 9). Background and scientific basis of the starch hydrolysis test can be found in Mitra *et al.* 2020 ^41^. Both CC4533 ( Figure 9B) and *E. coli* ( Figure 9D) cannot hydrolyze starch as there was no visible clear zone around the growth on the Mueller Hinton agar. Iodine used in the starch hydrolysis test reacted with the starch present in Mueller-Hinton media to produce a brown/blue color. The results show that both bacterial strains do not secrete the enzymes α-amylase and oligo-1, 6-glucosidase to hydrolyze amylose and amylopectin (starch). We did not have a starch hydrolysis-positive strain in our lab to use as a positive control in this experiment.

### CC4533 uses cytochrome c oxidase in the respiratory electron transport chain

**Figure 10.**
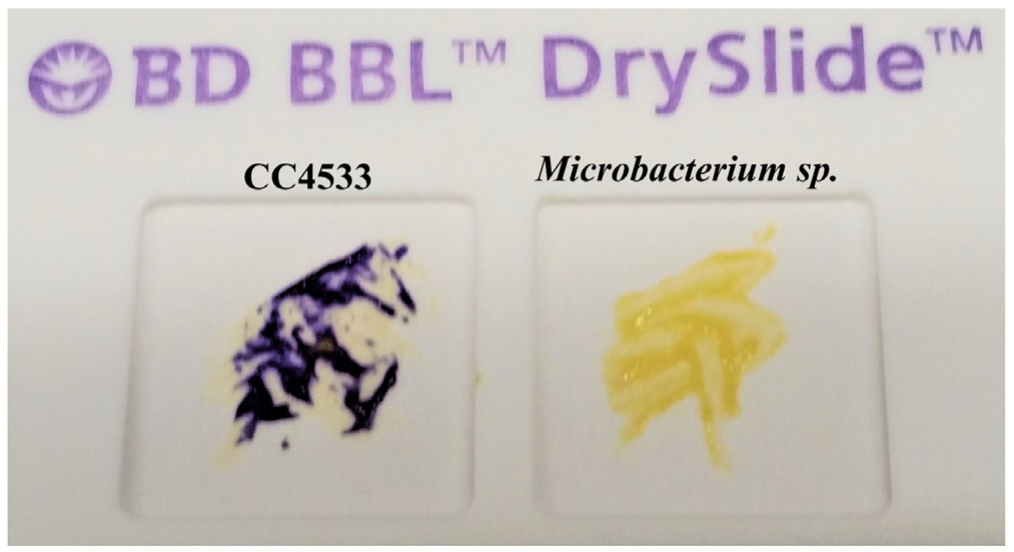
CC4533 is oxidase-positive. Cells of CC4533 (on the left) and *Microbacterium sp.* (on the right) streaked on a disposable slide containing a film coated with oxidase reagent (tetramethyl-p-phenylenediamine dihydrochloride). Image of the slide was taken after 10 seconds of the application of the cells on the slide. CC4533 is cytochrome c oxidase-positive as cytochrome c oxidase, if present, oxidizes the oxidase reagent on the film to form purple colored-indophenols. *Microbacterium sp.,* a yellow-pigmented bacterium, is oxidase-negative and fails to form the purple-colored product within 10 seconds.

Aerobic, facultative anaerobic or microaerophilic bacteria that uses cytochrome c oxidase in the electron transport chain associated with cellular respiration can be identified by the oxidase test. We conducted the oxidase test on CC4533 and on a yellow pigmented- *Microbacterium sp.*, using a disposable slide that contains a film coated with oxidase reagent tetramethyl-p-phenylenediamine (TMPD). CC4533 oxidized the TMPD to indophenols, a purple colored product, within 5–10 seconds. CC4533 is oxidase-positive ( Figure 10; left). *Microbacterium sp.* is oxidase-negative as it failed to change the color of TMPD to purple within 5–10 seconds. ( Figure 10; right). We grow CC4533 under aerobic conditions in our lab ( Figure 1– Figure 9). Our results show that CC4533 is an aerobic bacterium that uses cytochrome c in the respiratory electron transport chain.

### CC4533 synthesizes β-carotene

**Figure 11.**
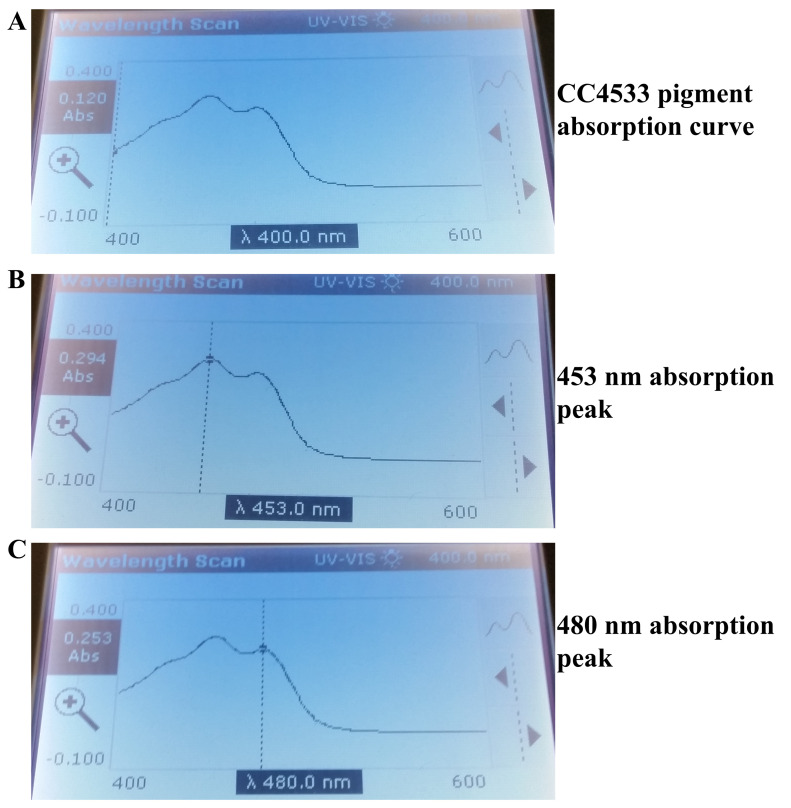
Absorption spectrum of pigments extracted from CC4533. ( **A**). Absorption curve of the yellow pigment extract of CC4533. ( **B**) Absorption peak of pigment extract at 453 nm. ( **C**) Absorption peak of pigment extract at 480 nm. CC4533 cells were harvested for pigment extraction from a LB agar medium plate maintained under low light. Absorption maxima were measured using the wavelength scan program with the wavelength range of 400-600 nm in a UV-Vis spectrophotometer.

It is known that many bacteria accumulate carotenoids, which gives them orange to yellow pigmentation. As CC4533 is yellow-pigmented, we tested for the presence of carotenoids in CC4533, grown under normal room light (30-40 µmol m ^-2^s ^-1^). We monitored the absorption spectrum of the acetone-extracted CC4533 pigment using wavelength scan program ranging from 400- 600 nm in a UV-Vis spectrophotometer. Carotenoids absorb strongly in the visible light range from 400-495 nm, with absorption peaking near 450 nm ( https://assets.publishing.service.gov.uk/media/57a08cbae5274a31e00013d4/tech02.pdf) ^49, 50^. We found two major absorption peaks in the 400-500 nm region of the spectrum ( Figure 11) ^49, 50^. The absorption peak with the absorbance reading (0.294) is at 453 nm and the one with the absorbance reading of 0.253 is at 480 nm ( Figure 11B and Figure 11C). β-carotene shows two major absorption peaks that range between 451 nm - 454 nm and 477 nm - 480 nm ^49, 50^. The observed two absorption peaks in CC4533 pigment extract strongly indicates the presence of β-carotene in CC4533.

**Figure 12.**
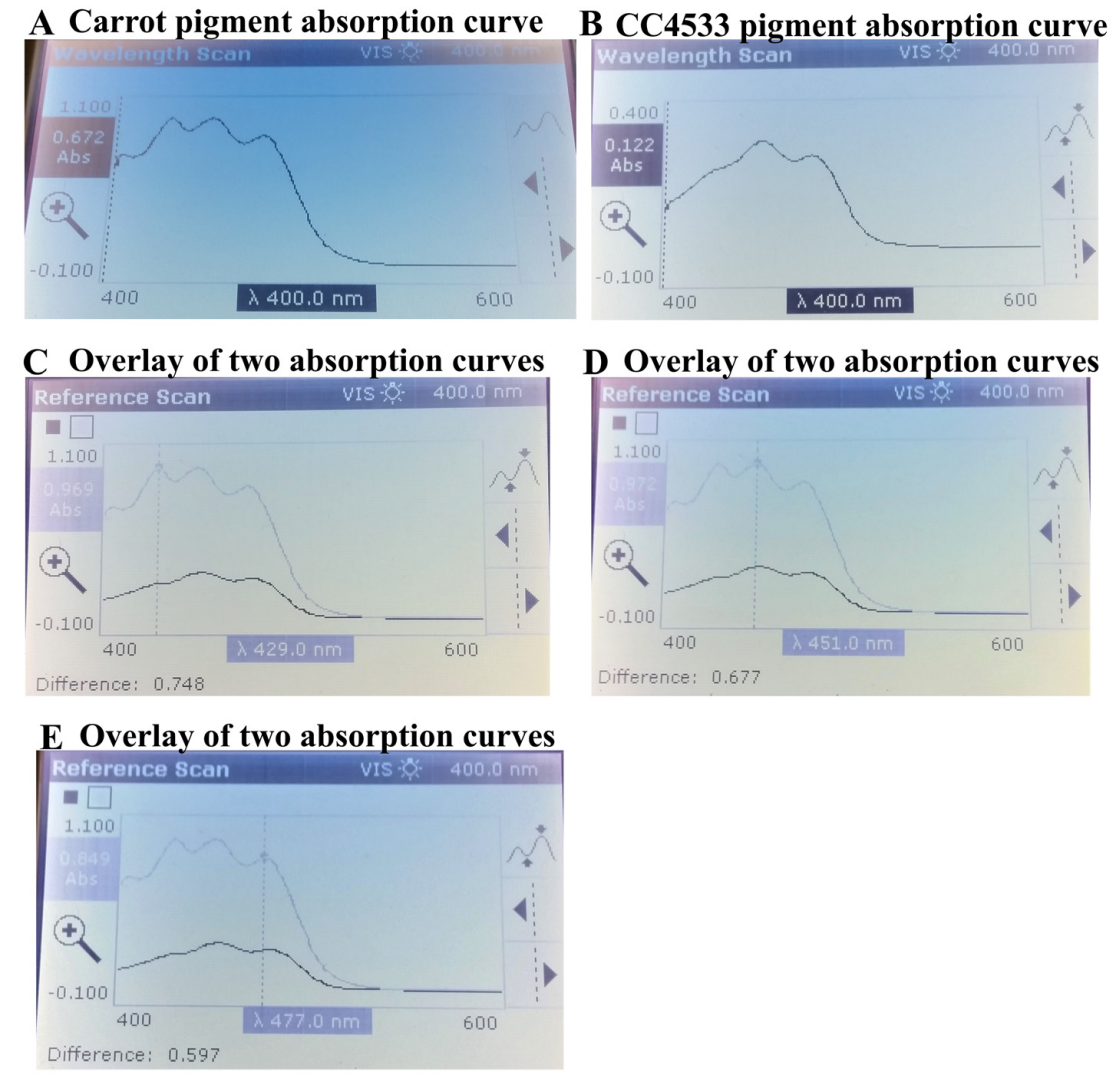
Comparative analyses of absorption spectrums of pigments of carrot and CC4533. ( **A**) Absorption curve of extracted pigments from baby carrots ( **B**) Absorption curve of the extracted yellow pigment from CC4533. ( **C**) Measured scan of CC4533 pigment absorption (black plot) against the overlay of the reference scan of carrot extract (light blue plot). The absorption curve shows the carotene absorption peak at 429 nm and the corresponding difference in absorbance readings between the two samples. ( **D**) Measured scan of CC4533 pigment absorption (black plot) against the overlay of the reference scan of carrot extract (light blue plot). The absorption curve shows the β-carotene absorption peak at 451 nm and the corresponding difference in absorbance readings between the two samples. ( **E**) Measured scan of CC4533 pigment absorption (black plot) against the overlay of the reference scan of carrot extract (light blue plot). The absorption curve shows the β-carotene absorption peak at 477 nm and the corresponding difference in absorbance readings between the two samples. CC4533 cells from a LB agar medium plate maintained under low light was used for pigment extraction. Absorption maxima were measured using the wavelength scan program with wavelength range of 400-600 nm in a UV-Vis spectrophotometer.

80% of the carotenoids in carrots is β-carotenes ^53, 54^. We extracted pigments from carrots and measured its absorption peaks ( Figure 12). We used the carrot pigments’ absorption curve as a reference scan overlay against the measured scan of CC4533 pigment extract ( Figure 12). We found that the extracted- carrot pigments exhibited three major peaks that are representative of β-carotene absorption at 429 nm, 451 nm and 477 nm ( https://assets.publishing.service.gov.uk/media/57a08cbae5274a31e00013d4/tech02.pdf; Figure 12A, Figure 12C, Figure 12D; Figure 12E). Two of these peaks (451 nm and 477 nm) are very close to that of absorption peaks (453 nm and 480 nm) of CC4533 pigment extract ( Figure 11B, Figure 11C, Figure 12B, Figure 12D and Figure 12E). The 429 nm peak in the pigment extract of carrot was not prominently visible in the pigment extract of CC4533 ( Figure 12C). Our preliminary data strongly indicates the presence of β-carotene in CC4533.

### Partial 16S rRNA gene sequence of CC4533 has 99.55% sequence identity with that of Sphingobium yanoikuyae

**Figure 13.**
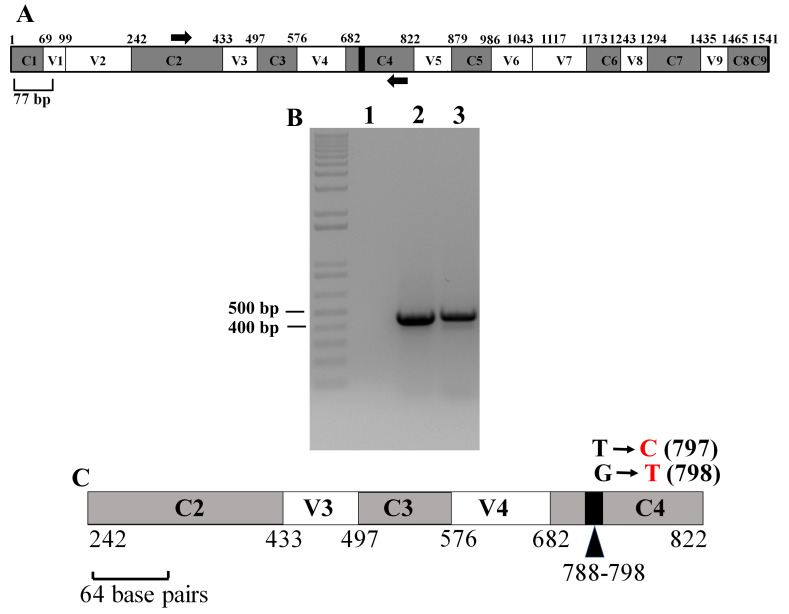
PCR amplification of the 16S rRNA partial gene sequence of CC4533 and NCBI-BLAST analyses. ( **A**) A schematic diagram showing the conserved and hypervariable regions in the 16S rRNA gene. The interspersed conserved regions (C1–C9) are shown in gray, and the hypervariable regions (V1–V9) are depicted in white. The black box within the C4 region represents 11 nucleotides (788 -798 base pairs) that are invariant in bacteria. PCR primers are shown in thick black arrows. Forward primer is in the C2 region and the reverse primer is in the C4 region. The figure is based on the 16S rRNA gene sequence of *E. coli*. ( **B**) A DNA agarose gel showing the results of PCR with the primers shown in Figure 13A. Lane 1 represents PCR with water (zero DNA control) and Lane 2 shows the PCR product from a bacterial strain LMJ ^41^ isolated by our lab and, Lane 3 shows the CC45333 PCR product. 1kb plus DNA ladder was used as a DNA molecular size ladder on the agarose gel. ( **C**) A schematic diagram showing the nucleotide changes in CC4533 in the 16S rRNA region spanning the C2 and C4 regions in comparison to the best NCBI- BLAST hit (score of 802; E-value 0 and percent identity of 99.55%): *Sphingobium yanoikuyae* strain PR86 16S ribosomal RNA gene, partial sequence (GenBank Accession #: MN232173.1). Black nucleotides show the native nucleotides in the BLAST hit *Sphingobium yanoikuyae* strain PR86 that were substituted by the depicted red nucleotides in CC4533 16S rRNA gene sequence. The black bold numbers within the parenthesis beside the nucleotides show the specific nucleotide position where the nucleotide changes have occurred. Nucleotide positions shown in the figures have been assigned according to that of the 16S rRNA gene sequence of *E. coli*. DNA sequencing data is available as *Underlying data* ^52^.

The full length 16S rRNA gene is 1541 bp long ( Figure 13A; based on *E. coli* 16S rRNA gene). There are nine hypervariable (V1-V9) and nine conserved regions (C1- C9) in the 16S rRNA gene ^55– 57^. 11 nucleotides (788-798) within the C4 conserved region are totally conserved in bacteria ^58^. This super-conserved region is represented in Figure 13 as a black box within the C4 region ^58^. Forward and reverse PCR primers are represented by black arrows in the schematic of the 16S rRNA gene ( Figure 13A). PCR amplification of the partial 16S rRNA gene of CC4533 generated an amplicon of approximately 460 bp in size ( Figure 13B). The amplicon shown in Figure 13B was sequenced to determine the nearest relative of CC4533.

In fall 2019, NCBI-nucleotide BLAST analyses identified the nearest relative of CC4533 as *Sphingobium yanoikuyae* strain PR86 (GenBank Accession #: MN232173.1) based on the partial 16S ribosomal RNA gene sequence. This hit has a score of 802; zero E-value and percent identity of 99.55%. Figure 13C shows two nucleotide substitutions (transitions) that are present in CC4533 16S rRNA partial gene sequence relative to that in *Sphingobium yanoikuyae* strain PR86. These two nucleotide substitutions are in the 11 bp super-conserved sub-region within C4 region ( Figure 13C). We deposited in November 2019, the partial 16S rRNA sequence of CC4533 in GenBank with the definition: *Sphingobium yanoikuyae* strain PR86 variant, 16S ribosomal RNA gene, partial sequence (Accession number: MN633285.1). DNA sequencing data of the 16S rRNA gene of CC4533 are available as *Underlying data* ^52^. In 2020, NCBI-BLAST analyses revealed another close relative of CC4533: *Sphingobium yanoikuyae* strain NRB095 (Accession number: MK543001.1) based on the partial 16S rRNA gene sequence. Both strains of *Sphingobium yanoikuyae* detected by our NCBI-BLAST analyses had identical scores, E-values and sequence identity (including the same nucleotide substitutions at the identical locations within the 16S rRNA gene) when their partial 16S rRNA gene sequences were compared against that of CC4533.

### CC4533 AKA Sphingobium yanoikuyae strain PR86 variant can use biohazardous saturated hydrocarbons and aromatic compounds as the sole carbon source for growth

*Sphingobium sp.* are known to use alkanes, polycyclic aromatic hydrocarbons (PAH), polyhydroxyalkanoates like polyhydroxybutyrate (PHB) and other aromatic compounds as alternative carbon sources for growth ^17– 40^. Hence, we tested if CC4533 can utilize saturated hydrocarbons, PAH, other biohazardous aromatic compounds and PHB as carbon sources for growth. These growth analyses were performed as described in Mitra *et al.* 2020 ^41^. Different doses of the chemicals that were tested can be also found in Mitra *et al.* 2020 and are also available as *Underlying data* ^48^.

**Figure 14.**
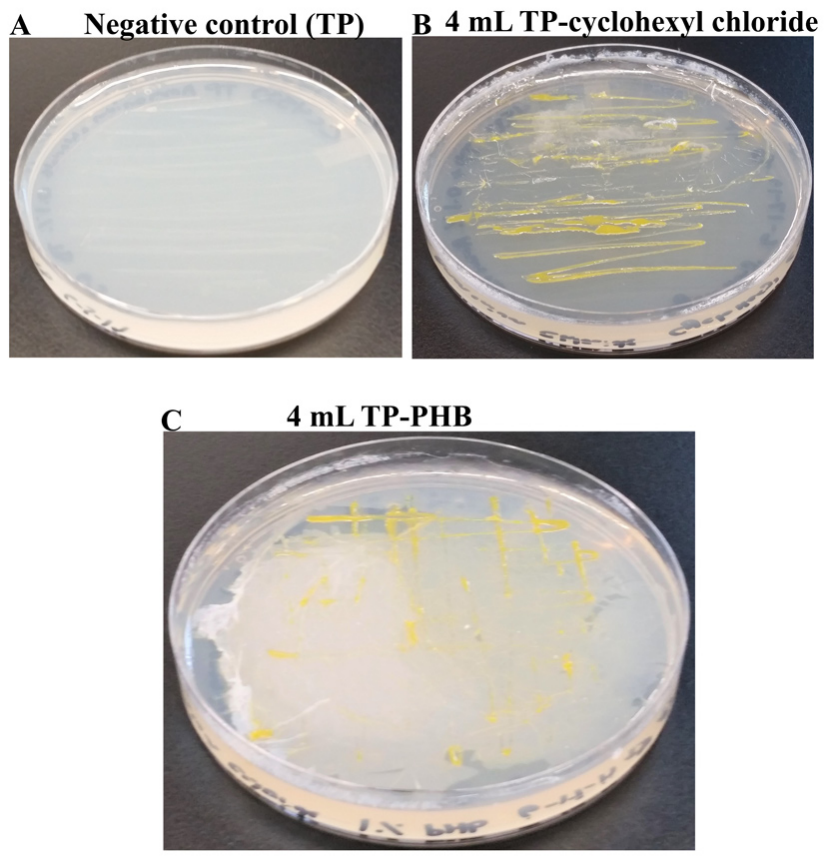
Growth of CC4533 using cycloalkane and polyhydroxybutyrate as the sole carbon source. Tris- Phosphate (TP) agar medium plates shown in Figure 14B & Figure 14C were coated with either cyclohexyl chloride or polyhydroxybutyrate. CC4533 was streaked on the negative control TP-agar medium plate and on the hydrocarbon-coated TP-agar medium plates. After 2 weeks of growth at room temperature, media plates were imaged. ( **A**) TP-agar medium plate streaked with CC4533. CC4533 does not grow on TP medium as it lacks a carbon source. ( **B**) CC4533 growth on TP-agar medium plate coated with 4 mL of 1% cyclohexyl chloride diluted with chloroform. ( **C**) CC4533 growth on TP-agar medium plate coated with 4 mL of 1% polyhydroxybutyrate.

CC4533 was streaked on a TP-agar medium plate, which lacks a carbon source, as a negative control to show that CC4533 does not grow on a TP medium plate in the absence of a carbon source ( Figure 14A). CC4533 was able to grow on TP medium containing all doses of 1% cyclohexyl chloride (a mono chlorinated hydrocarbon) ( Figure 14B) and 1% PHB (belongs to the class of polyhydroxyalkanoates that are used as bio-derived and biodegradable plastics) ( Figure 14C). Our results show that CC4533 can use both these organic compounds as sole carbon sources for growth.

**Figure 15.**
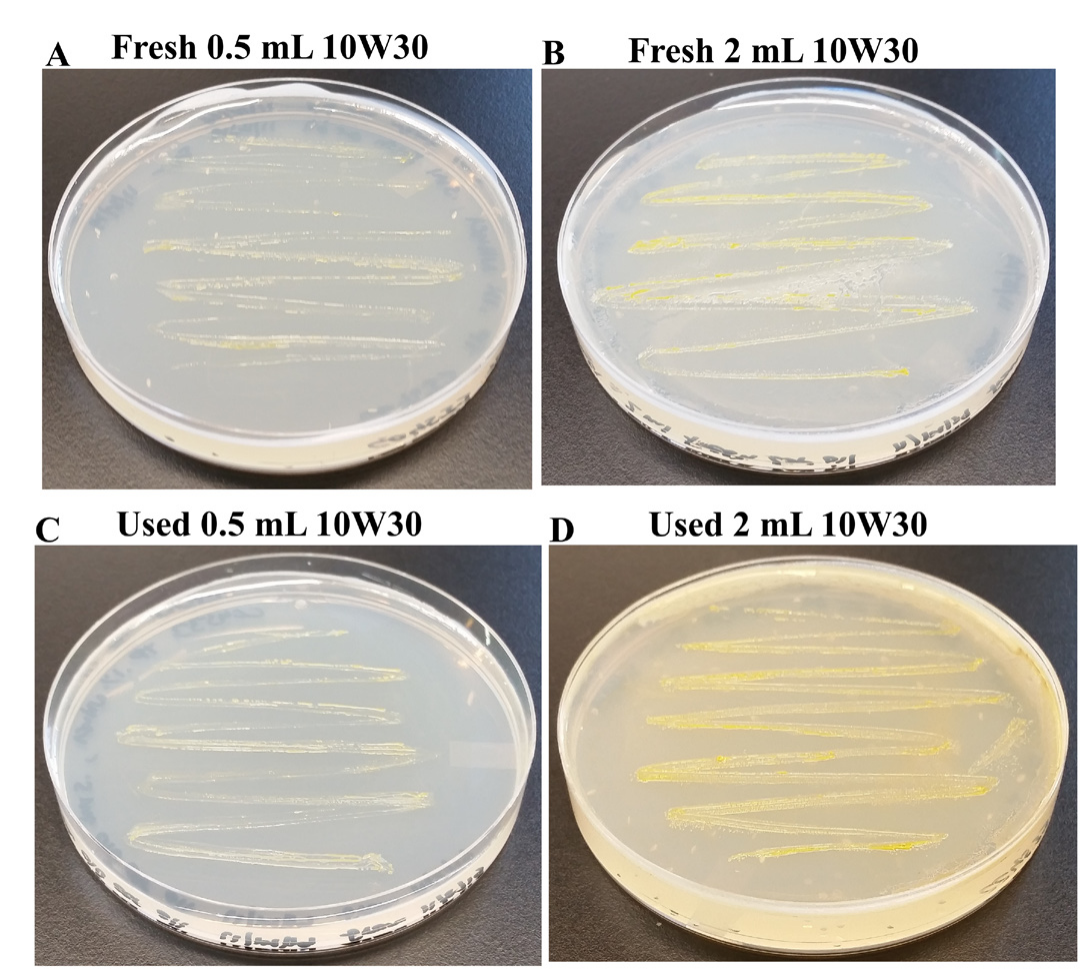
Growth of CC4533 using car motor oil as the sole carbon source. Tris- Phosphate (TP) agar medium plates shown in Figures 15A-15D were coated with different does of fresh and combusted car motor oil. CC4533 was streaked on the motor oil-coated TP-agar media plates shown in Figures 15A-15D. After 2 weeks of growth at room temperature, media plates were imaged. ( **A**) CC4533 growth on TP-agar medium plate coated with 0.5 mL of 2% fresh 10W-30 car motor oil. ( **B**) CC4533 growth on TP-agar medium plate coated with 2 mL of 2% fresh 10W-30 car motor oil. ( **C**) CC4533 growth on TP-agar medium plate coated with 0.5 mL of 2% combusted 10W-30 car motor oil. ( **D**) CC4533 growth on TP-agar medium plate coated with 2 mL of 2% combusted 10W-30 car motor oil. TP-agar medium plate (lacks a carbon source) shown in Figure 14A served as the negative control for this experiment. Images of plates with different doses of car motor oil are available as *Underlying data* ^48^.

Motor oils contain petroleum-based hydrocarbons which contain between 18 and 34 carbon atoms per molecule, poly-alpha olefins or their mixtures in different ratios. We monitored the growth of CC4533 on TP medium containing 2% (v/v) fresh 10W30 car motor oil ( Figure 15A–Figure B) and 2% (v/v) combusted 10W30 car motor oil ( Figures 15C–D). C4533 grew on TP-agar containing all doses of 2% fresh ( Figure 15A–B) and 2% combusted 10W30 motor oil ( *Underlying data* ^48^; Figure 15C–D), indicating that it can utilize petroleum-derived hydrocarbons as carbon sources.

**Figure 16.**
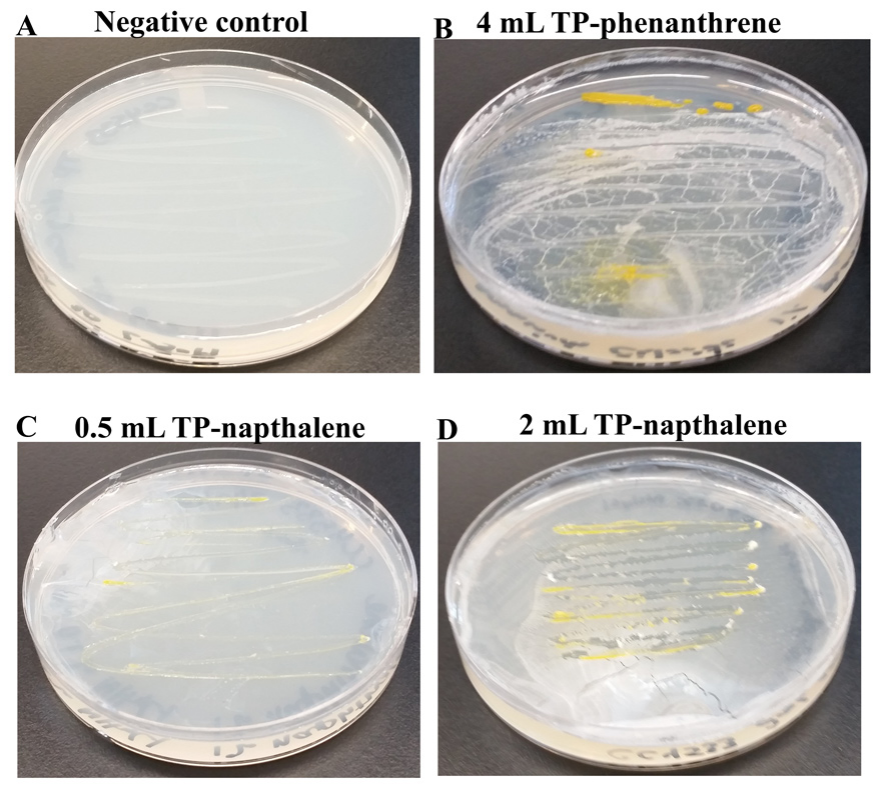
Growth of CC4533 using polycyclic aromatic hydrocarbons as the sole carbon source. TP medium plates shown in Figures 16B-16D were coated with polycyclic aromatic hydrocarbons (PAH). CC4533 was streaked on the negative control TP-agar medium plate and on the PAH-coated TP-agar medium plates. After 2 weeks of growth at room temperature, media plates were imaged. ( **A**) TP control plate streaked with CC4533. CC4533 does not grow on TP plate as it lacks a carbon source. ( **B**) CC4533 growth on TP plate coated with 4 mL of 1% phenanthrene dissolved in chloroform. ( **C**) CC4533 growth on TP plate coated with 0.5 mL of 1% naphthalene dissolved in chloroform. ( **D**) CC4533 growth on TP plate coated with 2 mL of 1% naphthalene dissolved in chloroform. Images of plates with different doses of aromatic hydrocarbons are available as *Underlying data* ^48^.

Phenanthrene is a PAH composed of three fused benzene rings. Napthalene is a PAH consisting of two fused benzene rings. CC4533 was streaked on a TP medium plate which lacks a carbon source and this plate served as the negative control in the experiment ( Figure 16A). We monitored the growth of CC4533 on TP medium containing 1% phenanthrene ( Figure 16B) and 1% naphthalene ( Figures 16C–D). CC4533 grew on TP-agar containing all doses of 1% phenanthrene and 1% naphthalene ( *Underlying data* ^48^; Figures 16B–D).

**Figure 17.**
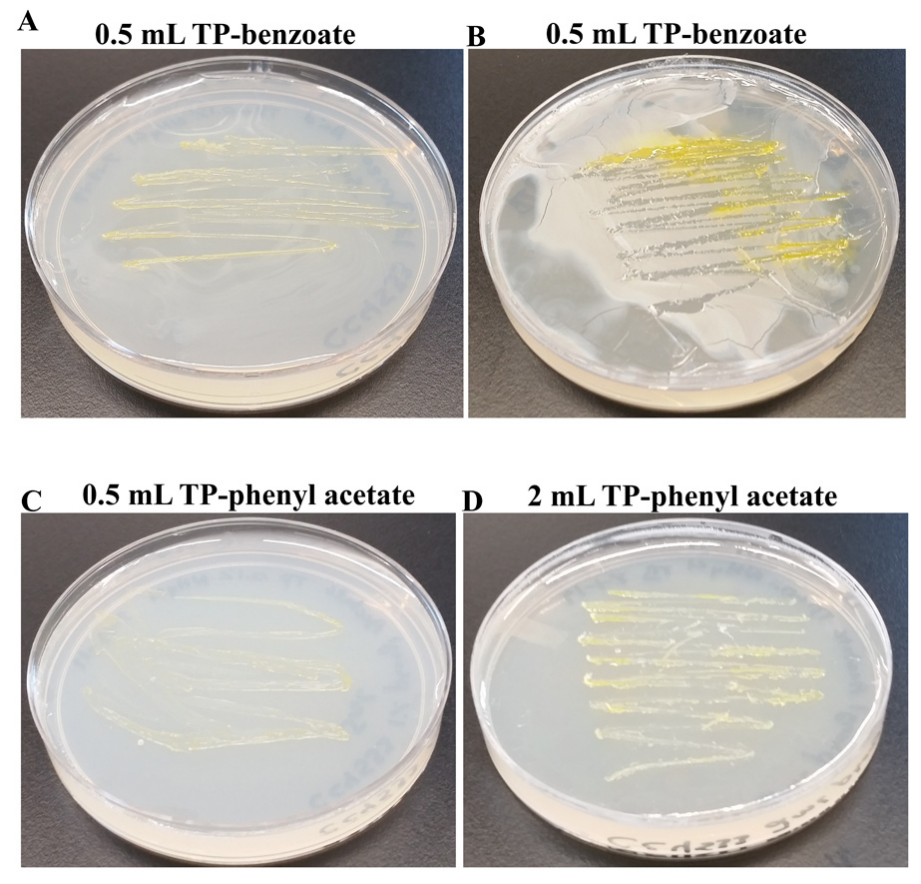
Growth of CC4533 using biohazardous aromatic compounds as the sole carbon source. TP medium plates shown in Figures 17A-17D were coated with aromatic compounds. CC4533 was streaked on the aromatic compound-coated TP-agar medium plates. After 2 weeks of growth at room temperature, media plates were imaged. ( **A**) CC4533 growth on TP plate coated with 0.5 mL of 1% benzoate dissolved in chloroform. ( **B**) CC4533 growth on TP plate coated with 2 mL of 1% benzoate dissolved in chloroform. ( **C**) CC4533 growth on TP plate coated with 0.5 mL of 1% phenyl acetate dissolved in chloroform. ( **D**) CC4533 growth on TP plate coated with 2 mL of 1% phenyl acetate dissolved in chloroform. TP-agar medium plate (lacks a carbon source) shown in ( Figure 16A served as the negative control for this experiment. Images of plates with different doses of aromatic compounds are available as *Underlying data* ^48^.

Benzoic acid is an aromatic carboxylic acid with a single benzene ring. Benzoic acid and sodium benzoate are commonly used as food preservatives and these preservatives are major environmental pollutants. Phenyl acetate is the ester of phenol and acetyl chloride. Phenylacetate is a common environmental pollutant and is a central intermediate in the pathways that degrade aromatic chemicals (e.g. phenylalanine, phenylacetaldehyde, lignin-related phenylpropane units, environmental contaminants like styrene and ethylbenzene) ^59^. CC4533 was able to utilize all tested doses of benzoic acid ( Figures 17A–B) and phenyl acetate ( Figures 17C–D) in the TP medium as the sole carbon sources ( *Underlying data* ^48^).

## Discussion

CC4533 is a mesophilic, yellow pigmented, Gram-negative rod ( Figure 1– Figure 4). It can ferment glucose, sucrose and lactose ( Figure 5). It is a non-enteric, salt-sensitive, alpha hemolytic, starch hydrolysis-negative, oxidase-positive bacterium ( Figure 6– Figure 10). NCBI-nucleotide BLAST analyses of the partial 16S rRNA gene sequence of CC4533 revealed that the best match is to that of *Sphingobium yanoikuyae* strain PR86 (GenBank Accession #: MN232173.1) and to that of *Sphingobium yanoikuyae* strain NRB095 (Accession number: MK543001.1) with a sequence identity of 99.55% and zero E-value, indicating strongly that CC4533 is a new strain of *Sphingobium yanoikuyae.* Whole genome sequencing can confirm if CC4533 is a new species of *Sphingobium* or a new strain of *Sphingobium yanoikuyae* (see discussion end).

The family Sphingomonadaceae currently includes the genera *Sphingomonas*, *Sandaracinobacter*, *Blastomonas*, *Novosphingobium*, *Sphingobium*, *Sphingopyxis*, *Sandarakinorhabdus*, *Sphingosinicella*, *Stakelama*, *Sphingomicrobium*, *Sphingorhabdus*, *Parasphingopyxis*, and *Zymomonas* ^60^. The type genus *Sphingomonas* was dissected into four genera based on clustering in the 16S rRNA gene sequence phylogeny and differences in the major polyamine and the 2-hydroxy fatty acid patterns: the genus *Sphingomonas* sensu stricto and the three new genera *Novosphingobium*, *Sphingobium*, and *Sphingopyxis* ^60^.

*Sphingobium sp*. are chemoorganotrophs and have been isolated from soils, freshwater and marine habitats, activated sludge, phyllosphere and rhizosphere. The isolation source for *Sphingobium yanoikuyae* strain PR86 (GenBank Accession #: MN232173.1) is sunflower endo-phyllosphere and that for *Sphingobium yanoikuyae* strain NRB095 (Accession number: MK543001.1) is surface disinfested root of the tropical Koronivia grass *Brachiaria humidicola*. There are various examples of plant growth-promoting organisms within the Sphingomonadaceae family but the genus *Sphingobium* is largely limited to members that degrade xenobiotic compounds ^61– 65^. Exception is the *Sphingobium sp.* strain AEW4, isolated from the rhizosphere of the beach grass, *Ammophila breviligulata* ^61^. This strain has plant growth promoting properties via production of siderophores and indole-3-acetic acid and induces root growth ^61^. *Sphingobium paulinellae sp.* nov. and *Sphingobium algicola sp.* nov are isolated from *Paulinella chromatophora*, a freshwater filose amoeba with photosynthetic endosymbionts (chromatophores) of cyanobacterial origin ^66^. In nature, *C. reinhardtii* is predominantly found in temperate, nutrient-rich, cultivated field soils in Northern America and Japan ^67, 68^. We isolated CC4533 ( *Sphingobium yanoikuyae* strain PR86 variant) in our laboratory from TAP agar medium plates of the micro-green-alga *C. reinhardtii*.

CC4533 is resistant to the β-lactam group of drugs like penicillin, and to the broad-spectrum antibiotic, chloramphenicol ( Table 1). It is resistant to both 50 µg and 100 µg doses of the cationic antimicrobial polypeptide, polymyxin B, which is one of the most effective drugs against Gram-negative bacteria ^69^ ( Table 1; Figure 2, Underlying data ^44, 45^). CC4533 is sensitive to neomycin ( *Underlying data* ^44, 45^; Table 1; Figure 2). The diversity and antibiotic resistance patterns of Sphingomonadaceae isolates from drinking water show that the highest antibiotic resistance prevalence values were in members of the genera *Sphingomonas* and *Sphingobium*, especially in tap water and in water from dental chairs ^70^. *Sphingobium* isolates in drinking water are 89.3-100% penicillin-resistant, 96.4% polymyxin B-resistant, 25% sulfonamide-resistant and 100% neomycin-sensitive ^70^.

Many *Sphingobium* strains capable of degrading PAHs and other aromatic compounds have been isolated ^17– 40^. *Sphingobium yanoikuyae* strain B1, previously known as *Sphingomonas yanoikuyae* strain B1 and *Beijerinckia sp.* strain B1, has caught attention due to its versatile capabilities to degrade various environmental pollutants, such as biphenyl, naphthalene, phenanthrene, toluene, m-, p-xylene, anthracene ^17– 22^. *Sphingobium* sp. 22B isolated from soil contaminated with PAHs from Argentina has great capacity of degrading PAHs as unique sources of carbon in mineral medium and in phenanthrene microcosms assays ^23, 24^. Biphenyls and polychlorinated biphenyls (PCBs) are difficult to be completely mineralized by environmental microbes because of the accumulation of dead-end intermediates like benzoate and chlorobenzoates during biphenyl and PCB biodegradation. *Sphingobium fuliginis* HC3 strain isolated from PCBs-contaminated soil can degrade biphenyl and PCBs without dead-end intermediates accumulation ^25^.

*Sphingobium aromaticiconvertens* sp. nov. isolated from polluted river sediment can mineralize monochlorinated dibenzofurans ^26^. *Sphingobium xenophagum* Bayram can utilize branched 4-nonylphenol as a sole carbon source ^27^. *Sphingobium yanoikuyae* strain FM-2 isolated from river water is capable of degrading Bisphenol F ^28^. Three strains of *Sphingobium sp.* strain YBL1, strain YBL2 and strain YBL3 isolated from China were found to mineralize commonly used N, N-dimethyl-substituted phenylurea herbicides ^29, 30^. *Sphingobium wenxiniae* JZ-1 has a gene cluster that codes for phenoxybenzoic acid 1′,2′-dioxygenase, which allows this species to metabolize 3-phenoxybenzoic acid ^31^. *Sphingobium scionense sp*. nov is an aromatic hydrocarbon-degrading bacterium isolated from a PAH-contaminated soil in New Zealand ^32^. *Sphingobium yanoikuyae* XLDN2-5 isolated from petroleum-contaminated soils can degrade carbazole efficiently ^33– 35^. This strain could also co-metabolically catabolize dibenzofuran, dibenzothiophene, and benzothiophene ^33, 34, 36^. *Sphingobium yanoikuyae* SHJ is a strain isolated from shallow aquifer sediment in China that can degrade diethyl phthalate, which is used as a plasticizer ^37^.

Synthesis and accumulation of PHB is widespread in bacteria and depends on either the type of strain or the carbon source used in the metabolism ^38, 39^. PHB is important for the physiology of the PAH-degrading strain *Sphingobium sp*. 22B, isolated from semi-arid region of Patagonia (South America) as it accumulates significant amounts of this polyhydroxyalkanoate ^40^. PHB has been shown to play diverse roles under different degrees of water stress: it can serve as an endogenous carbon source under low water stress and can protect cells under high water stress ^40^.

We have shown in this work that CC4533 ( *Sphingobium yanoikuyae* strain PR86 variant) can utilize PAH, chlorinated alkanes, petroleum derivatives in 10W30 car motor oil, PHB and benzoic acids and phenyl acetate as sole carbon source in TP medium ( Figure 14– Figure 17). Recently we isolated and characterized a novel bacterial strain, LMJ/Bacterium strain clone LIB091_C05_1243 variant 16S ribosomal RNA gene, partial sequence (Accession number: MN633292.1) ^41^. The nearest relative of LMJ with a genus name is *Acidovorax sp.* strain A16OP12 (Accession #: MN519578.1) ^41^. LMJ can grow on TP-medium containing saturated hydrocarbons, PHB and PAH like CC4533 ^41^. LMJ hardly grows on TP medium containing phenyl acetate and benzoate unlike CC4533 ^41^. CC4533 strain grows more robustly than LMJ on every tested toxic aromatic compound and hydrocarbon containing plates at all doses ^41^. Hence, CC4533 displays a higher potential for environmental bioremediation than the bacterial strain LMJ strain ^41^.

In future we plan to optimize the process of uniformly coating the TP medium surface with chemicals as we have noticed that the tested hydrophobic chemicals were unevenly deposited after coating, when the solvent chloroform evaporates. We think that this uneven coating can affect proper utilization of these aromatic chemicals by CC4533. Our TP medium is a nutritionally stringent minimal medium compared to the traditional M9 minimal medium used for bacterial growth ^41^. The final concentrations of phosphate, nitrogen, magnesium and carbon in the M9 medium is approximately, 70-fold, 2.5-fold, 4-fold and 5-fold higher than that present in our lab’s TAP medium, respectively ^41^. We would like to test if CC4533 can grow on the M9 medium without an added carbon source like a sugar or acetic acid. If CC4533 fails to grow on the M9 medium plates without a carbon source, we will compare the growth of CC4533 in M9 medium containing different PAHs or other aromatic compounds as alternative carbon sources against that in TP medium. Additionally, we need to collaborate with a research lab that can test the concentration of these toxic organic chemicals in TP medium before and after the CC4533 growth, to get additional evidence that CC4533 is degrading PAH, PHB and other hydrocarbons in the TP medium.

Bacterial heterocyclic aromatic compound degradation pathways mainly involve the oxidation reactions such as angular dioxygenation of carbazole and dibenzofuran, lateral dioxygenation of dibenzothiophene in Kodama pathway, and S-oxidation of dibenzothiophene in 4S pathway ^34, 71, 72^. A consequence of the oxidation reaction is the formation of reactive oxygen species (ROS), which can damage DNA, proteins, and membranes ^73, 74^. Carotenoids present in a wide variety of bacteria, algae, fungi, and plants are the most prominent membrane-integrated antioxidants ^75^. *Sphingobium yanoikuyae* XLDN2-5, a yellow pigmented PAH-degrading strain, synthesizes zeaxanthin from β-carotene through β-cryptoxanthin via the carotenoid biosynthetic pathway ^49^. In *Sphingobium yanoikuyae* XLDN2-5, there is a direct correlation between the increase in the amount of zeaxanthin and the enhancement of hydrogen peroxide production during the biodegradation of heterocyclic aromatic compounds ^49^. High levels of carotenoids in this *Sphingobium* strain were consistent with the enhanced transcription of the gene encoding phytoene desaturase, one of the key enzymes for carotenoid biosynthesis ^49^. 

Pigment analyses of CC4533 strongly indicate the presence of β-carotene in the CC4533 pigment extract ( Figure 11; Figure 12). We plan to confirm our results by conducting HPLC analyses of the CC4533 pigment extract and a commercial pure sample of β-carotene. Carotenoid production can be measured in CC4533 grown on LB medium that contain the ROS species like hydrogen peroxide or contains photo-sensitizers like Rose Bengal that generates the ROS, singlet oxygen, in the presence of light and oxygen ^76^. Bleaching herbicides, such as norflurazon, interferes with the carotenoid biosynthetic pathway ^77^. Norflurazon blocks the enzyme Phytoene desaturase (PDS), which converts phytoene (colorless carotene) to red-colored lycopene ^78^. ROS sensitivity of CC4533 can be tested in the presence and absence of the inhibitor of PDS. Carotenoids are used as regulators for membrane fluidity by *Staphylococcus xylosus* ^79^. For two *Staphylococcus xylosus* strains there was an increase in staphyloxanthin and other carotenoids when grown at 10°C but no carotenoids could be detected when grown at 30°C ^79^. CC4533 cannot grow at 37°C and appears lot less yellow-pigmented at 30°C compared to when it is grown at 22°C ( Figure 4). Pigment reduction is more pronounced in CC4533 on TAP medium than on LB ( Figure 4). Quantitative carotenoid assays at different temperatures can be performed to study the temperature effect on pigment production.

Partial 16S rRNA gene sequence of CC4533 shows two transitional nucleotide changes when compared to that of the *Sphingobium yanoikuyae* strain PR86 (GenBank Accession #: MN232173.1) and to that of *Sphingobium yanoikuyae* strain NRB095 (Accession number: MK543001.1). These two nucleotide substitutions are in the 11 bp invariable sub-region (788 bp -798 bp) within C4 region ( Figure 13C). These results show that conserved regions of the 16S rRNA are not truly “conserved”. Conserved regions of the 16S rRNA gene exhibit considerable variations that need to be considered when using this gene as a biomarker ^80^.

Sphingomonads have attracted the attention of microbiologists and biotechnologists due to their biodegradative and biosynthetic capabilities, and have been utilized for a wide range of biotechnological applications from bioremediation of contaminants to production of extracellular polymers ^28^. We have shown in this study that CC4533 is a *Sphingobium yanoikuyae* strain and has traits that can be exploited for bioremediation upon further research. We found in the NCBI database, 20 genome assemblies of *Sphingobium yanoikuyae* (Representative genome: *Sphingobium yanoikuyae* ATCC 51230; ID: 3110) and 70 genome assemblies of uncharacterized environmental isolates. Because of funding limitations, we could not sequence the whole genome of CC4533 at the time of this manuscript submission. But we will have funds in fall 2020 to sequence the whole genome of CC4533 using the Pacific Biosciences technology. Whole genome sequencing will allow us to: **1**) confirm if CC4533 is a novel species of *Sphingobium* or a new strain of *Sphingobium yanoikuyae* and, **2**) will reveal genes in CC4533 that are recruited for degradation of xenobiotics and contribute to its metabolic diversity, relevant to environmental bioremediation and industrial biotechnology.

## Data availability

### Underlying data

Sphingobium yanoikuyae strain PR86 16S ribosomal RNA gene, partial sequence. Accession number, MN633285.1: https://www.ncbi.nlm.nih.gov/nuccore/MN232173.1

Figshare: Antibiotic sensitivity data of the bacterial strain CC4533 ( *Sphingobium yanoikuyae* strain PR86 variant; GenBank Accession number: MN633285.1) and *Chlamydomonas reinhardtii* strain 4A+. https://doi.org/10.6084/m9.figshare.12465863.v1 ^44^

This project contains the following underlying data:

- CC4533 Data S1 (XLSX). Means of the zones of inhibitions of the bacterial strain CC4533 ( *Sphingobium yanoikuyae* strain PR86 variant) and *Chlamydomonas* and corresponding standard deviations.
- CC4533 Data S2 (XLSX). Statistical analyses of the zones of inhibitions of the bacterial strain CC4533 ( *Sphingobium yanoikuyae* strain PR86 variant) and *Chlamydomonas*, induced by four antibiotics.

Figshare: Images of antibiotic plates of the bacterial strain CC4533 ( *Sphingobium yanoikuyae* PR86 strain variant partial 16S rRNA sequence; GenBank Accession # MN633285.1) and green micro-alga *Chlamydomonas* from the antibiotic susceptibility disc diffusion tests. https://doi.org/10.6084/m9.figshare.12465953.v1 ^45^. This project contains 16 images of antibiotic plates used for the antibiotic susceptibility tests using the disc diffusion method for *Chlamydomonas* and the bacterial strain, CC4533 ( *Sphingobium yanoikuyae* PR86 strain variant).

Figshare: Growth of the bacterial strain CC4533 (Sphingobium yanoikuyae PR86 strain variant partial 16S rRNA sequence; GenBank Accession # MN633285.1) and Staphylococcus aureus on Tryptic Soy agar medium containing 5% sheep blood. https://doi.org/10.6084/m9.figshare.12478544.v1 ^46^. This project contains six images that show the growth of the bacterial strain CC4533 ( *Sphingobium yanoikuyae* PR86 strain variant) and *Staphylococcus aureus* on Tryptic Soy agar medium plates containing 5% sheep blood over a period of 3 days at 30°C.

Figshare: Tests using Tris-Phosphate medium (TP) to see if hydrocarbons, aromatic compounds and polyhydroxyalkanoates can be used by the bacterium CC4533 ( *Sphingobium yanoikuyae* PR86 strain variant, partial 16S rRNA sequence; GenBank Accession # MN633285.1) as the sole carbon source. https://doi.org/10.6084/m9.figshare.12465989.v1 ^48^. This project contains 21 images of TP (Tris-Phosphate) medium plates containing different alternative carbon sources. Bacterium CC4533 ( *Sphingobium yanoikuyae* PR86 strain variant) was streaked on these chemical plates to test if CC4533 can utilize these chemicals as the sole carbon source for energy and growth.

Figshare: 16S rRNA partial gene sequences of the bacterial strain CC4533 (Sphingobium yanoikuyae PR86 strain variant partial 16S rRNA sequence; GenBank Accession # MN633285.1). https://doi.org/10.6084/m9.figshare.12466043.v1 ^52^.

This project contains the following underlying data:

- Abi extension files obtained from partial sequencing of the 16S rRNA gene of CC4533 strain ( *Sphingobium yanoikuyae* PR86 strain variant).
- Corresponding text files of DNA sequences

Data are available under the terms of the Creative Commons Attribution 4.0 International license (CC-BY 4.0).
