# Supplementary figures and images for: Isolation and characterization of a novel Sphingobium yanoikuyae strain variant that uses biohazardous saturated hydrocarbons and aromatic compounds as sole carbon sources
Source: F1000Res. 2020 Jul 24;9:767. [Version 1] doi: 10.12688/f1000research.25284.1 (PMC7477647; doi:10.12688/f1000research.25284.1)

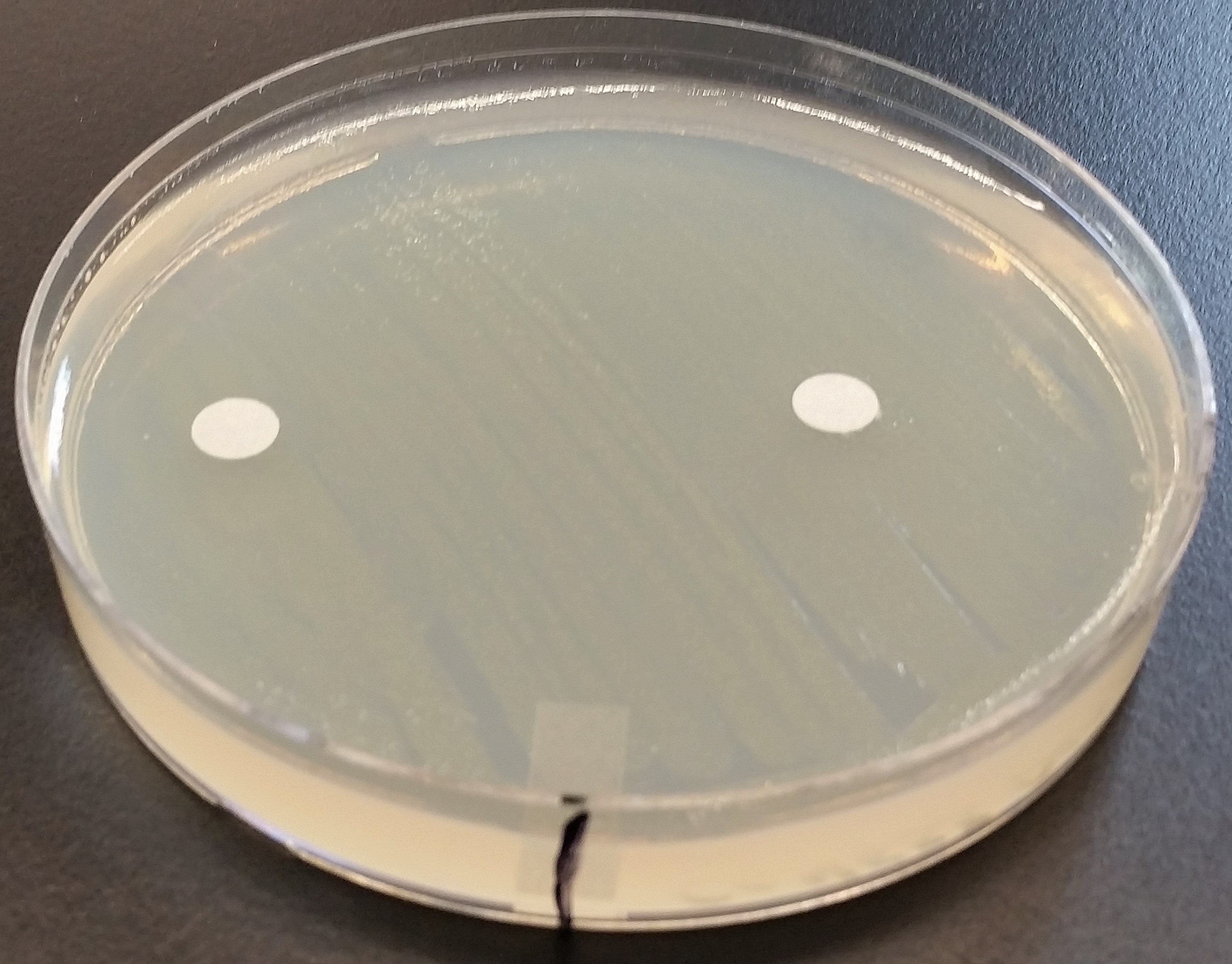

Supplement: Images of antibiotic plates of the bacterial strain CC4533 (Sphingobium yanoikuyae PR86 strain variant partial 16S rRNA sequence; GenBank Accession # MN633285.1) and green micro-alga Chlamydomonas from the antibiotic susceptibility disc diffusion tests. — The file contains 16 images of antibiotic plates used for the antibiotic susceptibility tests using the disc diffusion method for Chlamydomonas and the bacterial strain, CC4533 (Sphingobium yanoikuyae PR86 strain variant). Antibiotics tested are: penicillin, chloramphenicol, polymyxin B and neomycin. Two different doses of antibiotics were used: 50 and 100 micrograms of each antibiotics. On the antibiotic plates, the filter paper disc on the left contains the antibiotic and that on the right contains sterile water (control). CC4533 (Sphingobium yanoikuyae PR86 strain variant) plates were imaged after 3 days of growth and Chlamydomonas plates were imaged after 4 days of growth at room temperature (22C). [file f1000research-9-27904-s0001.tgz › CC453350microPenicillin.jpg]

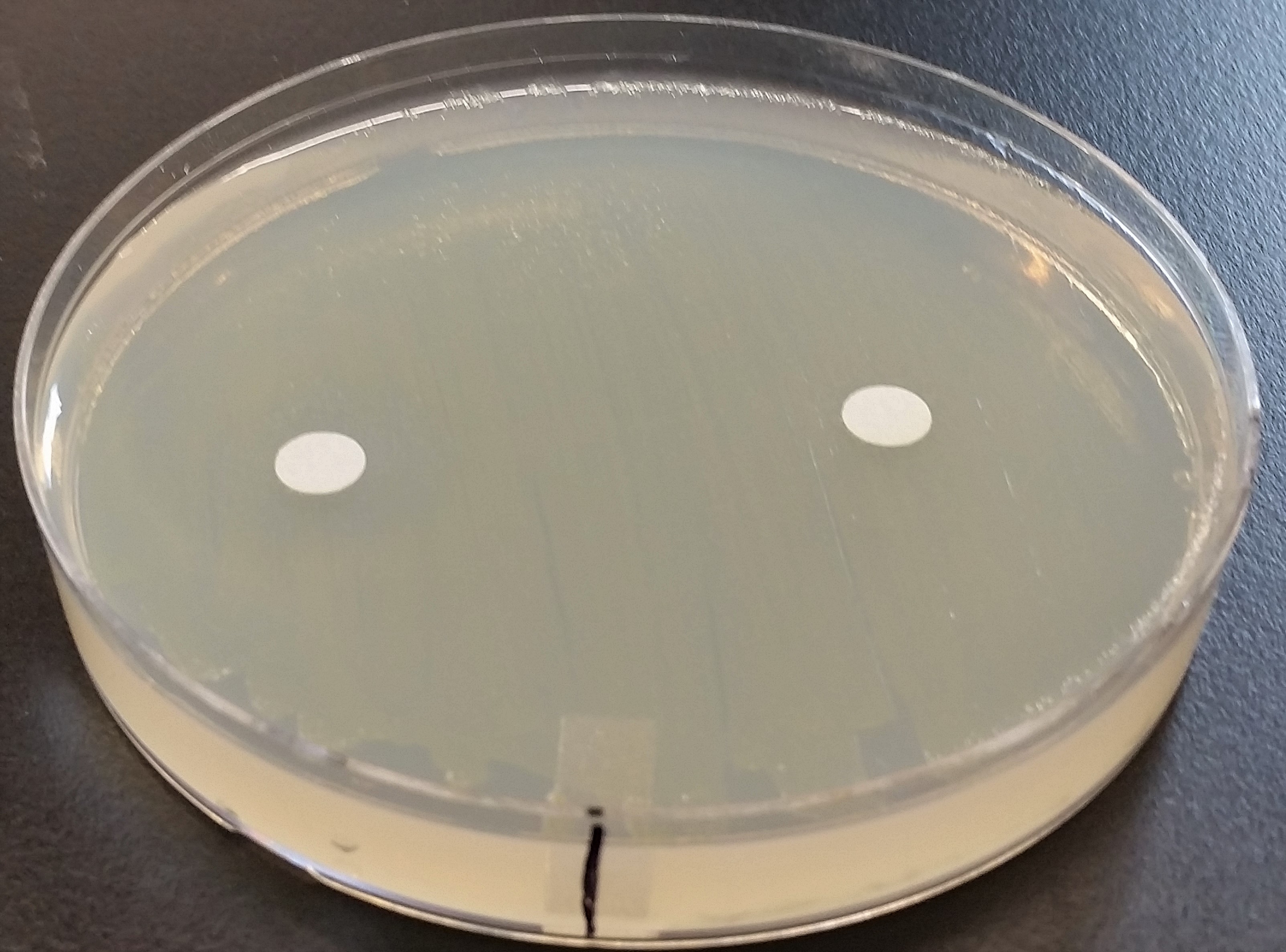

Supplement: Images of antibiotic plates of the bacterial strain CC4533 (Sphingobium yanoikuyae PR86 strain variant partial 16S rRNA sequence; GenBank Accession # MN633285.1) and green micro-alga Chlamydomonas from the antibiotic susceptibility disc diffusion tests. — The file contains 16 images of antibiotic plates used for the antibiotic susceptibility tests using the disc diffusion method for Chlamydomonas and the bacterial strain, CC4533 (Sphingobium yanoikuyae PR86 strain variant). Antibiotics tested are: penicillin, chloramphenicol, polymyxin B and neomycin. Two different doses of antibiotics were used: 50 and 100 micrograms of each antibiotics. On the antibiotic plates, the filter paper disc on the left contains the antibiotic and that on the right contains sterile water (control). CC4533 (Sphingobium yanoikuyae PR86 strain variant) plates were imaged after 3 days of growth and Chlamydomonas plates were imaged after 4 days of growth at room temperature (22C). [file f1000research-9-27904-s0001.tgz › CC4533100microPenicillin.jpg]

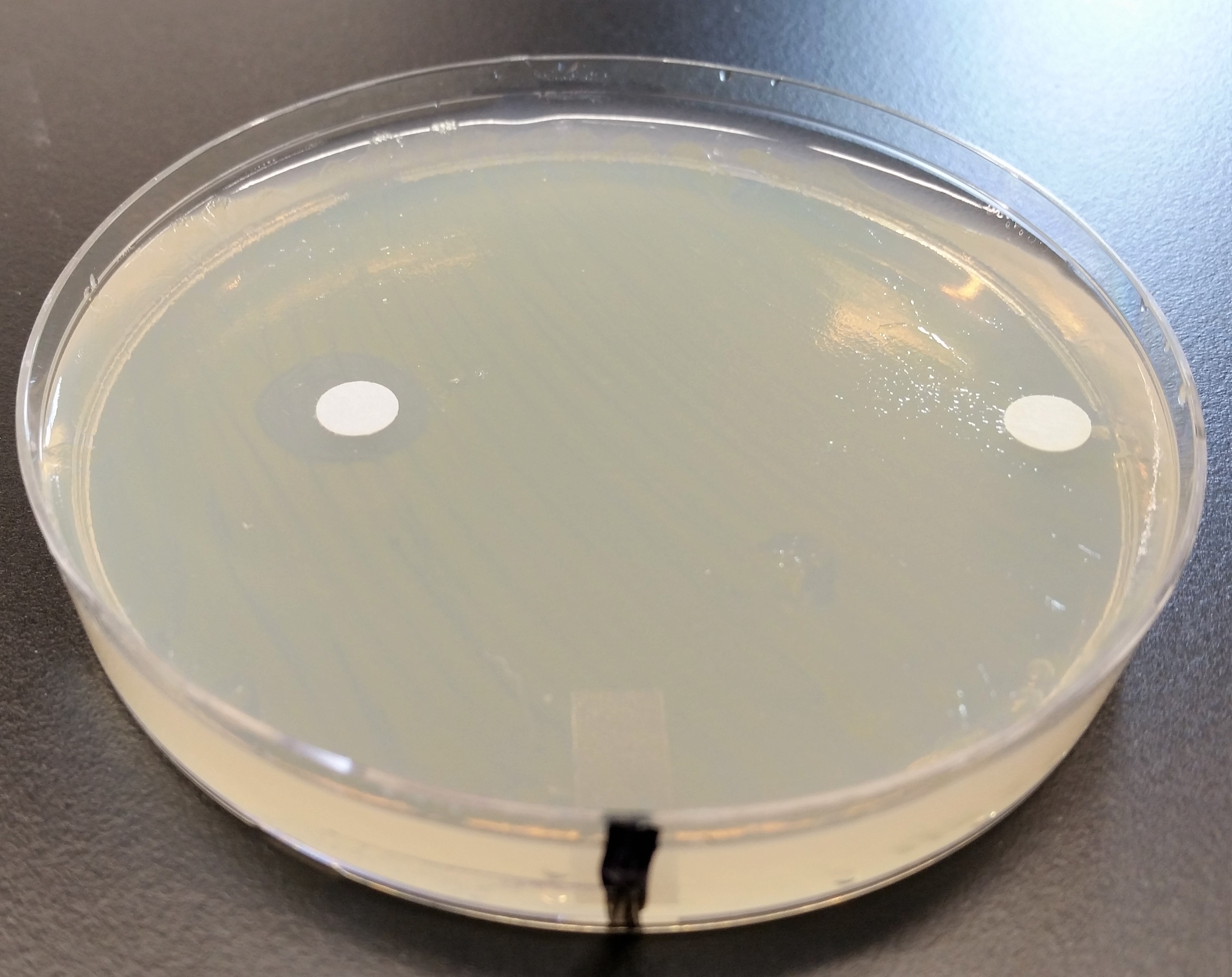

Supplement: Images of antibiotic plates of the bacterial strain CC4533 (Sphingobium yanoikuyae PR86 strain variant partial 16S rRNA sequence; GenBank Accession # MN633285.1) and green micro-alga Chlamydomonas from the antibiotic susceptibility disc diffusion tests. — The file contains 16 images of antibiotic plates used for the antibiotic susceptibility tests using the disc diffusion method for Chlamydomonas and the bacterial strain, CC4533 (Sphingobium yanoikuyae PR86 strain variant). Antibiotics tested are: penicillin, chloramphenicol, polymyxin B and neomycin. Two different doses of antibiotics were used: 50 and 100 micrograms of each antibiotics. On the antibiotic plates, the filter paper disc on the left contains the antibiotic and that on the right contains sterile water (control). CC4533 (Sphingobium yanoikuyae PR86 strain variant) plates were imaged after 3 days of growth and Chlamydomonas plates were imaged after 4 days of growth at room temperature (22C). [file f1000research-9-27904-s0001.tgz › CC453350microNeomycin.jpg]

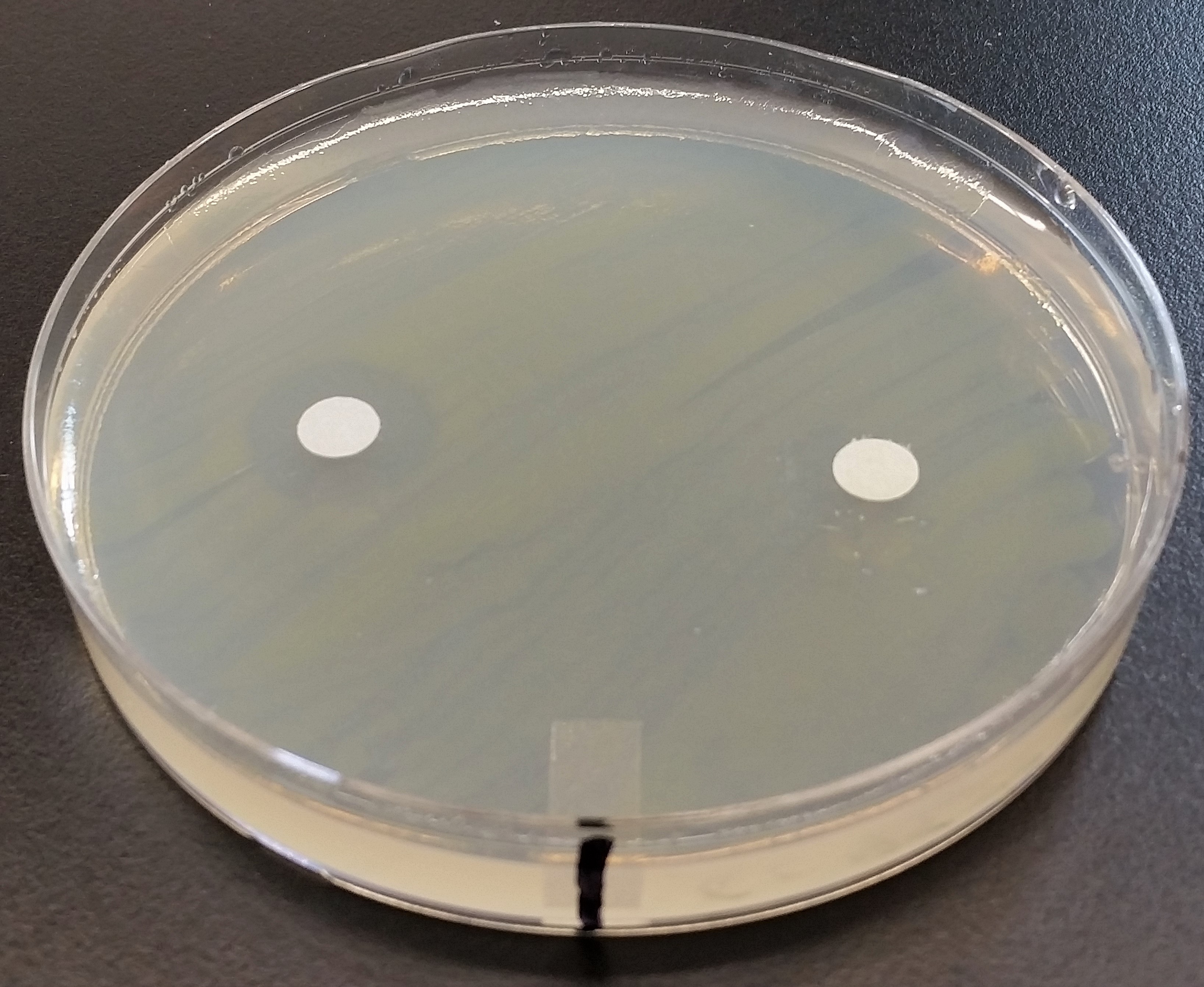

Supplement: Images of antibiotic plates of the bacterial strain CC4533 (Sphingobium yanoikuyae PR86 strain variant partial 16S rRNA sequence; GenBank Accession # MN633285.1) and green micro-alga Chlamydomonas from the antibiotic susceptibility disc diffusion tests. — The file contains 16 images of antibiotic plates used for the antibiotic susceptibility tests using the disc diffusion method for Chlamydomonas and the bacterial strain, CC4533 (Sphingobium yanoikuyae PR86 strain variant). Antibiotics tested are: penicillin, chloramphenicol, polymyxin B and neomycin. Two different doses of antibiotics were used: 50 and 100 micrograms of each antibiotics. On the antibiotic plates, the filter paper disc on the left contains the antibiotic and that on the right contains sterile water (control). CC4533 (Sphingobium yanoikuyae PR86 strain variant) plates were imaged after 3 days of growth and Chlamydomonas plates were imaged after 4 days of growth at room temperature (22C). [file f1000research-9-27904-s0001.tgz › CC4533100microNeomycin.jpg]

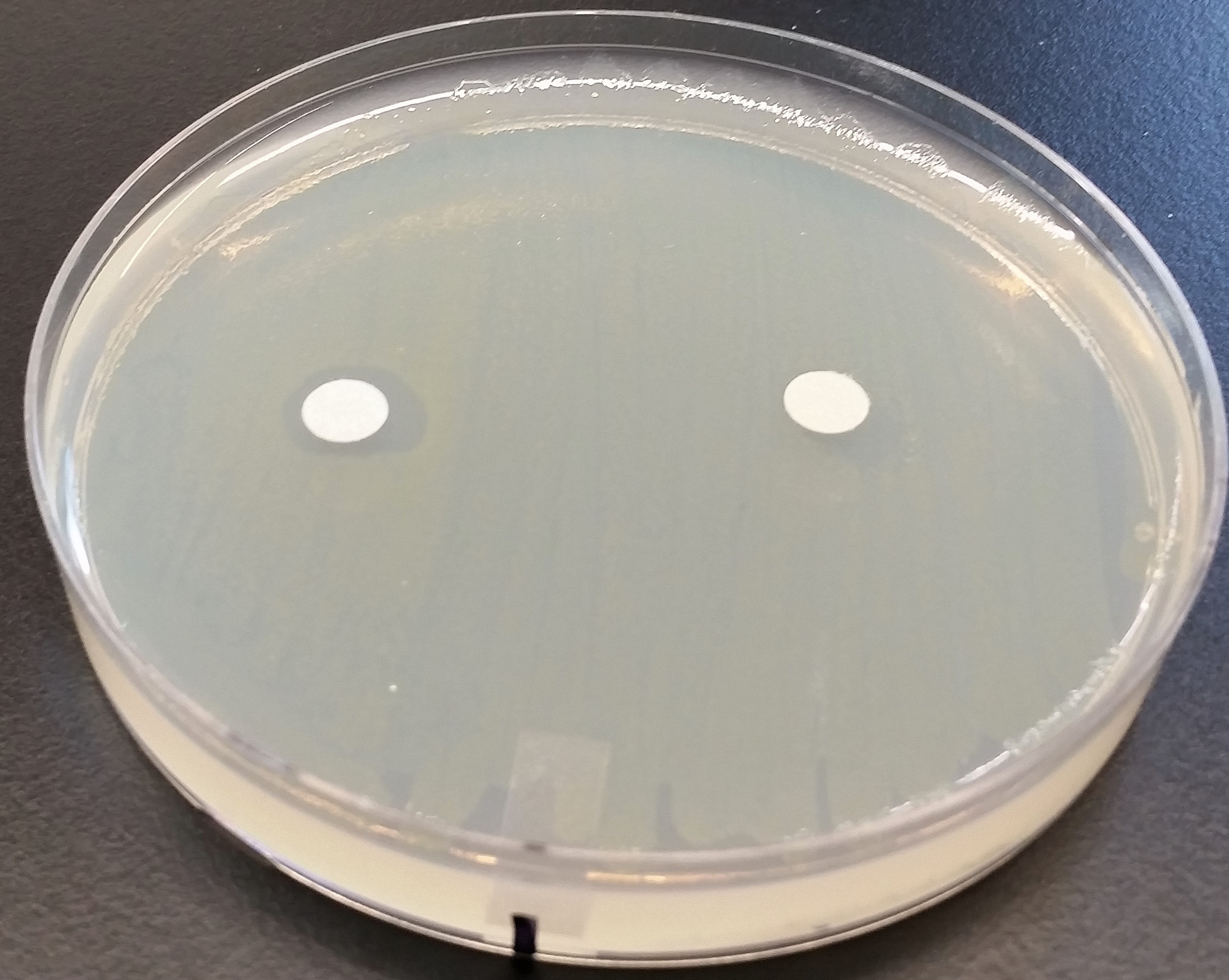

Supplement: Images of antibiotic plates of the bacterial strain CC4533 (Sphingobium yanoikuyae PR86 strain variant partial 16S rRNA sequence; GenBank Accession # MN633285.1) and green micro-alga Chlamydomonas from the antibiotic susceptibility disc diffusion tests. — The file contains 16 images of antibiotic plates used for the antibiotic susceptibility tests using the disc diffusion method for Chlamydomonas and the bacterial strain, CC4533 (Sphingobium yanoikuyae PR86 strain variant). Antibiotics tested are: penicillin, chloramphenicol, polymyxin B and neomycin. Two different doses of antibiotics were used: 50 and 100 micrograms of each antibiotics. On the antibiotic plates, the filter paper disc on the left contains the antibiotic and that on the right contains sterile water (control). CC4533 (Sphingobium yanoikuyae PR86 strain variant) plates were imaged after 3 days of growth and Chlamydomonas plates were imaged after 4 days of growth at room temperature (22C). [file f1000research-9-27904-s0001.tgz › CC453350microPolymyxinB.jpg]

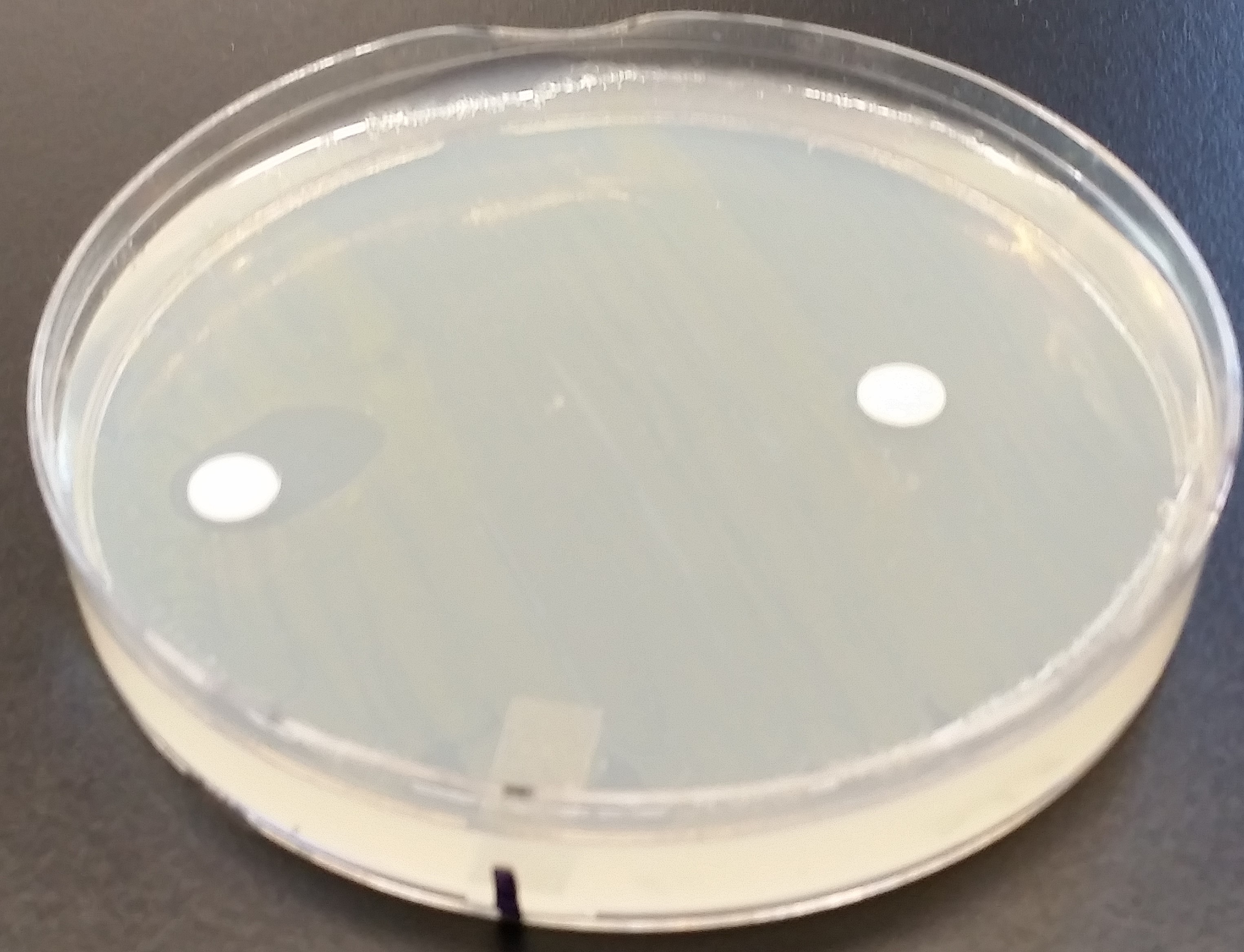

Supplement: Images of antibiotic plates of the bacterial strain CC4533 (Sphingobium yanoikuyae PR86 strain variant partial 16S rRNA sequence; GenBank Accession # MN633285.1) and green micro-alga Chlamydomonas from the antibiotic susceptibility disc diffusion tests. — The file contains 16 images of antibiotic plates used for the antibiotic susceptibility tests using the disc diffusion method for Chlamydomonas and the bacterial strain, CC4533 (Sphingobium yanoikuyae PR86 strain variant). Antibiotics tested are: penicillin, chloramphenicol, polymyxin B and neomycin. Two different doses of antibiotics were used: 50 and 100 micrograms of each antibiotics. On the antibiotic plates, the filter paper disc on the left contains the antibiotic and that on the right contains sterile water (control). CC4533 (Sphingobium yanoikuyae PR86 strain variant) plates were imaged after 3 days of growth and Chlamydomonas plates were imaged after 4 days of growth at room temperature (22C). [file f1000research-9-27904-s0001.tgz › CC4533100micropolymyxinB.jpg]

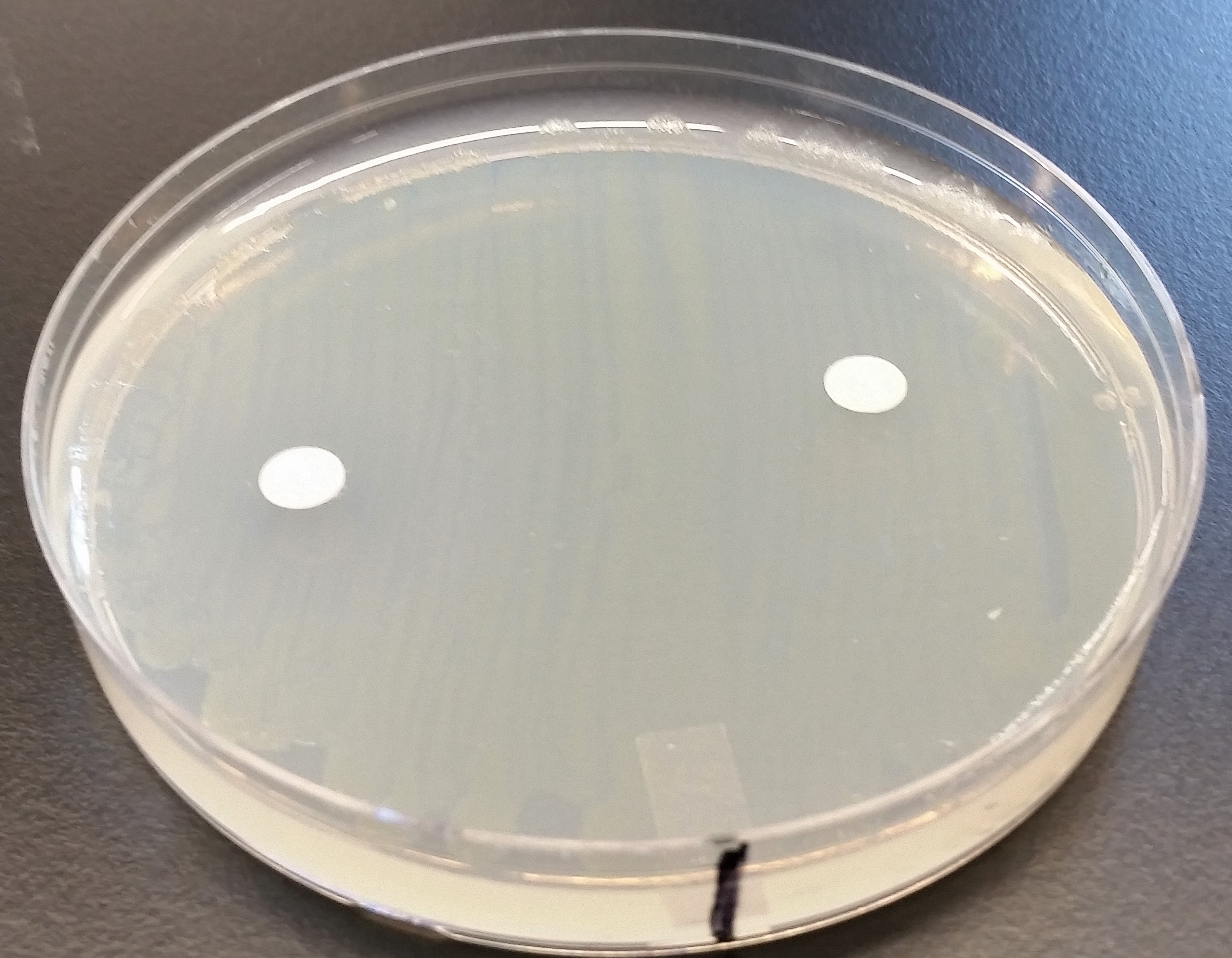

Supplement: Images of antibiotic plates of the bacterial strain CC4533 (Sphingobium yanoikuyae PR86 strain variant partial 16S rRNA sequence; GenBank Accession # MN633285.1) and green micro-alga Chlamydomonas from the antibiotic susceptibility disc diffusion tests. — The file contains 16 images of antibiotic plates used for the antibiotic susceptibility tests using the disc diffusion method for Chlamydomonas and the bacterial strain, CC4533 (Sphingobium yanoikuyae PR86 strain variant). Antibiotics tested are: penicillin, chloramphenicol, polymyxin B and neomycin. Two different doses of antibiotics were used: 50 and 100 micrograms of each antibiotics. On the antibiotic plates, the filter paper disc on the left contains the antibiotic and that on the right contains sterile water (control). CC4533 (Sphingobium yanoikuyae PR86 strain variant) plates were imaged after 3 days of growth and Chlamydomonas plates were imaged after 4 days of growth at room temperature (22C). [file f1000research-9-27904-s0001.tgz › CC453350microChloramphenicol.jpg]

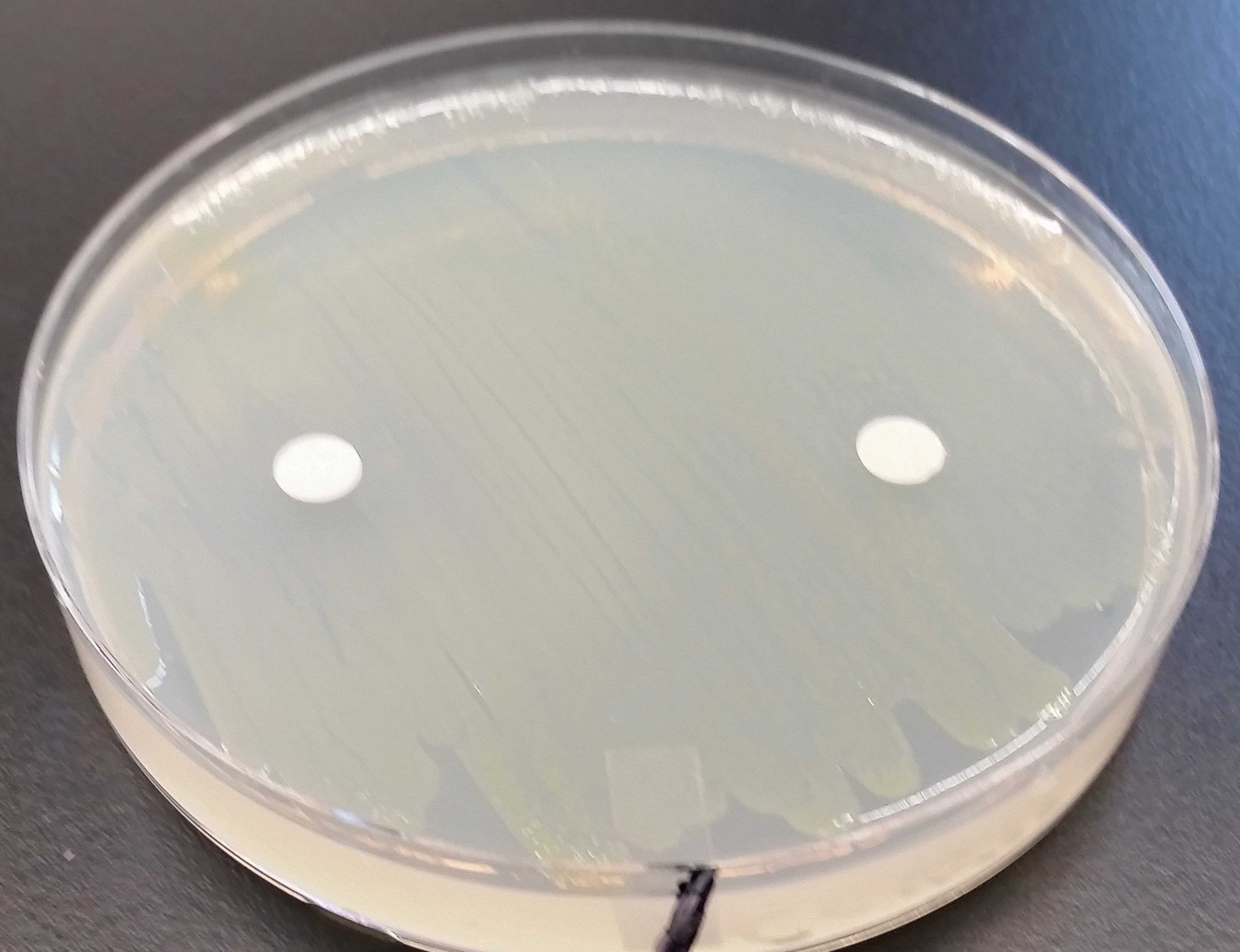

Supplement: Images of antibiotic plates of the bacterial strain CC4533 (Sphingobium yanoikuyae PR86 strain variant partial 16S rRNA sequence; GenBank Accession # MN633285.1) and green micro-alga Chlamydomonas from the antibiotic susceptibility disc diffusion tests. — The file contains 16 images of antibiotic plates used for the antibiotic susceptibility tests using the disc diffusion method for Chlamydomonas and the bacterial strain, CC4533 (Sphingobium yanoikuyae PR86 strain variant). Antibiotics tested are: penicillin, chloramphenicol, polymyxin B and neomycin. Two different doses of antibiotics were used: 50 and 100 micrograms of each antibiotics. On the antibiotic plates, the filter paper disc on the left contains the antibiotic and that on the right contains sterile water (control). CC4533 (Sphingobium yanoikuyae PR86 strain variant) plates were imaged after 3 days of growth and Chlamydomonas plates were imaged after 4 days of growth at room temperature (22C). [file f1000research-9-27904-s0001.tgz › CC4533100microChloramphenicol.jpg]

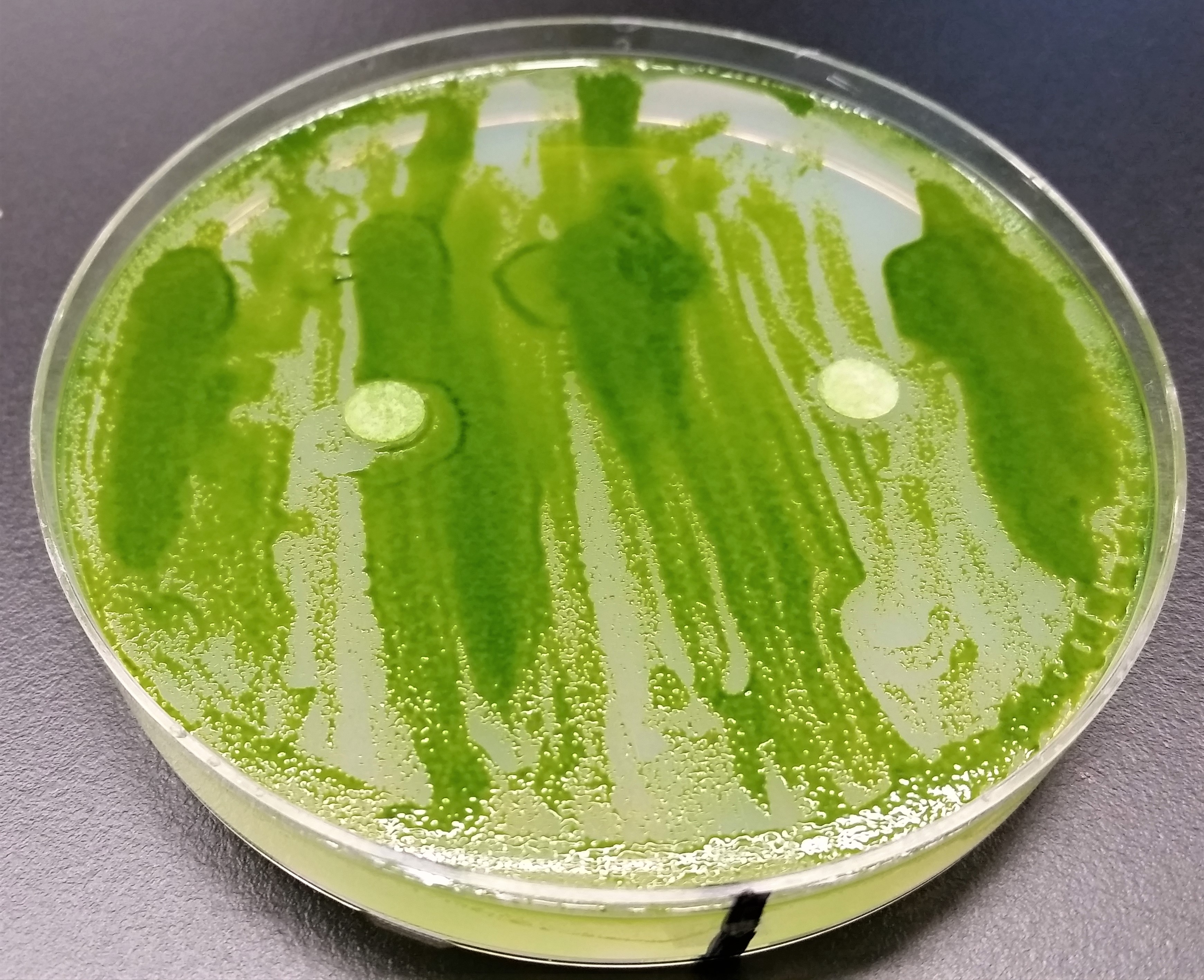

Supplement: Images of antibiotic plates of the bacterial strain CC4533 (Sphingobium yanoikuyae PR86 strain variant partial 16S rRNA sequence; GenBank Accession # MN633285.1) and green micro-alga Chlamydomonas from the antibiotic susceptibility disc diffusion tests. — The file contains 16 images of antibiotic plates used for the antibiotic susceptibility tests using the disc diffusion method for Chlamydomonas and the bacterial strain, CC4533 (Sphingobium yanoikuyae PR86 strain variant). Antibiotics tested are: penicillin, chloramphenicol, polymyxin B and neomycin. Two different doses of antibiotics were used: 50 and 100 micrograms of each antibiotics. On the antibiotic plates, the filter paper disc on the left contains the antibiotic and that on the right contains sterile water (control). CC4533 (Sphingobium yanoikuyae PR86 strain variant) plates were imaged after 3 days of growth and Chlamydomonas plates were imaged after 4 days of growth at room temperature (22C). [file f1000research-9-27904-s0001.tgz › 50microPenicillinChlamycropped.jpg]

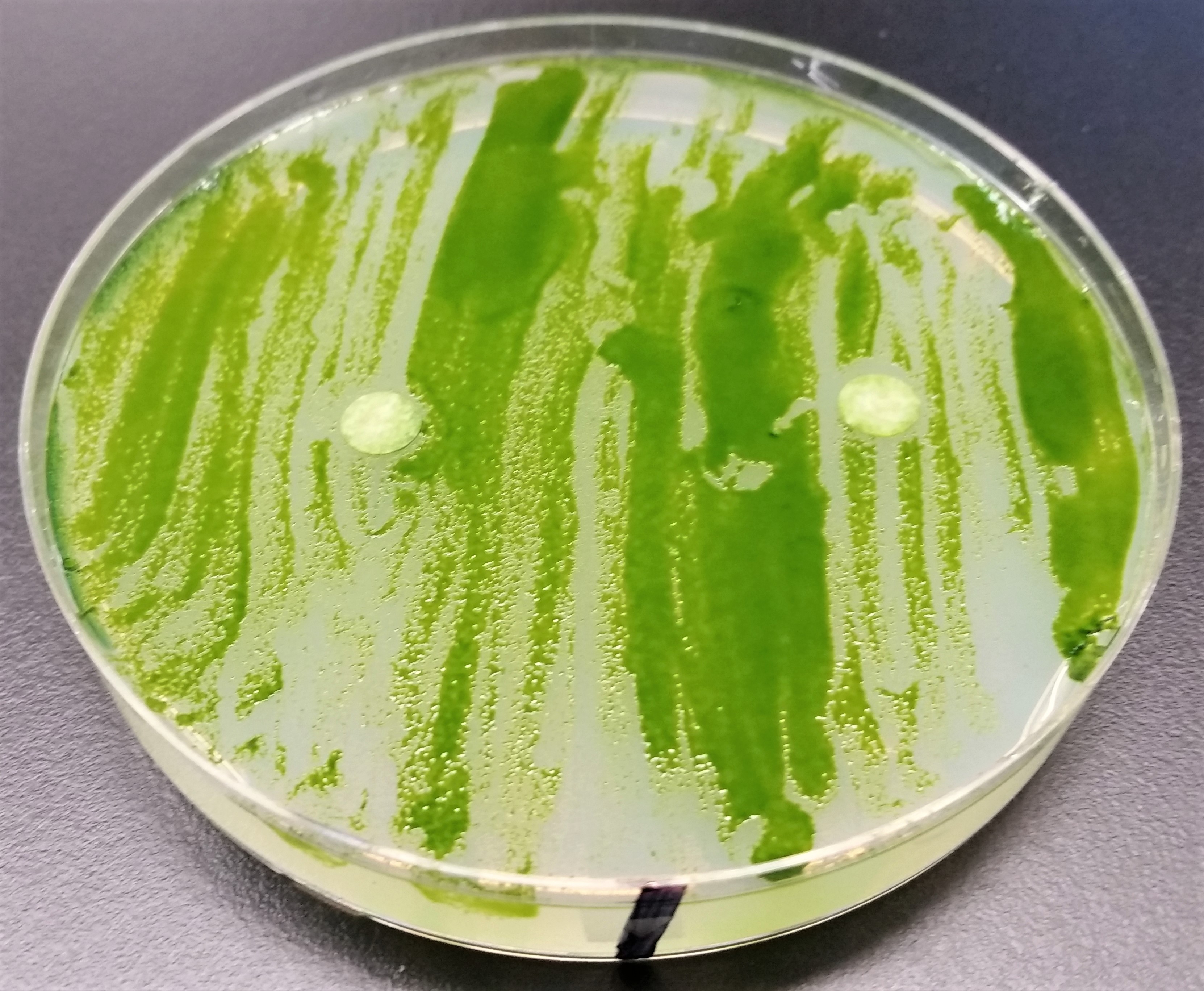

Supplement: Images of antibiotic plates of the bacterial strain CC4533 (Sphingobium yanoikuyae PR86 strain variant partial 16S rRNA sequence; GenBank Accession # MN633285.1) and green micro-alga Chlamydomonas from the antibiotic susceptibility disc diffusion tests. — The file contains 16 images of antibiotic plates used for the antibiotic susceptibility tests using the disc diffusion method for Chlamydomonas and the bacterial strain, CC4533 (Sphingobium yanoikuyae PR86 strain variant). Antibiotics tested are: penicillin, chloramphenicol, polymyxin B and neomycin. Two different doses of antibiotics were used: 50 and 100 micrograms of each antibiotics. On the antibiotic plates, the filter paper disc on the left contains the antibiotic and that on the right contains sterile water (control). CC4533 (Sphingobium yanoikuyae PR86 strain variant) plates were imaged after 3 days of growth and Chlamydomonas plates were imaged after 4 days of growth at room temperature (22C). [file f1000research-9-27904-s0001.tgz › 100microPenicillincropped.jpg]

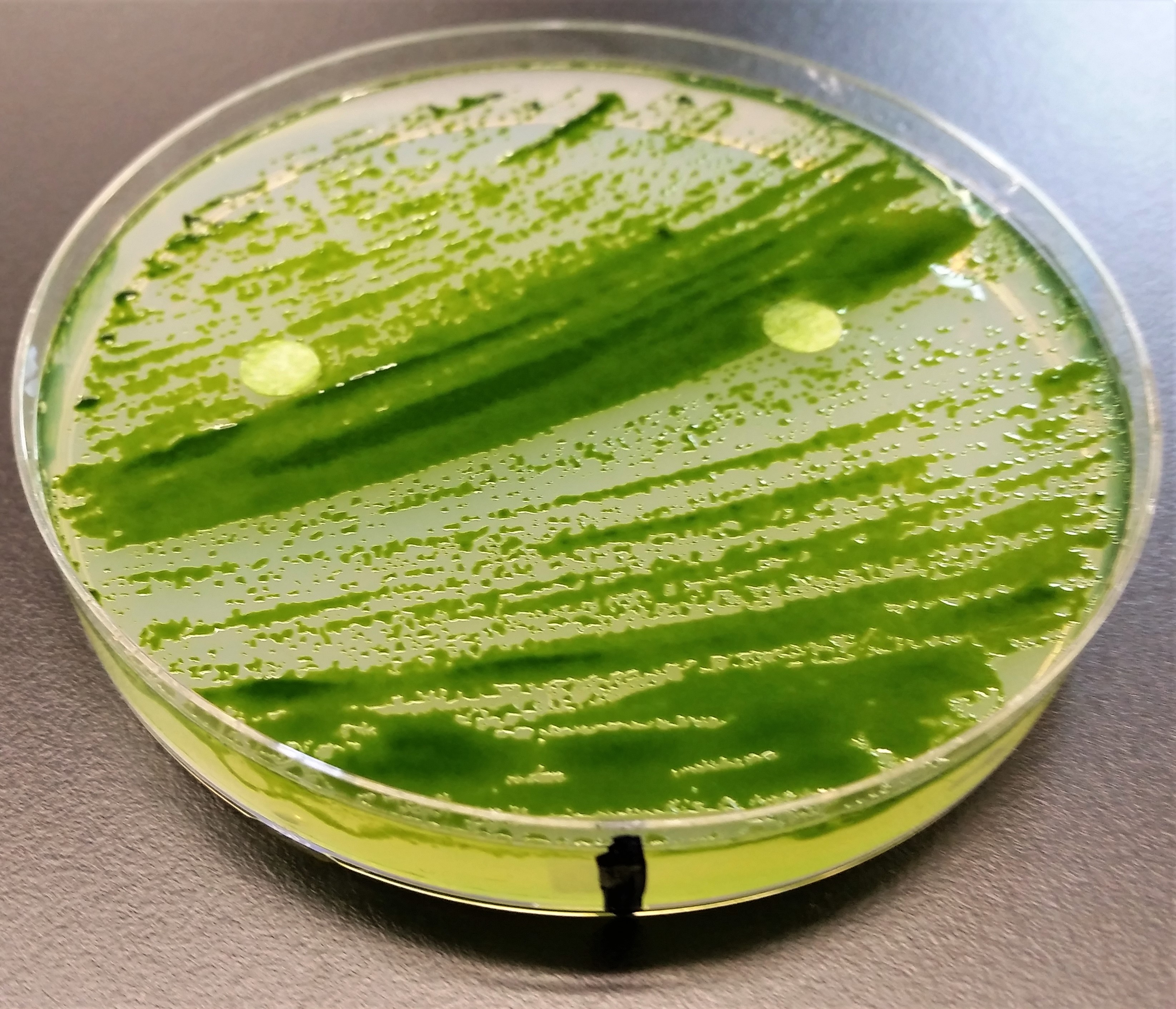

Supplement: Images of antibiotic plates of the bacterial strain CC4533 (Sphingobium yanoikuyae PR86 strain variant partial 16S rRNA sequence; GenBank Accession # MN633285.1) and green micro-alga Chlamydomonas from the antibiotic susceptibility disc diffusion tests. — The file contains 16 images of antibiotic plates used for the antibiotic susceptibility tests using the disc diffusion method for Chlamydomonas and the bacterial strain, CC4533 (Sphingobium yanoikuyae PR86 strain variant). Antibiotics tested are: penicillin, chloramphenicol, polymyxin B and neomycin. Two different doses of antibiotics were used: 50 and 100 micrograms of each antibiotics. On the antibiotic plates, the filter paper disc on the left contains the antibiotic and that on the right contains sterile water (control). CC4533 (Sphingobium yanoikuyae PR86 strain variant) plates were imaged after 3 days of growth and Chlamydomonas plates were imaged after 4 days of growth at room temperature (22C). [file f1000research-9-27904-s0001.tgz › 50microChloramphenicolChlamycropped.jpg]

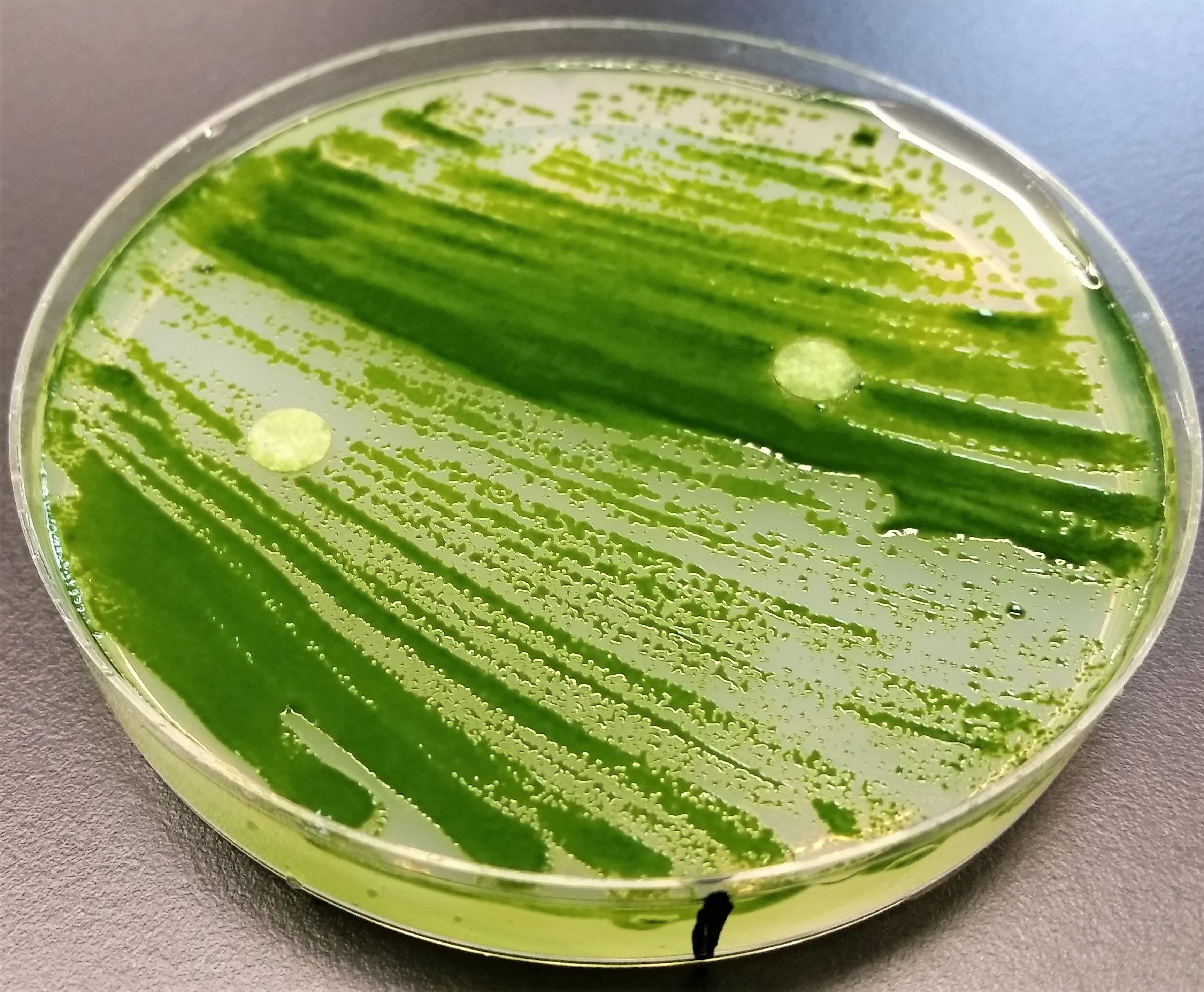

Supplement: Images of antibiotic plates of the bacterial strain CC4533 (Sphingobium yanoikuyae PR86 strain variant partial 16S rRNA sequence; GenBank Accession # MN633285.1) and green micro-alga Chlamydomonas from the antibiotic susceptibility disc diffusion tests. — The file contains 16 images of antibiotic plates used for the antibiotic susceptibility tests using the disc diffusion method for Chlamydomonas and the bacterial strain, CC4533 (Sphingobium yanoikuyae PR86 strain variant). Antibiotics tested are: penicillin, chloramphenicol, polymyxin B and neomycin. Two different doses of antibiotics were used: 50 and 100 micrograms of each antibiotics. On the antibiotic plates, the filter paper disc on the left contains the antibiotic and that on the right contains sterile water (control). CC4533 (Sphingobium yanoikuyae PR86 strain variant) plates were imaged after 3 days of growth and Chlamydomonas plates were imaged after 4 days of growth at room temperature (22C). [file f1000research-9-27904-s0001.tgz › 100microchloramphenicolcropped.jpg]

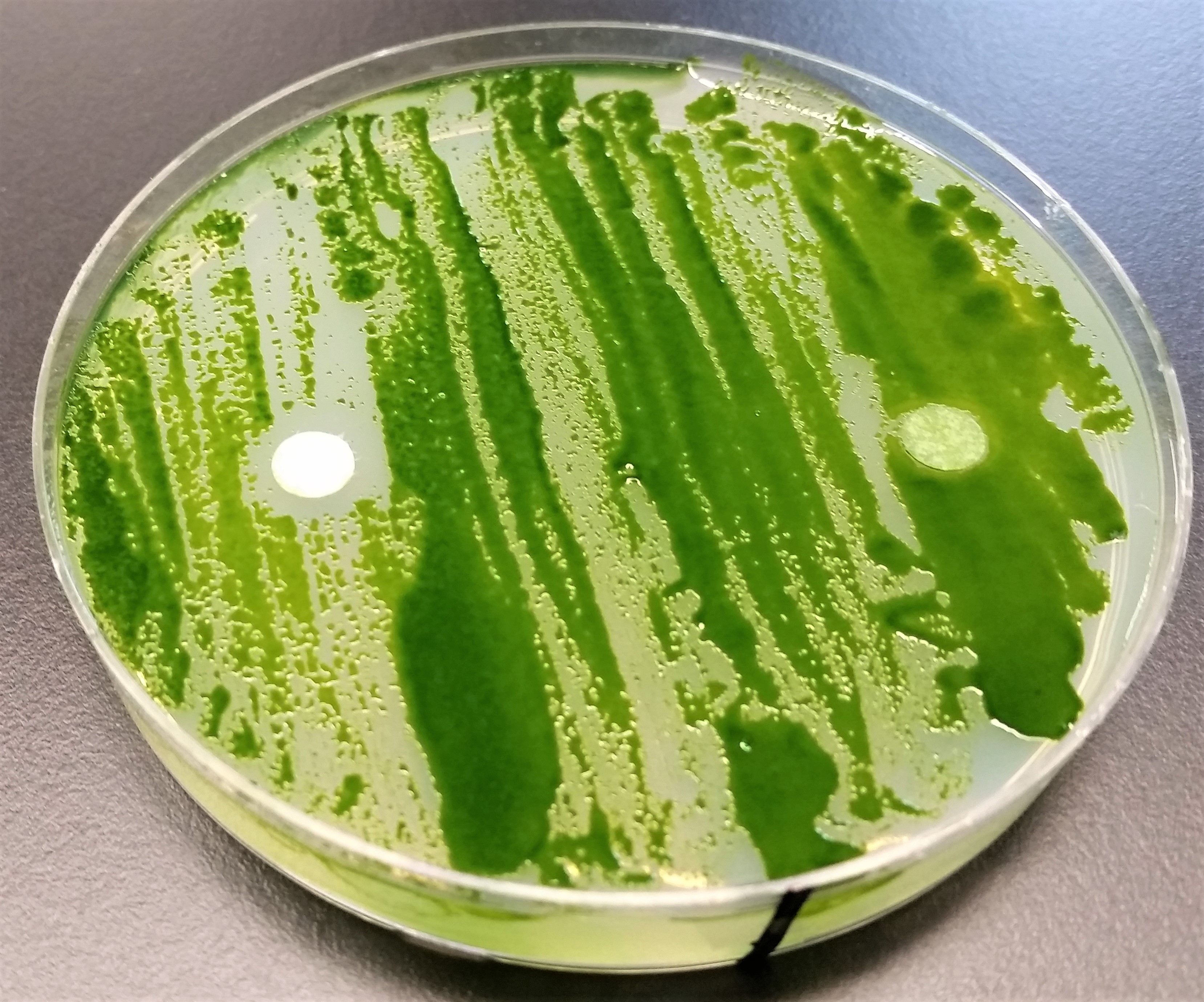

Supplement: Images of antibiotic plates of the bacterial strain CC4533 (Sphingobium yanoikuyae PR86 strain variant partial 16S rRNA sequence; GenBank Accession # MN633285.1) and green micro-alga Chlamydomonas from the antibiotic susceptibility disc diffusion tests. — The file contains 16 images of antibiotic plates used for the antibiotic susceptibility tests using the disc diffusion method for Chlamydomonas and the bacterial strain, CC4533 (Sphingobium yanoikuyae PR86 strain variant). Antibiotics tested are: penicillin, chloramphenicol, polymyxin B and neomycin. Two different doses of antibiotics were used: 50 and 100 micrograms of each antibiotics. On the antibiotic plates, the filter paper disc on the left contains the antibiotic and that on the right contains sterile water (control). CC4533 (Sphingobium yanoikuyae PR86 strain variant) plates were imaged after 3 days of growth and Chlamydomonas plates were imaged after 4 days of growth at room temperature (22C). [file f1000research-9-27904-s0001.tgz › 50microNeomycinChlamycropped.jpg]

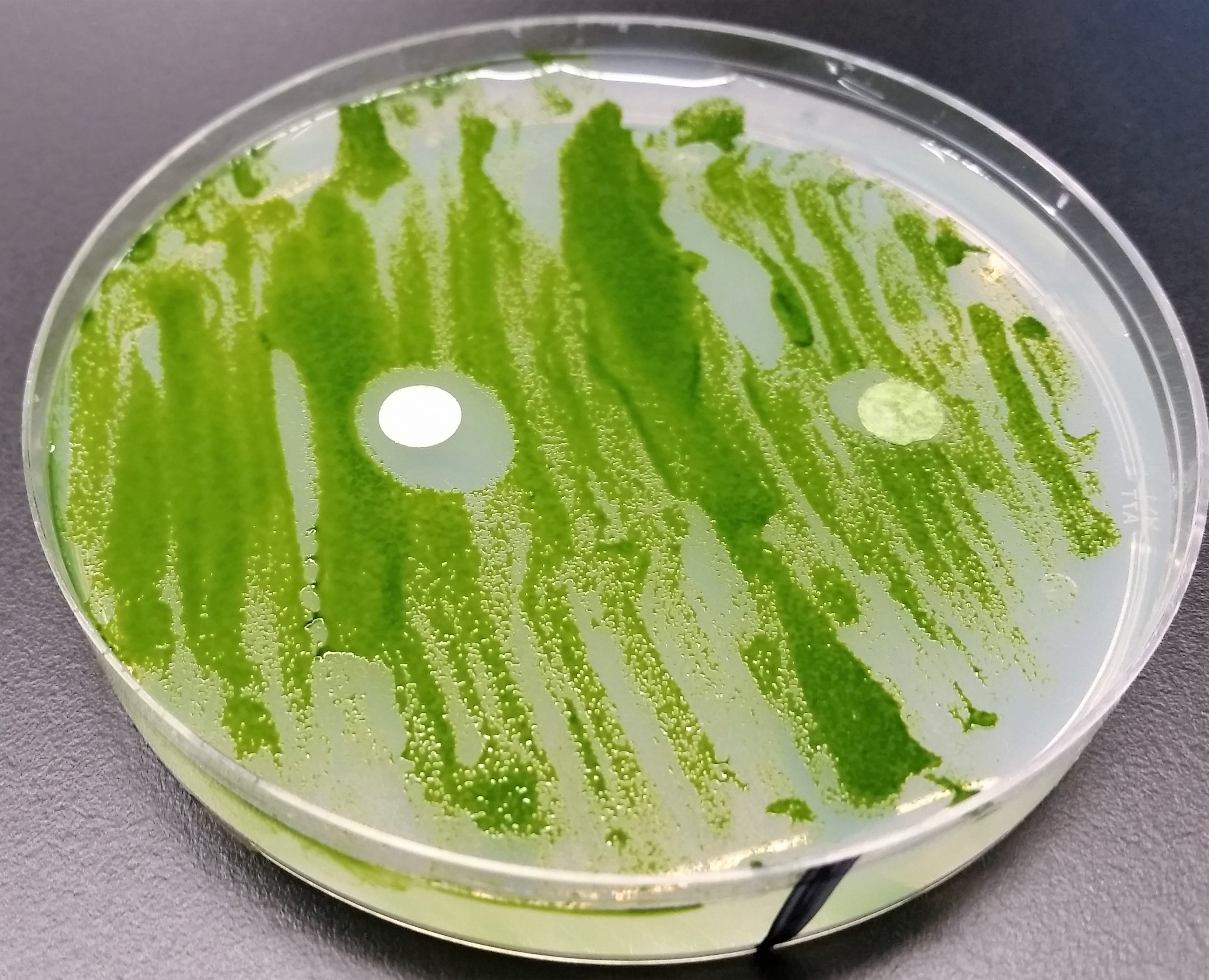

Supplement: Images of antibiotic plates of the bacterial strain CC4533 (Sphingobium yanoikuyae PR86 strain variant partial 16S rRNA sequence; GenBank Accession # MN633285.1) and green micro-alga Chlamydomonas from the antibiotic susceptibility disc diffusion tests. — The file contains 16 images of antibiotic plates used for the antibiotic susceptibility tests using the disc diffusion method for Chlamydomonas and the bacterial strain, CC4533 (Sphingobium yanoikuyae PR86 strain variant). Antibiotics tested are: penicillin, chloramphenicol, polymyxin B and neomycin. Two different doses of antibiotics were used: 50 and 100 micrograms of each antibiotics. On the antibiotic plates, the filter paper disc on the left contains the antibiotic and that on the right contains sterile water (control). CC4533 (Sphingobium yanoikuyae PR86 strain variant) plates were imaged after 3 days of growth and Chlamydomonas plates were imaged after 4 days of growth at room temperature (22C). [file f1000research-9-27904-s0001.tgz › 100microNeomycincropped.jpg]

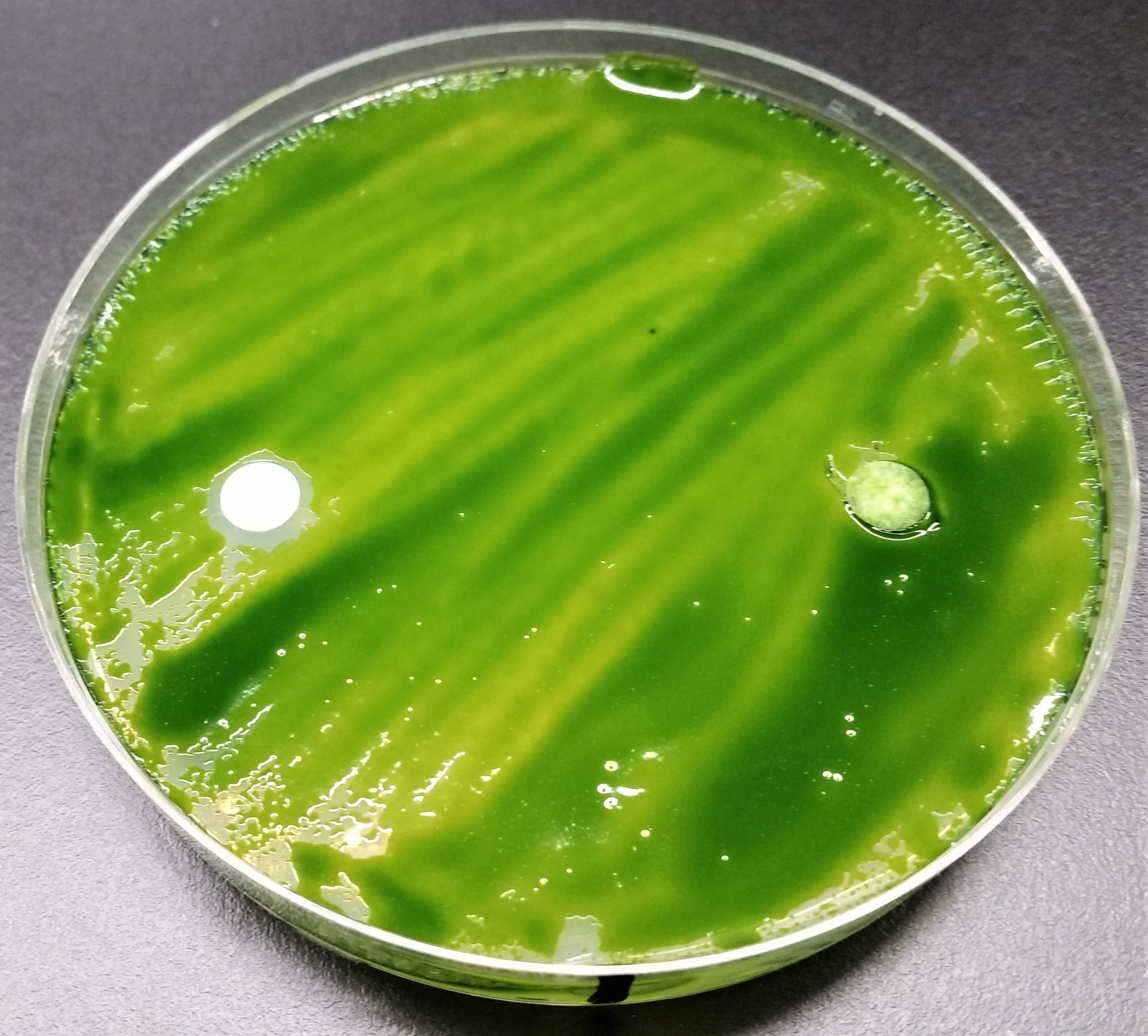

Supplement: Images of antibiotic plates of the bacterial strain CC4533 (Sphingobium yanoikuyae PR86 strain variant partial 16S rRNA sequence; GenBank Accession # MN633285.1) and green micro-alga Chlamydomonas from the antibiotic susceptibility disc diffusion tests. — The file contains 16 images of antibiotic plates used for the antibiotic susceptibility tests using the disc diffusion method for Chlamydomonas and the bacterial strain, CC4533 (Sphingobium yanoikuyae PR86 strain variant). Antibiotics tested are: penicillin, chloramphenicol, polymyxin B and neomycin. Two different doses of antibiotics were used: 50 and 100 micrograms of each antibiotics. On the antibiotic plates, the filter paper disc on the left contains the antibiotic and that on the right contains sterile water (control). CC4533 (Sphingobium yanoikuyae PR86 strain variant) plates were imaged after 3 days of growth and Chlamydomonas plates were imaged after 4 days of growth at room temperature (22C). [file f1000research-9-27904-s0001.tgz › 50micropolymyxinB2ndbatchcropped.jpg]

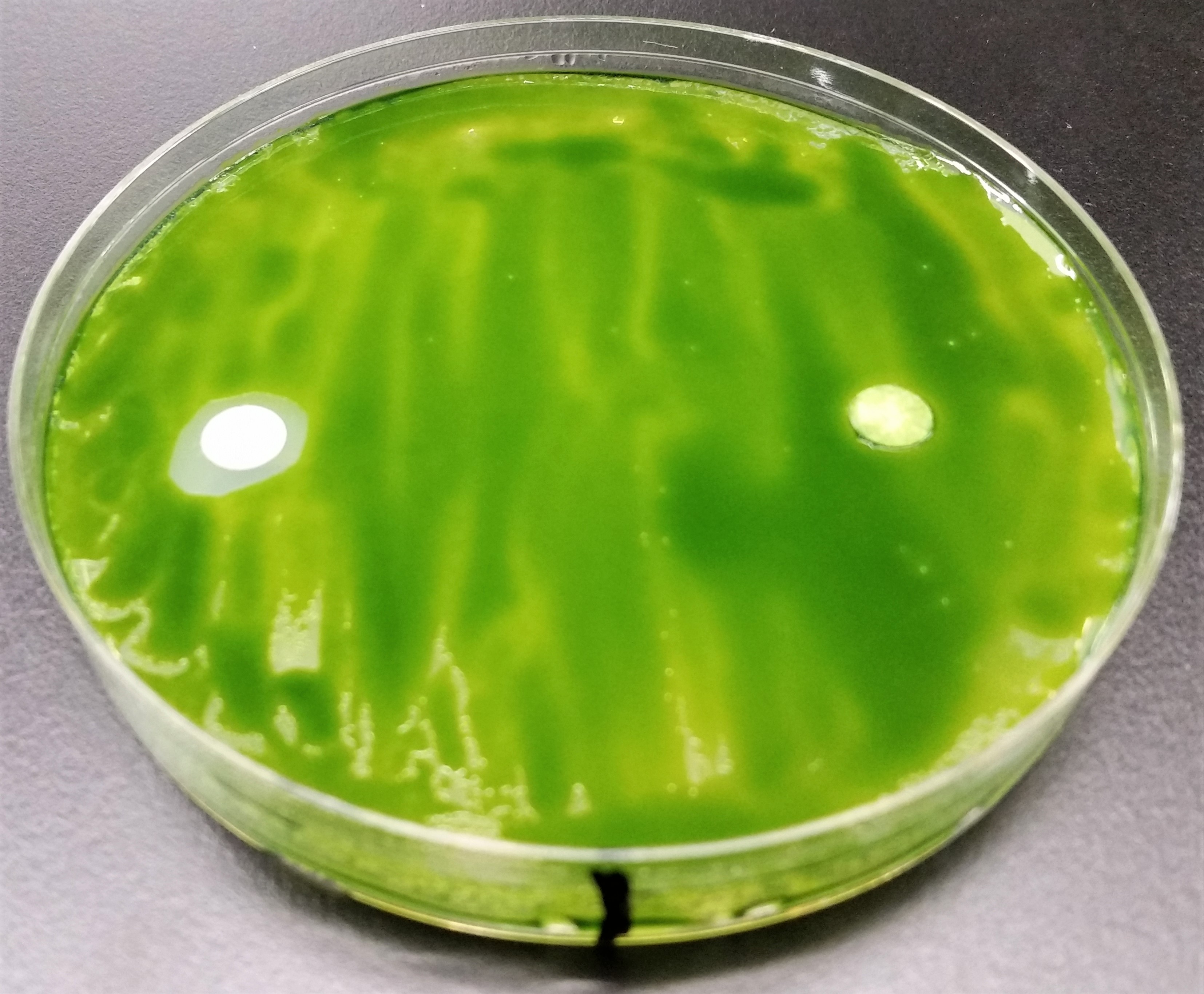

Supplement: Images of antibiotic plates of the bacterial strain CC4533 (Sphingobium yanoikuyae PR86 strain variant partial 16S rRNA sequence; GenBank Accession # MN633285.1) and green micro-alga Chlamydomonas from the antibiotic susceptibility disc diffusion tests. — The file contains 16 images of antibiotic plates used for the antibiotic susceptibility tests using the disc diffusion method for Chlamydomonas and the bacterial strain, CC4533 (Sphingobium yanoikuyae PR86 strain variant). Antibiotics tested are: penicillin, chloramphenicol, polymyxin B and neomycin. Two different doses of antibiotics were used: 50 and 100 micrograms of each antibiotics. On the antibiotic plates, the filter paper disc on the left contains the antibiotic and that on the right contains sterile water (control). CC4533 (Sphingobium yanoikuyae PR86 strain variant) plates were imaged after 3 days of growth and Chlamydomonas plates were imaged after 4 days of growth at room temperature (22C). [file f1000research-9-27904-s0001.tgz › 100micropolymyxinB2ndbatchcropped.jpg]

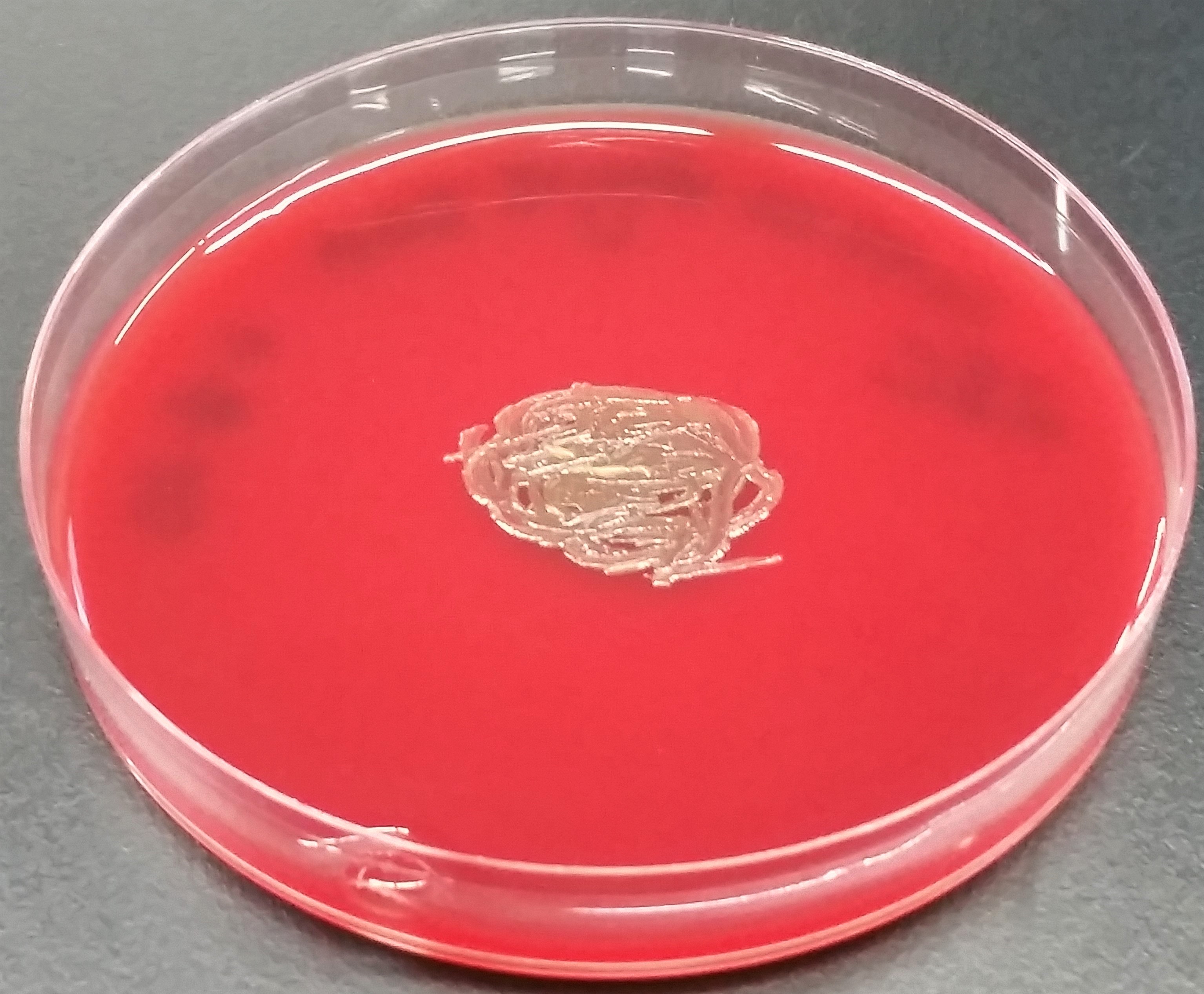

Supplement: Growth of the bacterial strain CC4533 (Sphingobium yanoikuyae PR86 strain variant partial 16S rRNA sequence; GenBank Accession # MN633285.1) and Staphylococcus aureus on Tryptic Soy agar medium containing 5% sheep blood. — Six figures in this file show the growth of the bacterial strain CC4533 (Sphingobium yanoikuyae PR86 strain variant) and Staphylococcus aureus on Tryptic Soy agar medium plates containing 5% sheep blood (Carolina Biological, Burlington, NC) over a period of 3 days at 30C. Plates were imaged after every 24 hours. Sphingobium yanoikuyae PR86 strain variant did not show any alpha hemolysis after 24 hours. Dark brown coloration on the blood agar medium around the growth of Sphingobium yanoikuyae PR86 strain variant was observed after 48 hours of growth. This brown coloration became more pronounced after 72 hours of growth. S. aureus is beta hemolytic and showed clear zones around its growth on blood agar after 24 hours of growth. [file f1000research-9-27904-s0002.tgz › CC453324hrsBA30C.jpg]

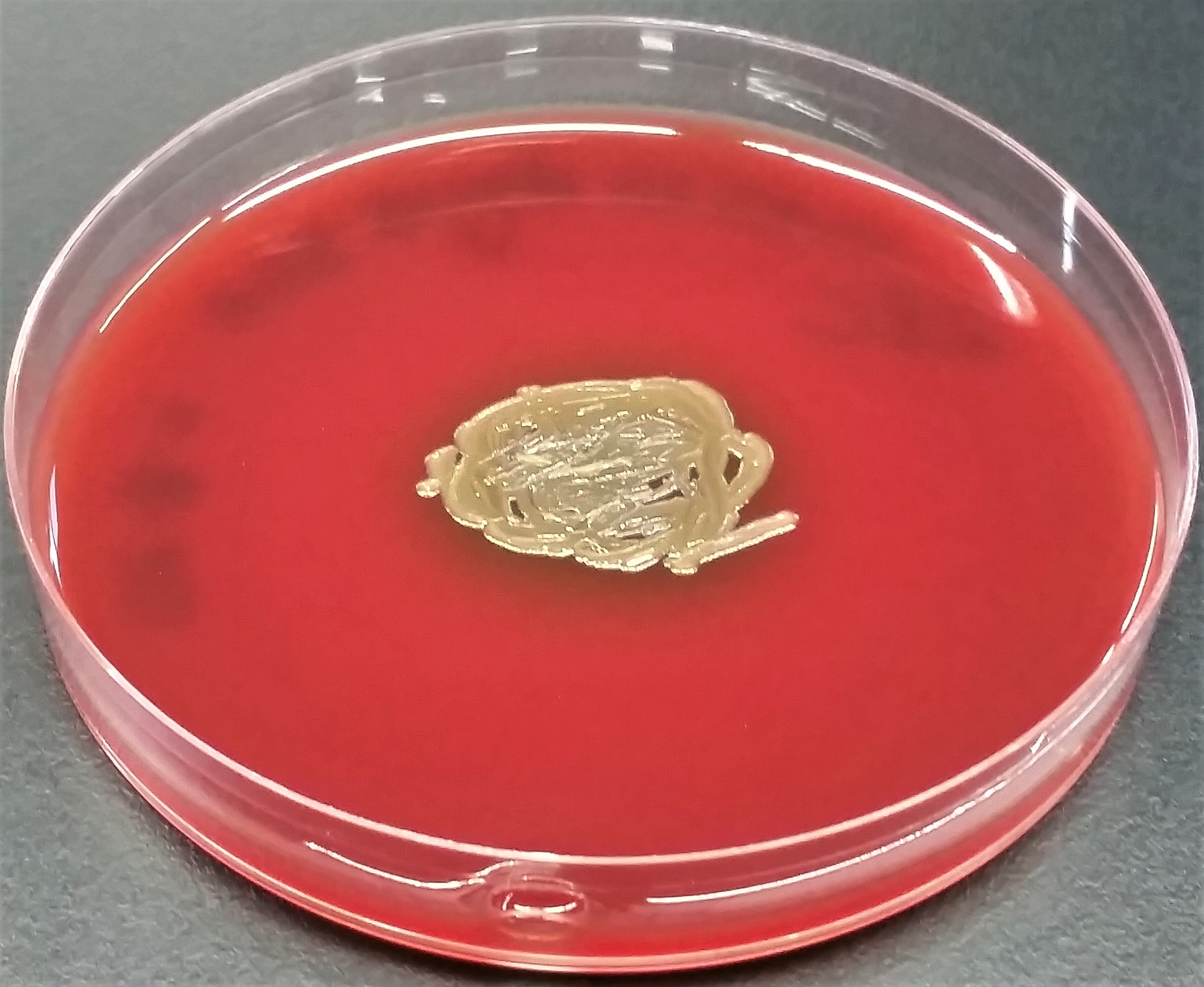

Supplement: Growth of the bacterial strain CC4533 (Sphingobium yanoikuyae PR86 strain variant partial 16S rRNA sequence; GenBank Accession # MN633285.1) and Staphylococcus aureus on Tryptic Soy agar medium containing 5% sheep blood. — Six figures in this file show the growth of the bacterial strain CC4533 (Sphingobium yanoikuyae PR86 strain variant) and Staphylococcus aureus on Tryptic Soy agar medium plates containing 5% sheep blood (Carolina Biological, Burlington, NC) over a period of 3 days at 30C. Plates were imaged after every 24 hours. Sphingobium yanoikuyae PR86 strain variant did not show any alpha hemolysis after 24 hours. Dark brown coloration on the blood agar medium around the growth of Sphingobium yanoikuyae PR86 strain variant was observed after 48 hours of growth. This brown coloration became more pronounced after 72 hours of growth. S. aureus is beta hemolytic and showed clear zones around its growth on blood agar after 24 hours of growth. [file f1000research-9-27904-s0002.tgz › CC453348hrsBA30C.jpg]

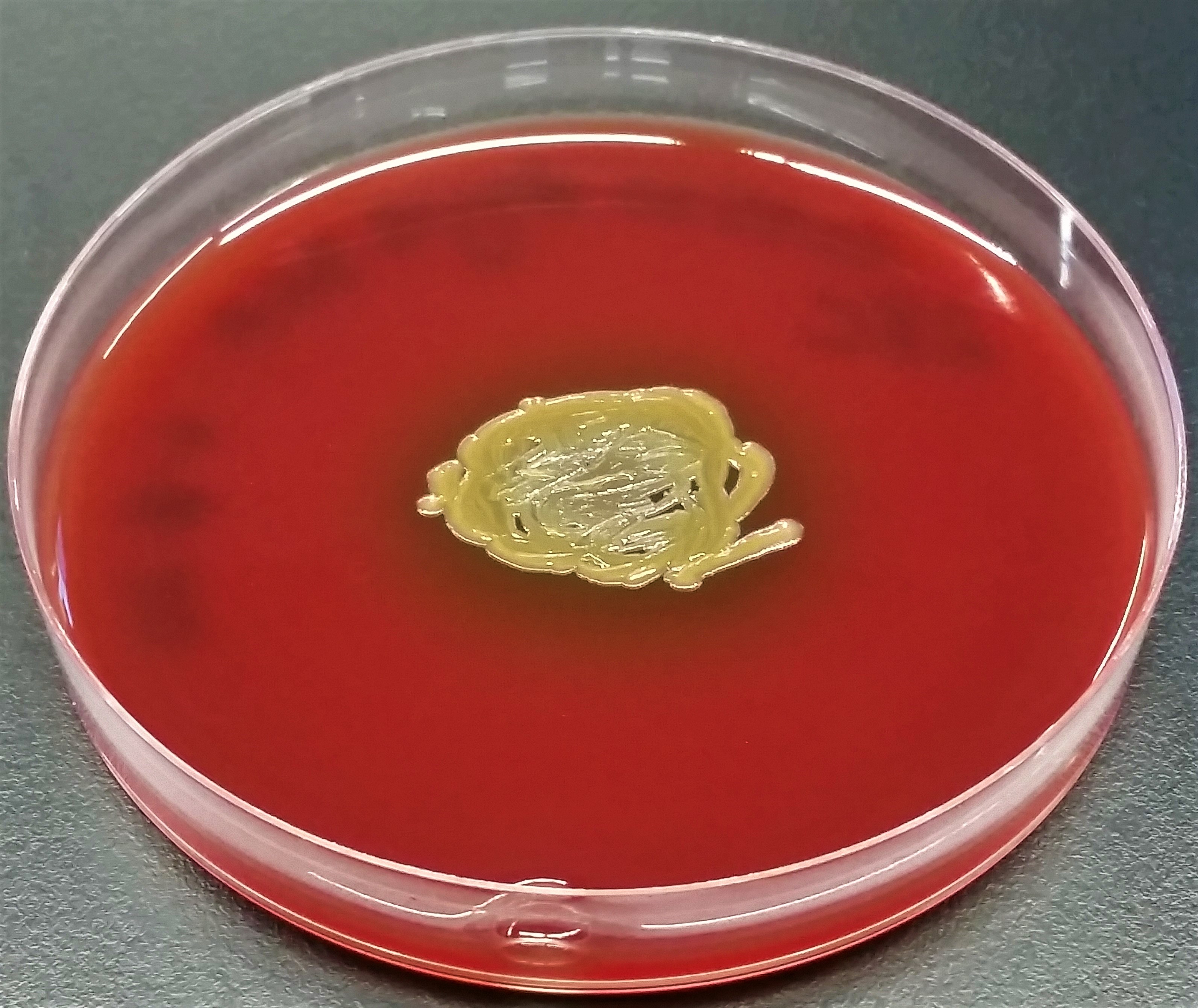

Supplement: Growth of the bacterial strain CC4533 (Sphingobium yanoikuyae PR86 strain variant partial 16S rRNA sequence; GenBank Accession # MN633285.1) and Staphylococcus aureus on Tryptic Soy agar medium containing 5% sheep blood. — Six figures in this file show the growth of the bacterial strain CC4533 (Sphingobium yanoikuyae PR86 strain variant) and Staphylococcus aureus on Tryptic Soy agar medium plates containing 5% sheep blood (Carolina Biological, Burlington, NC) over a period of 3 days at 30C. Plates were imaged after every 24 hours. Sphingobium yanoikuyae PR86 strain variant did not show any alpha hemolysis after 24 hours. Dark brown coloration on the blood agar medium around the growth of Sphingobium yanoikuyae PR86 strain variant was observed after 48 hours of growth. This brown coloration became more pronounced after 72 hours of growth. S. aureus is beta hemolytic and showed clear zones around its growth on blood agar after 24 hours of growth. [file f1000research-9-27904-s0002.tgz › CC453372hrsBA30C.jpg]

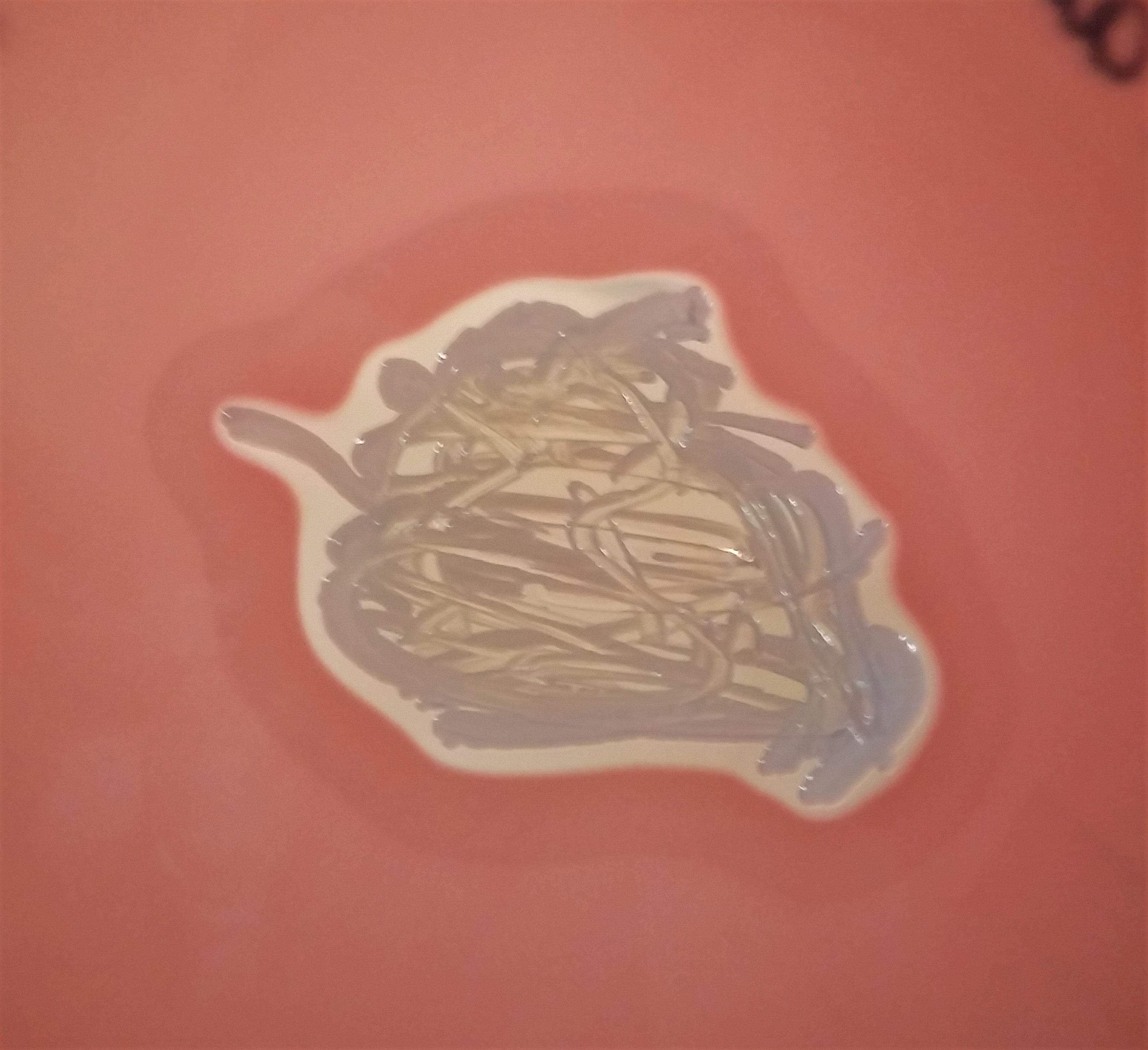

Supplement: Growth of the bacterial strain CC4533 (Sphingobium yanoikuyae PR86 strain variant partial 16S rRNA sequence; GenBank Accession # MN633285.1) and Staphylococcus aureus on Tryptic Soy agar medium containing 5% sheep blood. — Six figures in this file show the growth of the bacterial strain CC4533 (Sphingobium yanoikuyae PR86 strain variant) and Staphylococcus aureus on Tryptic Soy agar medium plates containing 5% sheep blood (Carolina Biological, Burlington, NC) over a period of 3 days at 30C. Plates were imaged after every 24 hours. Sphingobium yanoikuyae PR86 strain variant did not show any alpha hemolysis after 24 hours. Dark brown coloration on the blood agar medium around the growth of Sphingobium yanoikuyae PR86 strain variant was observed after 48 hours of growth. This brown coloration became more pronounced after 72 hours of growth. S. aureus is beta hemolytic and showed clear zones around its growth on blood agar after 24 hours of growth. [file f1000research-9-27904-s0002.tgz › S.aureus24hrsBA30C.jpg]

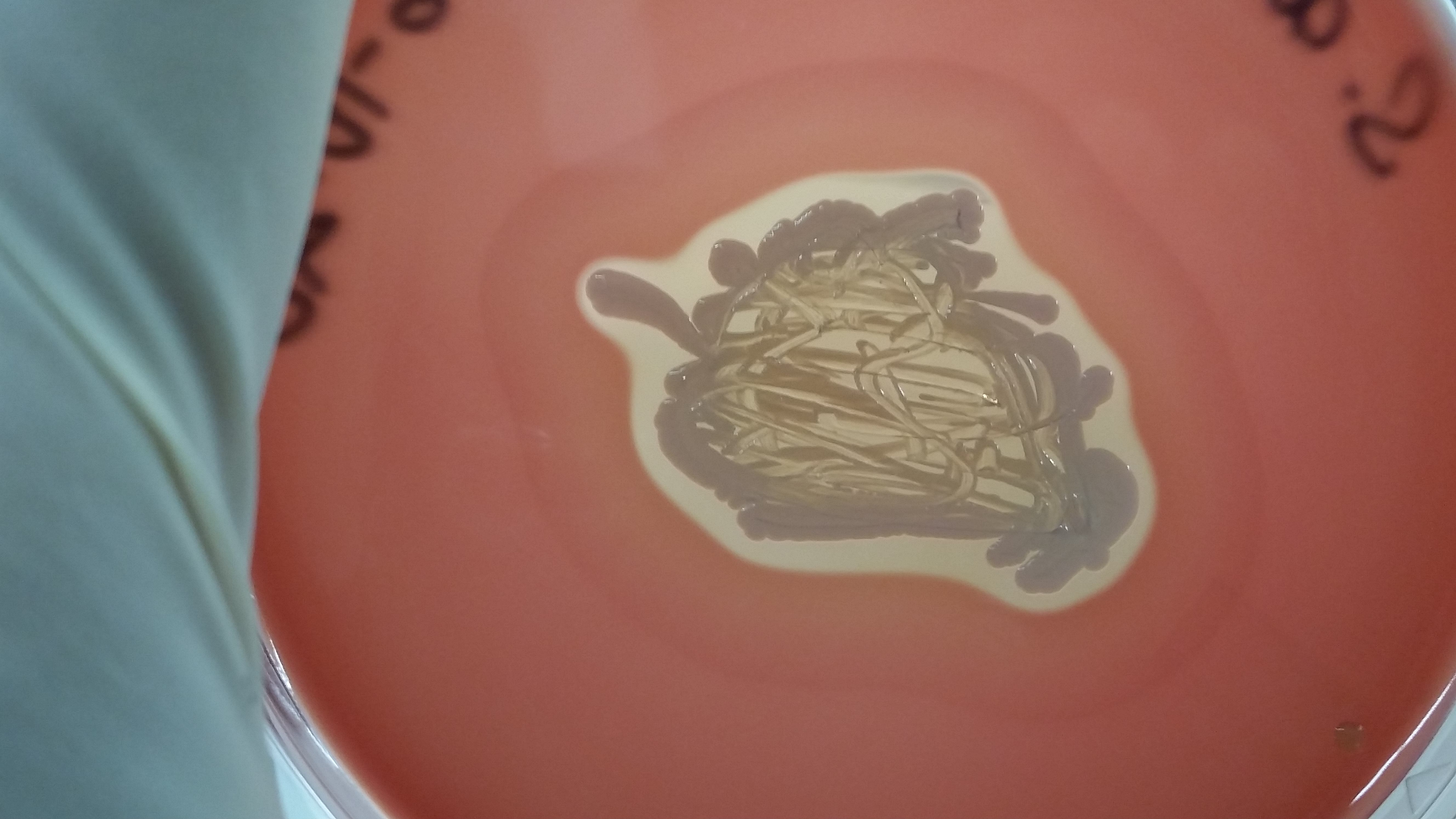

Supplement: Growth of the bacterial strain CC4533 (Sphingobium yanoikuyae PR86 strain variant partial 16S rRNA sequence; GenBank Accession # MN633285.1) and Staphylococcus aureus on Tryptic Soy agar medium containing 5% sheep blood. — Six figures in this file show the growth of the bacterial strain CC4533 (Sphingobium yanoikuyae PR86 strain variant) and Staphylococcus aureus on Tryptic Soy agar medium plates containing 5% sheep blood (Carolina Biological, Burlington, NC) over a period of 3 days at 30C. Plates were imaged after every 24 hours. Sphingobium yanoikuyae PR86 strain variant did not show any alpha hemolysis after 24 hours. Dark brown coloration on the blood agar medium around the growth of Sphingobium yanoikuyae PR86 strain variant was observed after 48 hours of growth. This brown coloration became more pronounced after 72 hours of growth. S. aureus is beta hemolytic and showed clear zones around its growth on blood agar after 24 hours of growth. [file f1000research-9-27904-s0002.tgz › S.aureus48hrsBA30C.jpg]

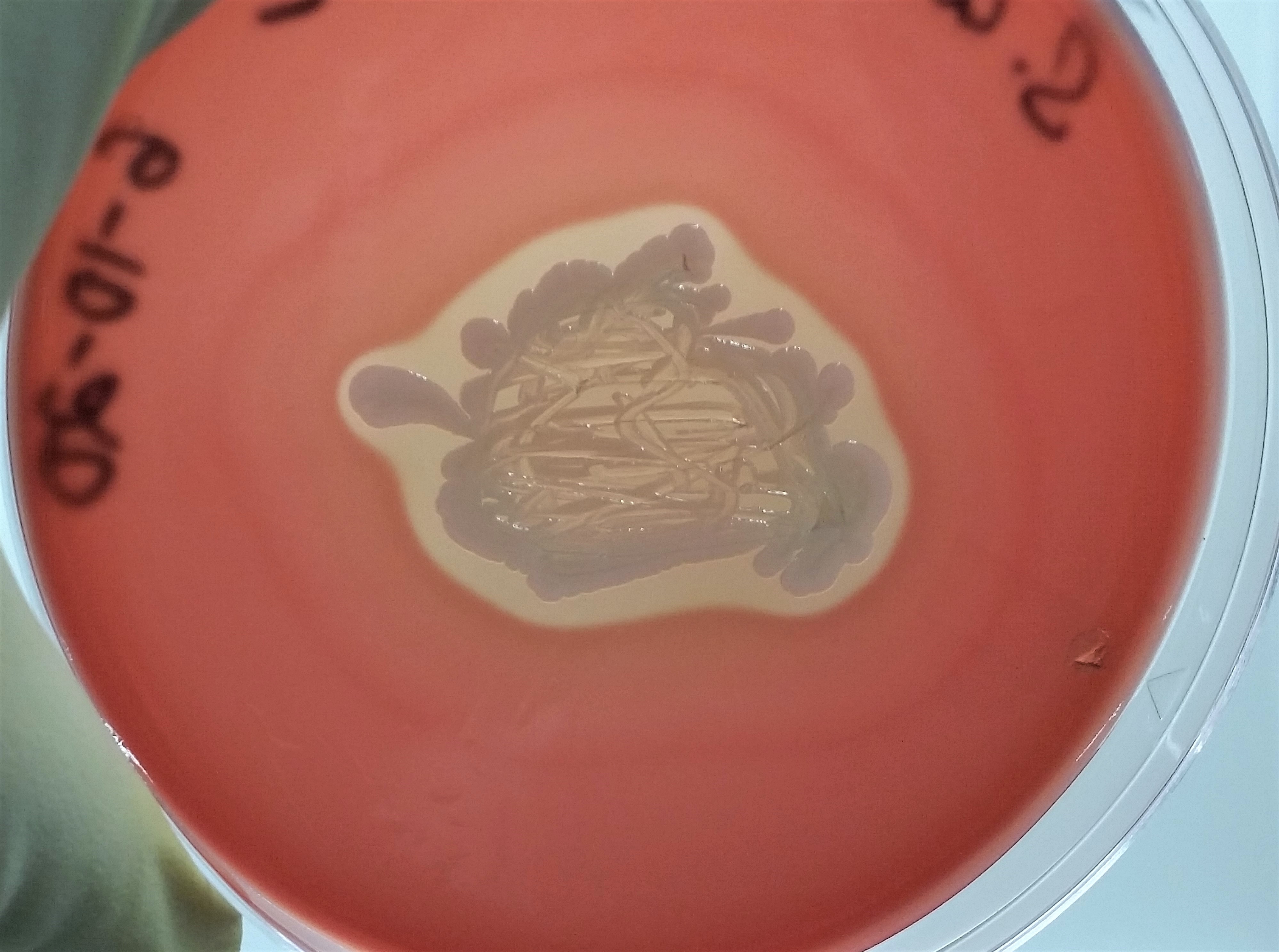

Supplement: Growth of the bacterial strain CC4533 (Sphingobium yanoikuyae PR86 strain variant partial 16S rRNA sequence; GenBank Accession # MN633285.1) and Staphylococcus aureus on Tryptic Soy agar medium containing 5% sheep blood. — Six figures in this file show the growth of the bacterial strain CC4533 (Sphingobium yanoikuyae PR86 strain variant) and Staphylococcus aureus on Tryptic Soy agar medium plates containing 5% sheep blood (Carolina Biological, Burlington, NC) over a period of 3 days at 30C. Plates were imaged after every 24 hours. Sphingobium yanoikuyae PR86 strain variant did not show any alpha hemolysis after 24 hours. Dark brown coloration on the blood agar medium around the growth of Sphingobium yanoikuyae PR86 strain variant was observed after 48 hours of growth. This brown coloration became more pronounced after 72 hours of growth. S. aureus is beta hemolytic and showed clear zones around its growth on blood agar after 24 hours of growth. [file f1000research-9-27904-s0002.tgz › S.aureus72hrsBA30C.jpg]

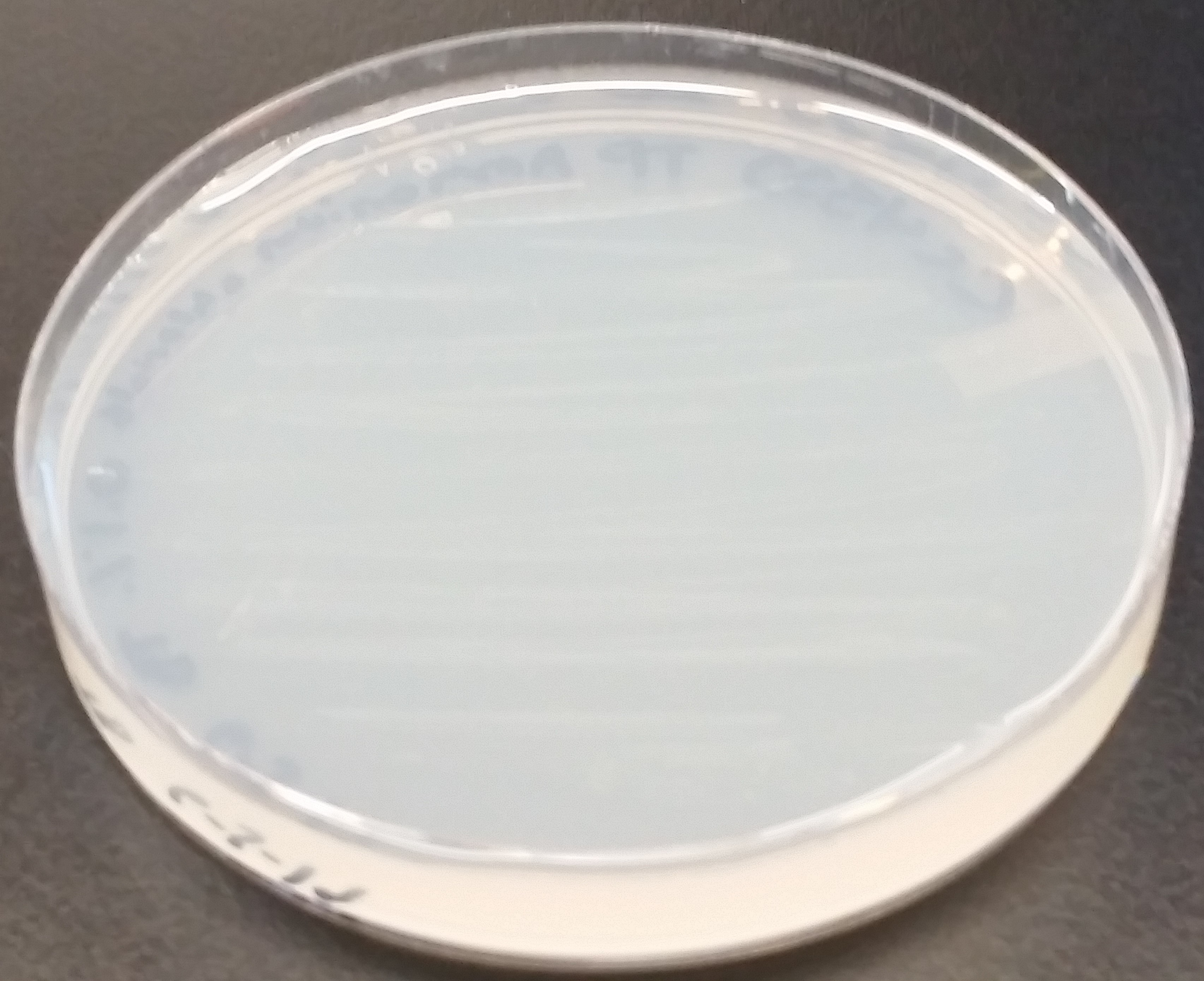

Supplement: Tests using Tris-Phosphate medium (TP) to see if hydrocarbons, aromatic compounds and polyhydroxyalkanoates can be used by the bacterium CC4533 (Sphingobium yanoikuyae PR86 strain variant, partial 16S rRNA sequence; GenBank Accession # MN633285.1) as the sole carbon source. — The file contains 21 images of TP (Tris-Phosphate) medium plates containing different alternative carbon sources. Bacterium CC4533 (Sphingobium yanoikuyae PR86 strain variant) was streaked on these chemical plates to test if CC4533 can utilize these chemicals as the sole carbon source for energy and growth. 1% stocks of the following chemicals were tested: cyclohexyl chloride, phenanthrene, napthalene, benzoic acid and phenyl acetate. 2% (v/v) stocks of fresh and used car motor oil 10W30 were also tested. Chemical doses used are given in mL in the file name. medium plates were imaged after two weeks of growth at room temperature (22C) [file f1000research-9-27904-s0003.tgz › CC4533TPnegcontrol1.jpg]

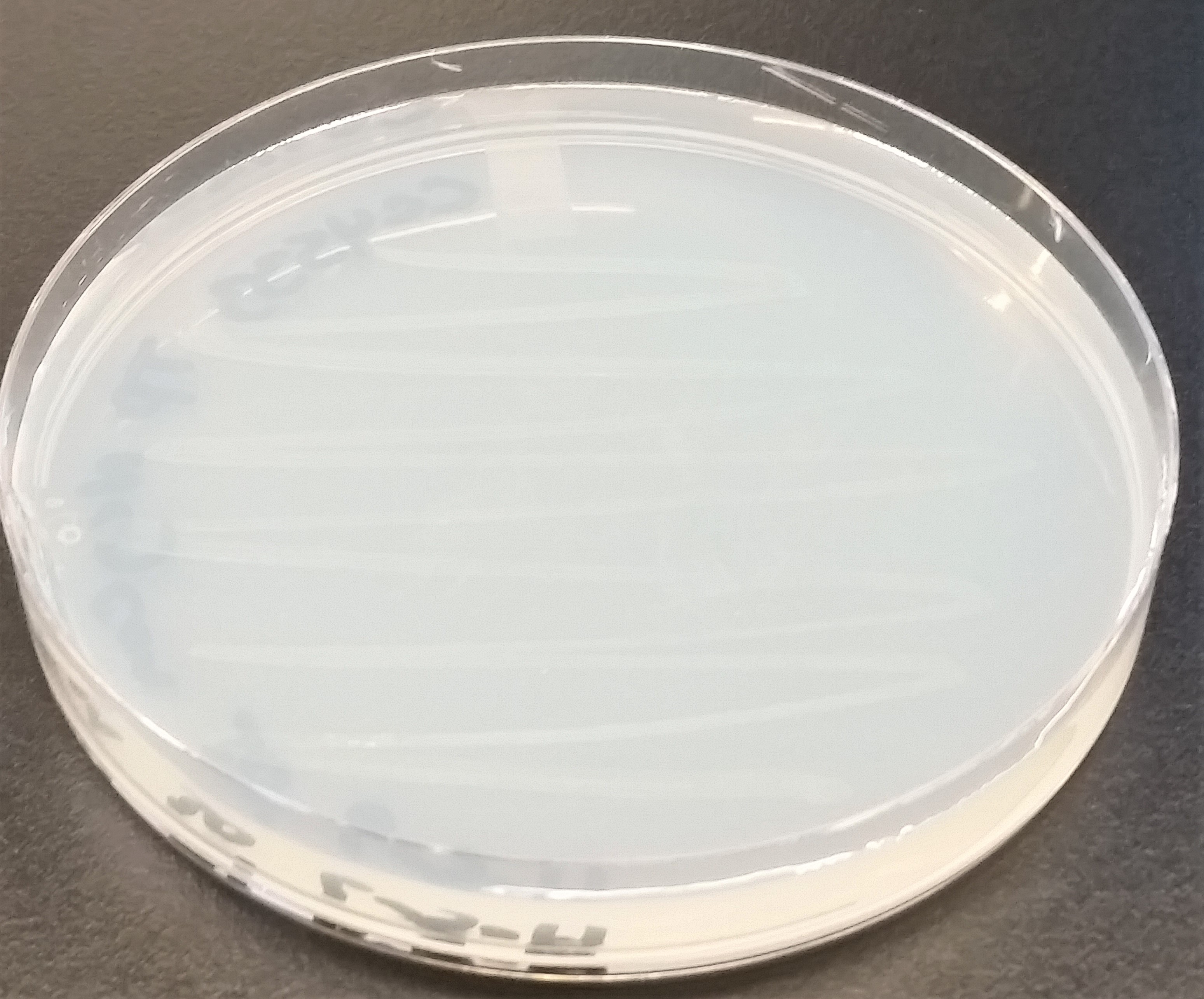

Supplement: Tests using Tris-Phosphate medium (TP) to see if hydrocarbons, aromatic compounds and polyhydroxyalkanoates can be used by the bacterium CC4533 (Sphingobium yanoikuyae PR86 strain variant, partial 16S rRNA sequence; GenBank Accession # MN633285.1) as the sole carbon source. — The file contains 21 images of TP (Tris-Phosphate) medium plates containing different alternative carbon sources. Bacterium CC4533 (Sphingobium yanoikuyae PR86 strain variant) was streaked on these chemical plates to test if CC4533 can utilize these chemicals as the sole carbon source for energy and growth. 1% stocks of the following chemicals were tested: cyclohexyl chloride, phenanthrene, napthalene, benzoic acid and phenyl acetate. 2% (v/v) stocks of fresh and used car motor oil 10W30 were also tested. Chemical doses used are given in mL in the file name. medium plates were imaged after two weeks of growth at room temperature (22C) [file f1000research-9-27904-s0003.tgz › cc4533negcontrol2TP.jpg]

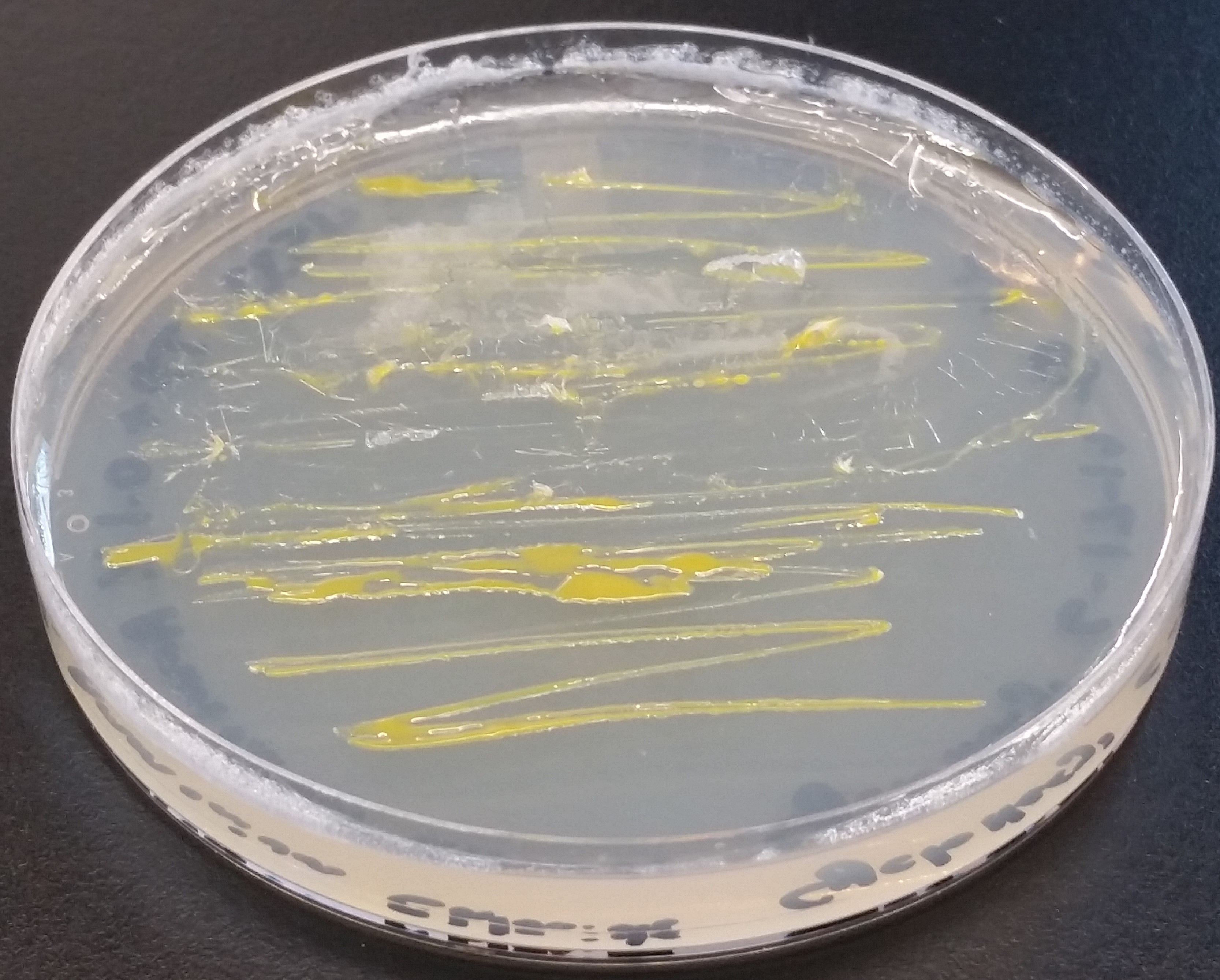

Supplement: Tests using Tris-Phosphate medium (TP) to see if hydrocarbons, aromatic compounds and polyhydroxyalkanoates can be used by the bacterium CC4533 (Sphingobium yanoikuyae PR86 strain variant, partial 16S rRNA sequence; GenBank Accession # MN633285.1) as the sole carbon source. — The file contains 21 images of TP (Tris-Phosphate) medium plates containing different alternative carbon sources. Bacterium CC4533 (Sphingobium yanoikuyae PR86 strain variant) was streaked on these chemical plates to test if CC4533 can utilize these chemicals as the sole carbon source for energy and growth. 1% stocks of the following chemicals were tested: cyclohexyl chloride, phenanthrene, napthalene, benzoic acid and phenyl acetate. 2% (v/v) stocks of fresh and used car motor oil 10W30 were also tested. Chemical doses used are given in mL in the file name. medium plates were imaged after two weeks of growth at room temperature (22C) [file f1000research-9-27904-s0003.tgz › CC45334mLof1cyclohexylchloride.jpg]

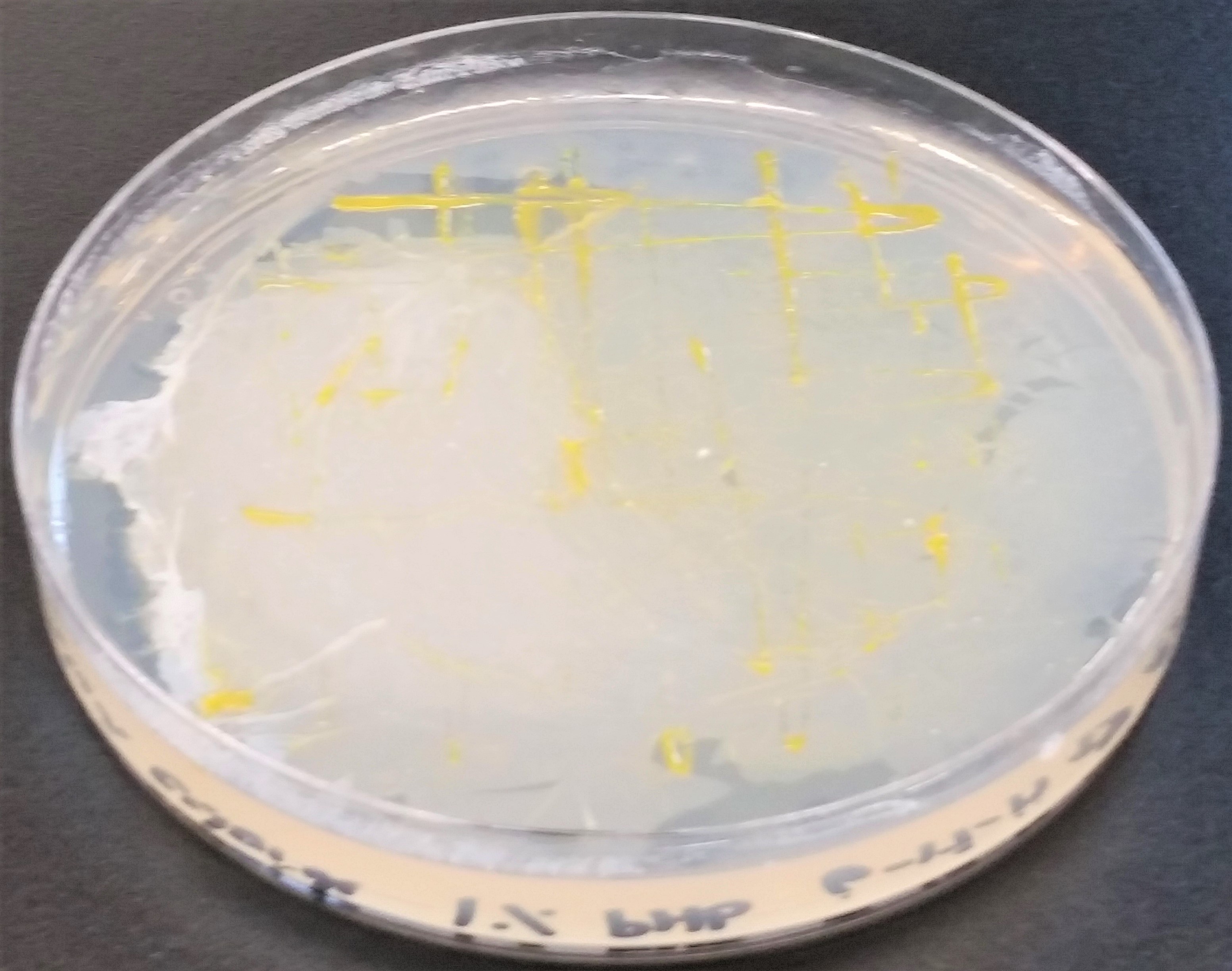

Supplement: Tests using Tris-Phosphate medium (TP) to see if hydrocarbons, aromatic compounds and polyhydroxyalkanoates can be used by the bacterium CC4533 (Sphingobium yanoikuyae PR86 strain variant, partial 16S rRNA sequence; GenBank Accession # MN633285.1) as the sole carbon source. — The file contains 21 images of TP (Tris-Phosphate) medium plates containing different alternative carbon sources. Bacterium CC4533 (Sphingobium yanoikuyae PR86 strain variant) was streaked on these chemical plates to test if CC4533 can utilize these chemicals as the sole carbon source for energy and growth. 1% stocks of the following chemicals were tested: cyclohexyl chloride, phenanthrene, napthalene, benzoic acid and phenyl acetate. 2% (v/v) stocks of fresh and used car motor oil 10W30 were also tested. Chemical doses used are given in mL in the file name. medium plates were imaged after two weeks of growth at room temperature (22C) [file f1000research-9-27904-s0003.tgz › CC45334mLof1PHB.jpg]

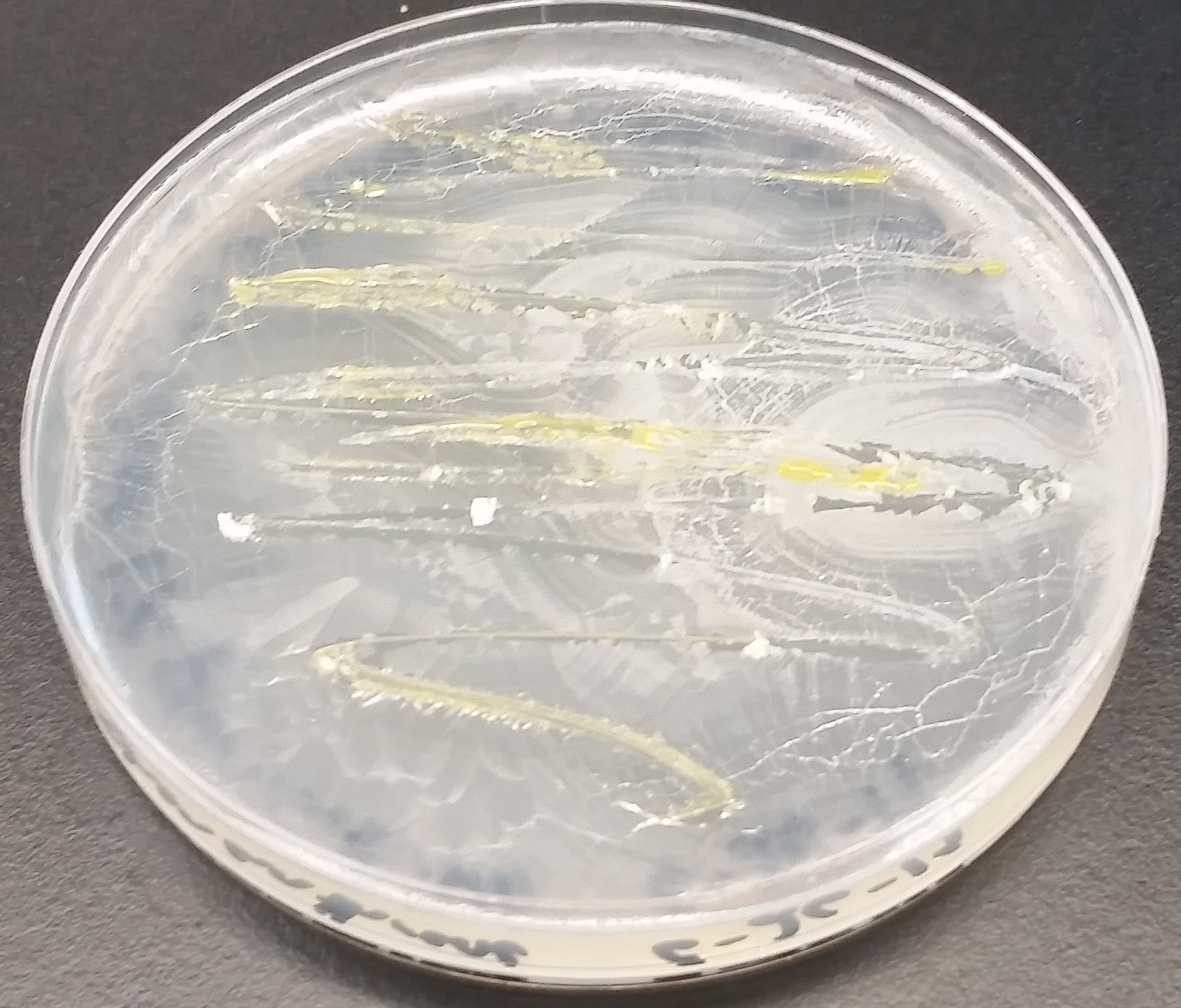

Supplement: Tests using Tris-Phosphate medium (TP) to see if hydrocarbons, aromatic compounds and polyhydroxyalkanoates can be used by the bacterium CC4533 (Sphingobium yanoikuyae PR86 strain variant, partial 16S rRNA sequence; GenBank Accession # MN633285.1) as the sole carbon source. — The file contains 21 images of TP (Tris-Phosphate) medium plates containing different alternative carbon sources. Bacterium CC4533 (Sphingobium yanoikuyae PR86 strain variant) was streaked on these chemical plates to test if CC4533 can utilize these chemicals as the sole carbon source for energy and growth. 1% stocks of the following chemicals were tested: cyclohexyl chloride, phenanthrene, napthalene, benzoic acid and phenyl acetate. 2% (v/v) stocks of fresh and used car motor oil 10W30 were also tested. Chemical doses used are given in mL in the file name. medium plates were imaged after two weeks of growth at room temperature (22C) [file f1000research-9-27904-s0003.tgz › CC45332mLof1phenanthrene.jpg]

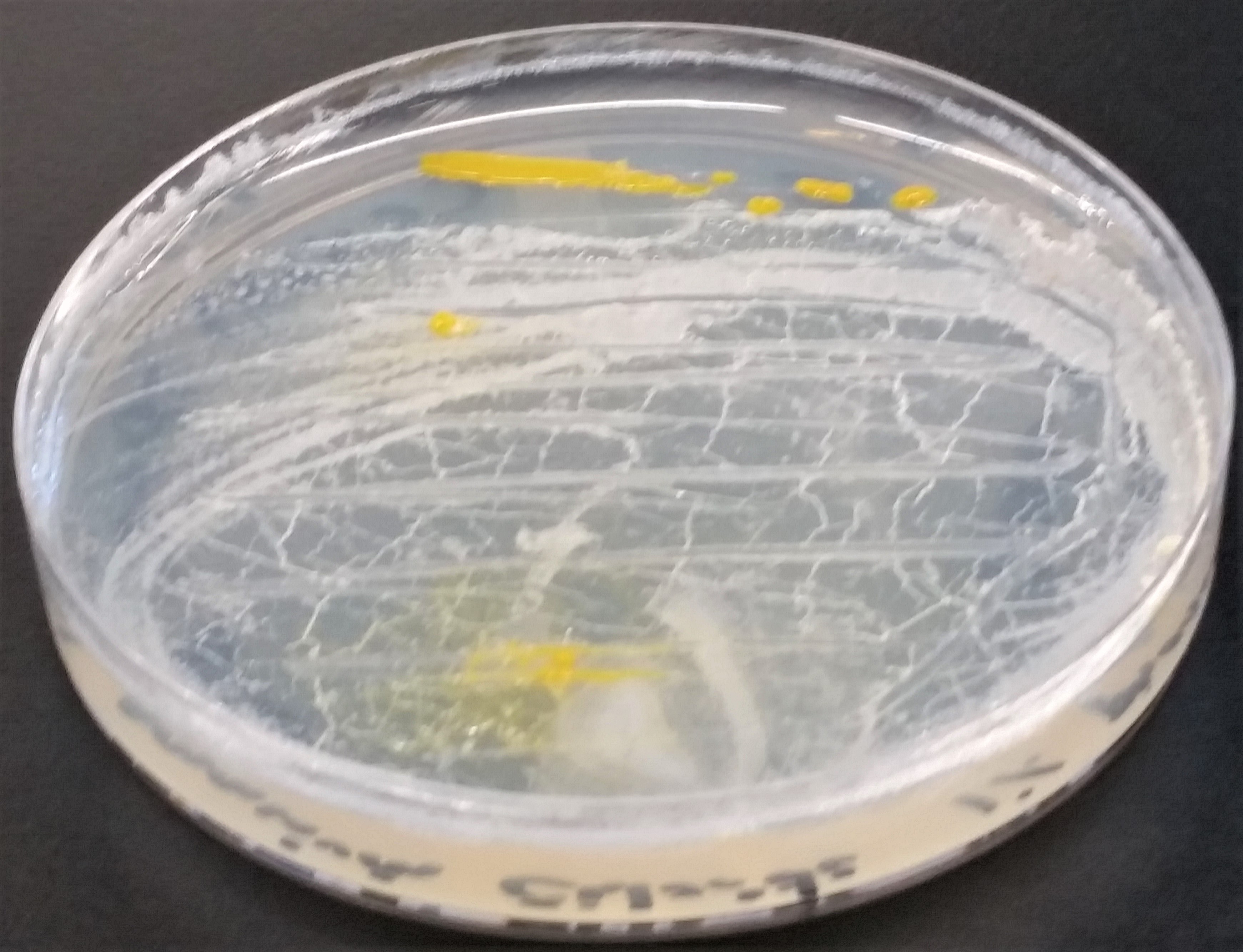

Supplement: Tests using Tris-Phosphate medium (TP) to see if hydrocarbons, aromatic compounds and polyhydroxyalkanoates can be used by the bacterium CC4533 (Sphingobium yanoikuyae PR86 strain variant, partial 16S rRNA sequence; GenBank Accession # MN633285.1) as the sole carbon source. — The file contains 21 images of TP (Tris-Phosphate) medium plates containing different alternative carbon sources. Bacterium CC4533 (Sphingobium yanoikuyae PR86 strain variant) was streaked on these chemical plates to test if CC4533 can utilize these chemicals as the sole carbon source for energy and growth. 1% stocks of the following chemicals were tested: cyclohexyl chloride, phenanthrene, napthalene, benzoic acid and phenyl acetate. 2% (v/v) stocks of fresh and used car motor oil 10W30 were also tested. Chemical doses used are given in mL in the file name. medium plates were imaged after two weeks of growth at room temperature (22C) [file f1000research-9-27904-s0003.tgz › CC45334mlof1phenanthrene.jpg]

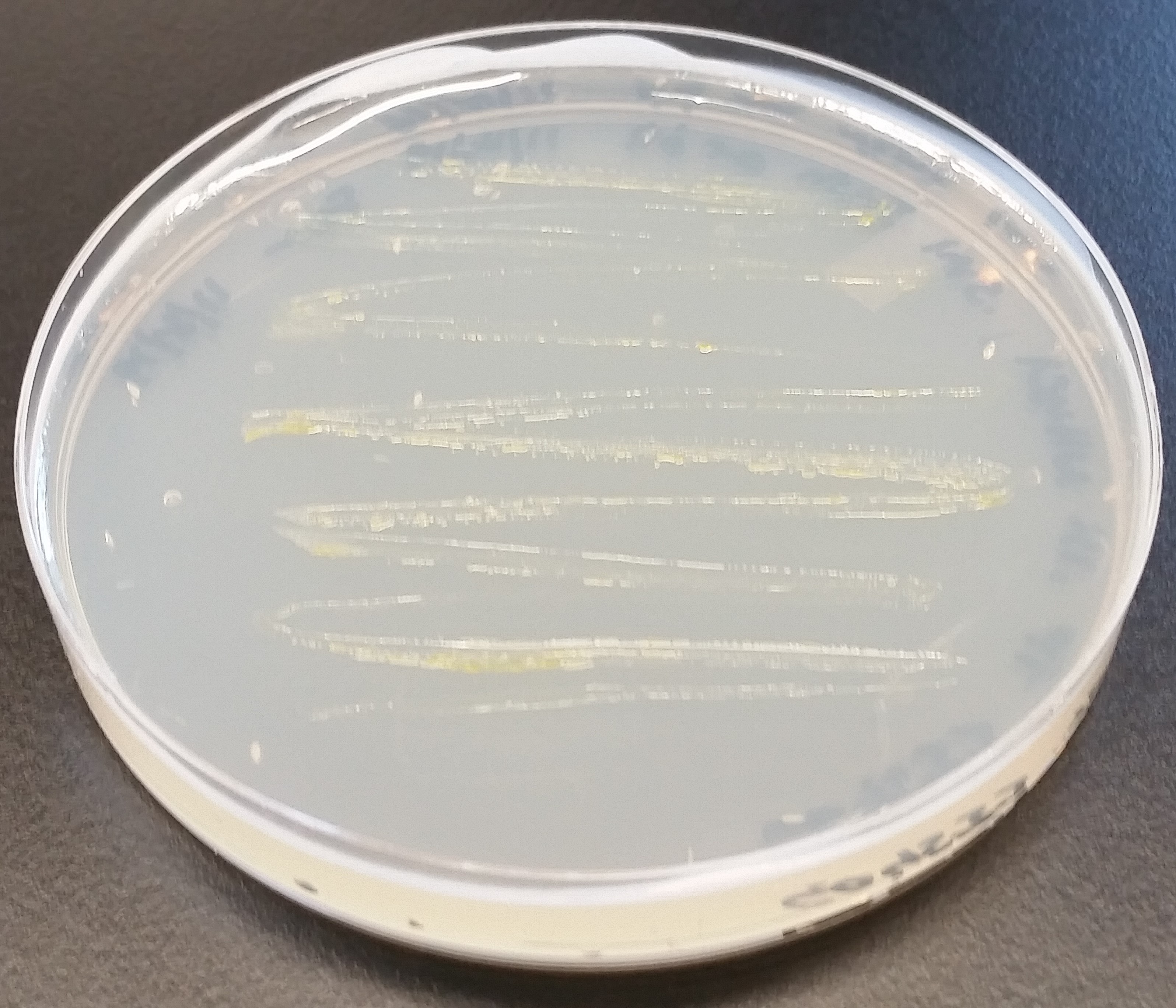

Supplement: Tests using Tris-Phosphate medium (TP) to see if hydrocarbons, aromatic compounds and polyhydroxyalkanoates can be used by the bacterium CC4533 (Sphingobium yanoikuyae PR86 strain variant, partial 16S rRNA sequence; GenBank Accession # MN633285.1) as the sole carbon source. — The file contains 21 images of TP (Tris-Phosphate) medium plates containing different alternative carbon sources. Bacterium CC4533 (Sphingobium yanoikuyae PR86 strain variant) was streaked on these chemical plates to test if CC4533 can utilize these chemicals as the sole carbon source for energy and growth. 1% stocks of the following chemicals were tested: cyclohexyl chloride, phenanthrene, napthalene, benzoic acid and phenyl acetate. 2% (v/v) stocks of fresh and used car motor oil 10W30 were also tested. Chemical doses used are given in mL in the file name. medium plates were imaged after two weeks of growth at room temperature (22C) [file f1000research-9-27904-s0003.tgz › CC45330.5mLfresh10W30.jpg]

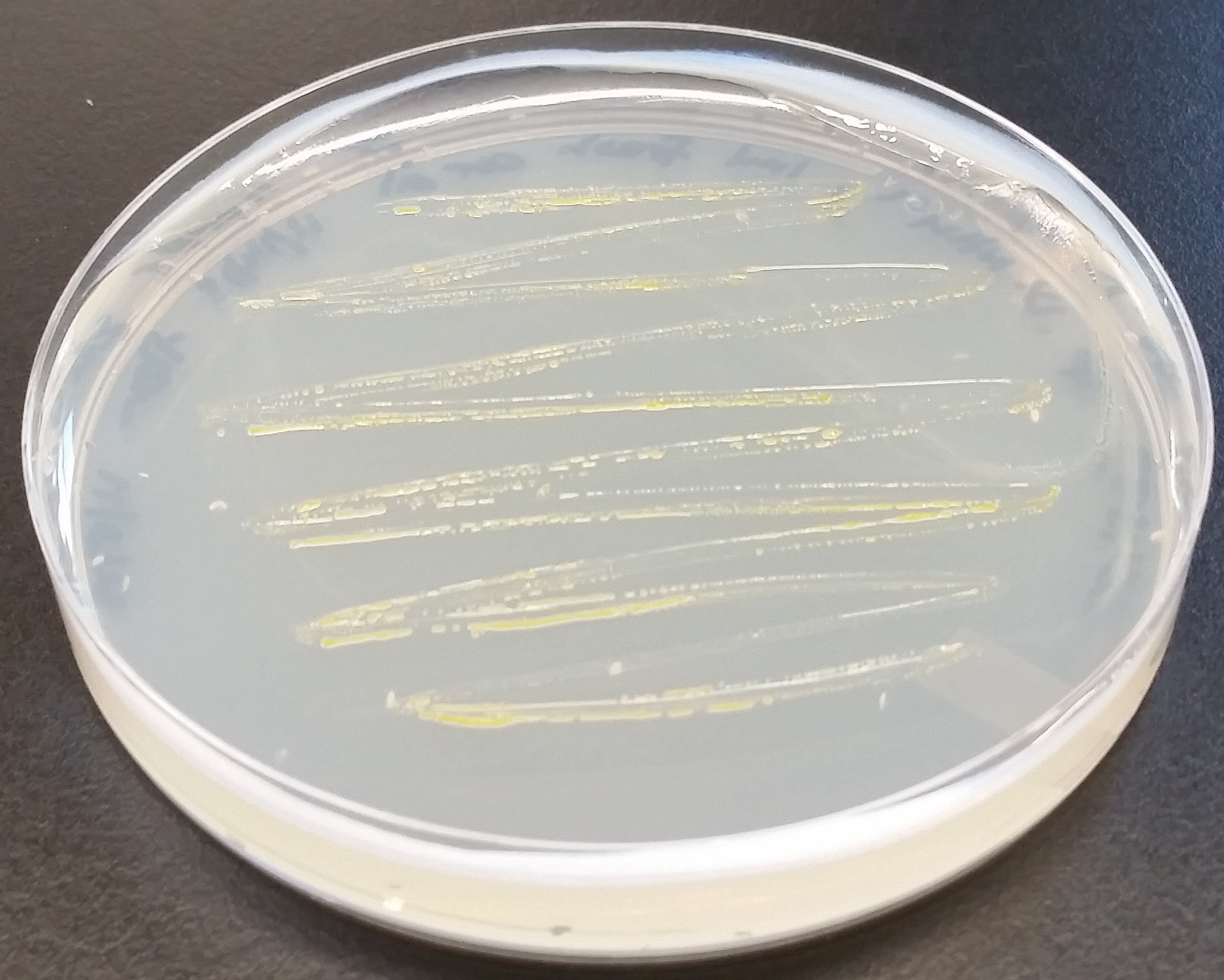

Supplement: Tests using Tris-Phosphate medium (TP) to see if hydrocarbons, aromatic compounds and polyhydroxyalkanoates can be used by the bacterium CC4533 (Sphingobium yanoikuyae PR86 strain variant, partial 16S rRNA sequence; GenBank Accession # MN633285.1) as the sole carbon source. — The file contains 21 images of TP (Tris-Phosphate) medium plates containing different alternative carbon sources. Bacterium CC4533 (Sphingobium yanoikuyae PR86 strain variant) was streaked on these chemical plates to test if CC4533 can utilize these chemicals as the sole carbon source for energy and growth. 1% stocks of the following chemicals were tested: cyclohexyl chloride, phenanthrene, napthalene, benzoic acid and phenyl acetate. 2% (v/v) stocks of fresh and used car motor oil 10W30 were also tested. Chemical doses used are given in mL in the file name. medium plates were imaged after two weeks of growth at room temperature (22C) [file f1000research-9-27904-s0003.tgz › CC4533fresh10W301mL.jpg]

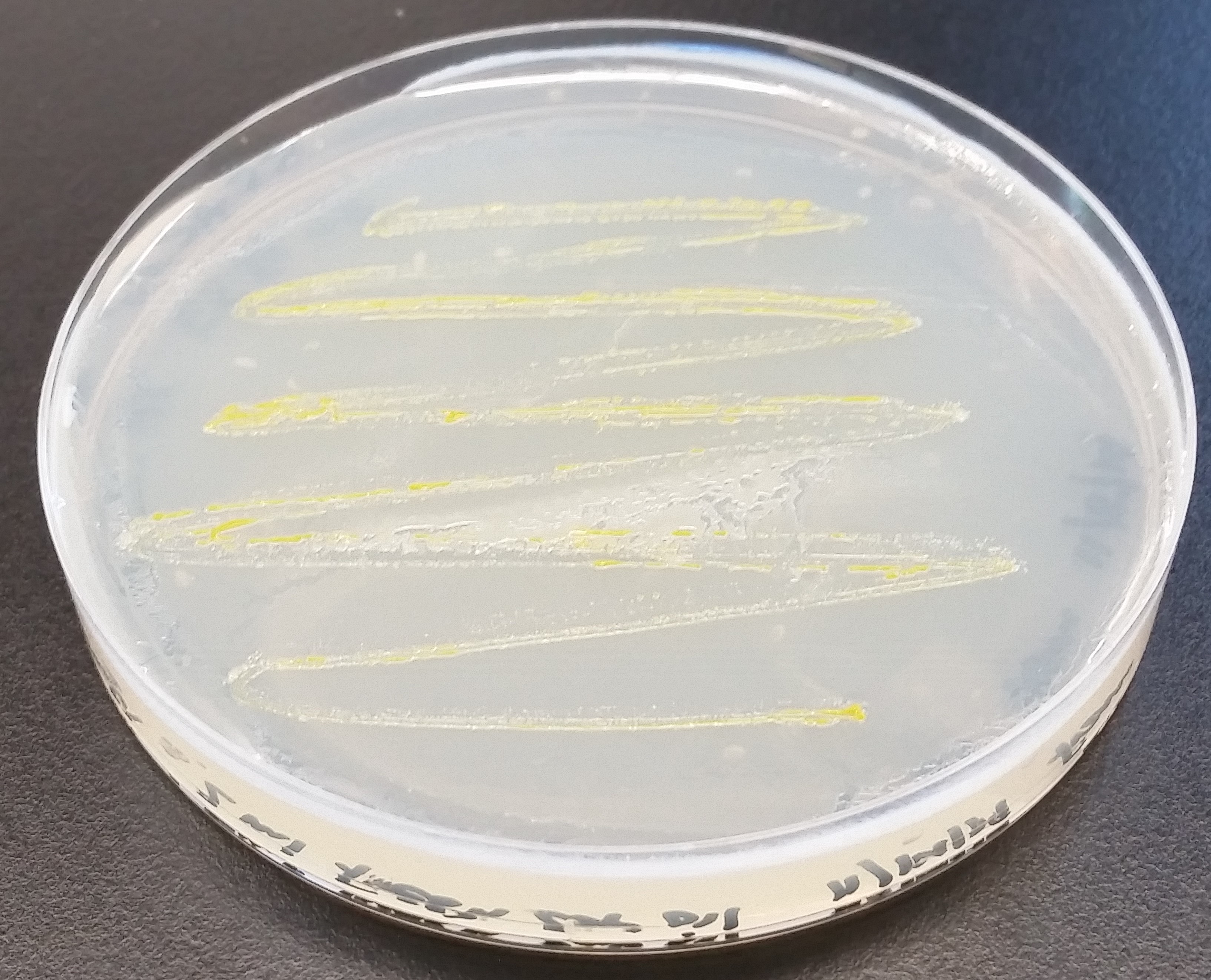

Supplement: Tests using Tris-Phosphate medium (TP) to see if hydrocarbons, aromatic compounds and polyhydroxyalkanoates can be used by the bacterium CC4533 (Sphingobium yanoikuyae PR86 strain variant, partial 16S rRNA sequence; GenBank Accession # MN633285.1) as the sole carbon source. — The file contains 21 images of TP (Tris-Phosphate) medium plates containing different alternative carbon sources. Bacterium CC4533 (Sphingobium yanoikuyae PR86 strain variant) was streaked on these chemical plates to test if CC4533 can utilize these chemicals as the sole carbon source for energy and growth. 1% stocks of the following chemicals were tested: cyclohexyl chloride, phenanthrene, napthalene, benzoic acid and phenyl acetate. 2% (v/v) stocks of fresh and used car motor oil 10W30 were also tested. Chemical doses used are given in mL in the file name. medium plates were imaged after two weeks of growth at room temperature (22C) [file f1000research-9-27904-s0003.tgz › CC4533fresh10W302mL.jpg]

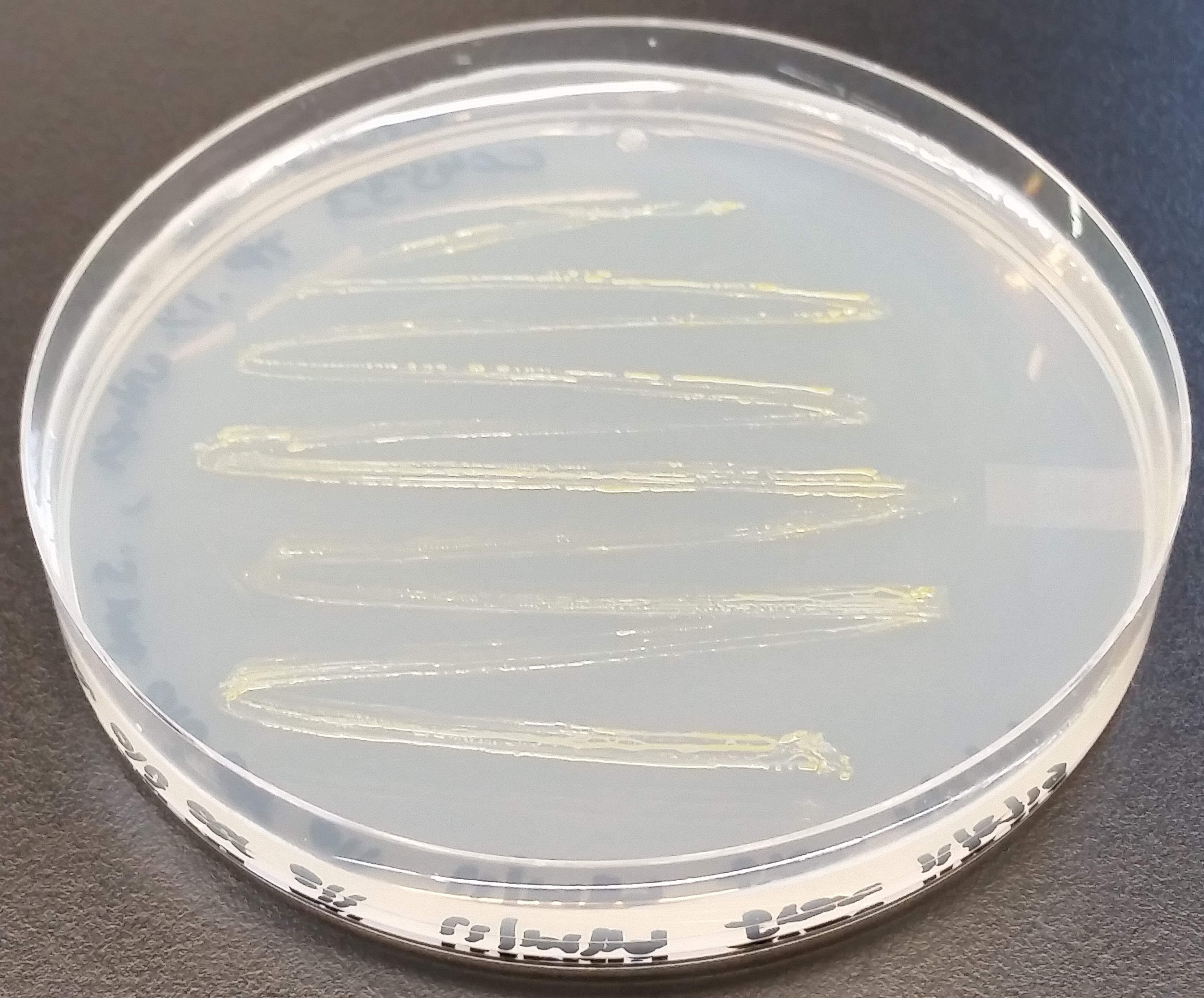

Supplement: Tests using Tris-Phosphate medium (TP) to see if hydrocarbons, aromatic compounds and polyhydroxyalkanoates can be used by the bacterium CC4533 (Sphingobium yanoikuyae PR86 strain variant, partial 16S rRNA sequence; GenBank Accession # MN633285.1) as the sole carbon source. — The file contains 21 images of TP (Tris-Phosphate) medium plates containing different alternative carbon sources. Bacterium CC4533 (Sphingobium yanoikuyae PR86 strain variant) was streaked on these chemical plates to test if CC4533 can utilize these chemicals as the sole carbon source for energy and growth. 1% stocks of the following chemicals were tested: cyclohexyl chloride, phenanthrene, napthalene, benzoic acid and phenyl acetate. 2% (v/v) stocks of fresh and used car motor oil 10W30 were also tested. Chemical doses used are given in mL in the file name. medium plates were imaged after two weeks of growth at room temperature (22C) [file f1000research-9-27904-s0003.tgz › CC4533used10W300.5mL.jpg]

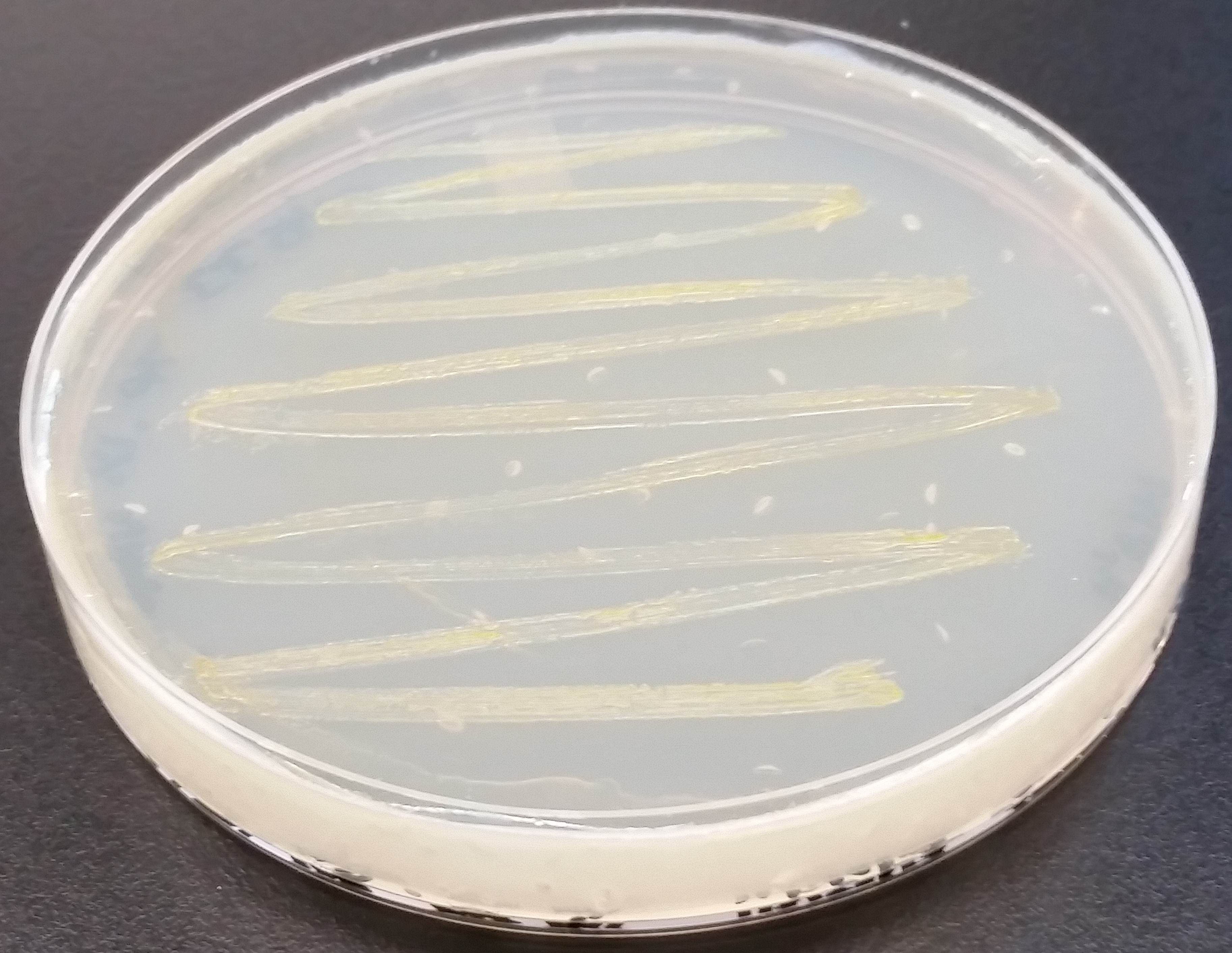

Supplement: Tests using Tris-Phosphate medium (TP) to see if hydrocarbons, aromatic compounds and polyhydroxyalkanoates can be used by the bacterium CC4533 (Sphingobium yanoikuyae PR86 strain variant, partial 16S rRNA sequence; GenBank Accession # MN633285.1) as the sole carbon source. — The file contains 21 images of TP (Tris-Phosphate) medium plates containing different alternative carbon sources. Bacterium CC4533 (Sphingobium yanoikuyae PR86 strain variant) was streaked on these chemical plates to test if CC4533 can utilize these chemicals as the sole carbon source for energy and growth. 1% stocks of the following chemicals were tested: cyclohexyl chloride, phenanthrene, napthalene, benzoic acid and phenyl acetate. 2% (v/v) stocks of fresh and used car motor oil 10W30 were also tested. Chemical doses used are given in mL in the file name. medium plates were imaged after two weeks of growth at room temperature (22C) [file f1000research-9-27904-s0003.tgz › CC4533used10W301mL.jpg]

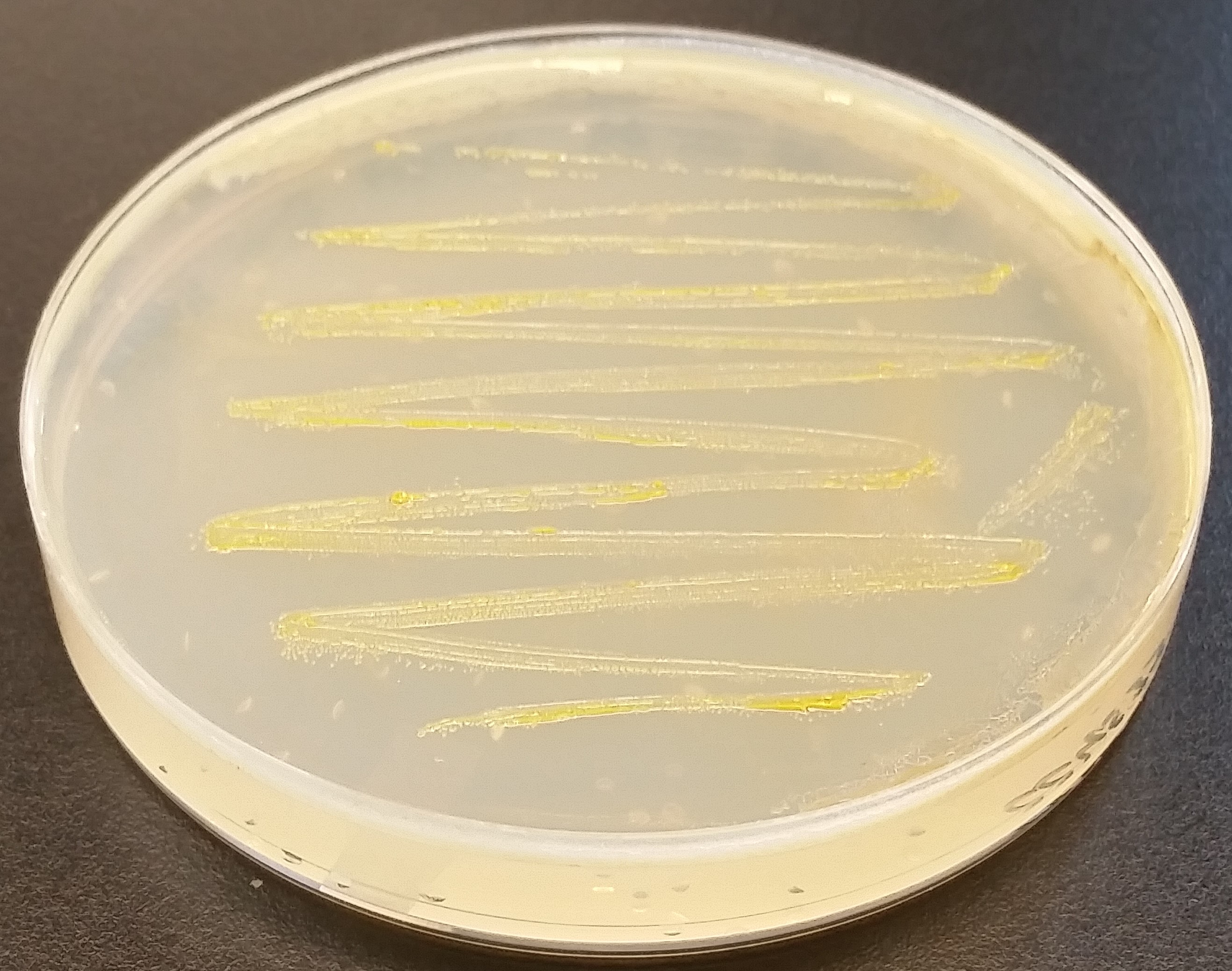

Supplement: Tests using Tris-Phosphate medium (TP) to see if hydrocarbons, aromatic compounds and polyhydroxyalkanoates can be used by the bacterium CC4533 (Sphingobium yanoikuyae PR86 strain variant, partial 16S rRNA sequence; GenBank Accession # MN633285.1) as the sole carbon source. — The file contains 21 images of TP (Tris-Phosphate) medium plates containing different alternative carbon sources. Bacterium CC4533 (Sphingobium yanoikuyae PR86 strain variant) was streaked on these chemical plates to test if CC4533 can utilize these chemicals as the sole carbon source for energy and growth. 1% stocks of the following chemicals were tested: cyclohexyl chloride, phenanthrene, napthalene, benzoic acid and phenyl acetate. 2% (v/v) stocks of fresh and used car motor oil 10W30 were also tested. Chemical doses used are given in mL in the file name. medium plates were imaged after two weeks of growth at room temperature (22C) [file f1000research-9-27904-s0003.tgz › CC4533used10W302mL.jpg]

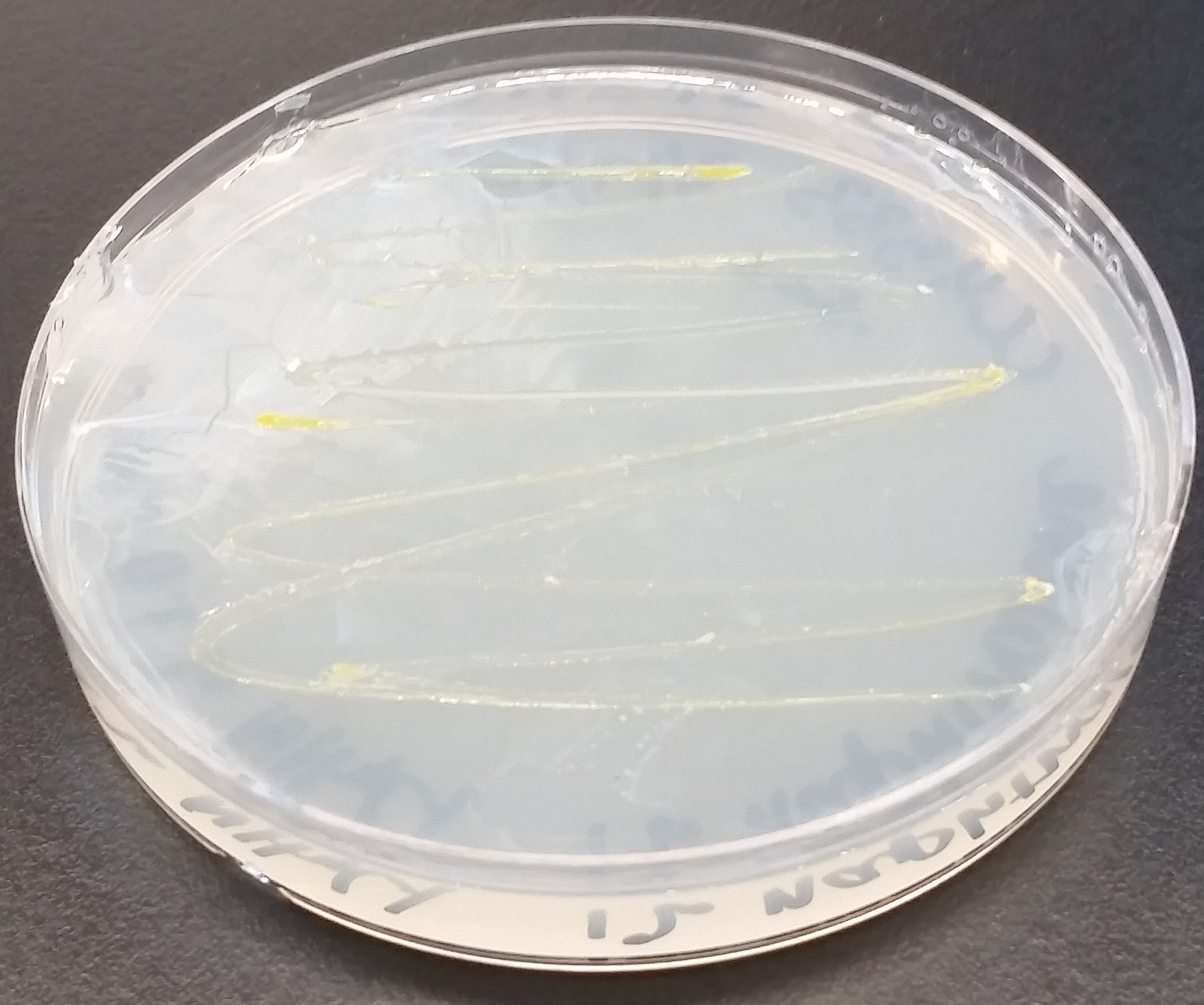

Supplement: Tests using Tris-Phosphate medium (TP) to see if hydrocarbons, aromatic compounds and polyhydroxyalkanoates can be used by the bacterium CC4533 (Sphingobium yanoikuyae PR86 strain variant, partial 16S rRNA sequence; GenBank Accession # MN633285.1) as the sole carbon source. — The file contains 21 images of TP (Tris-Phosphate) medium plates containing different alternative carbon sources. Bacterium CC4533 (Sphingobium yanoikuyae PR86 strain variant) was streaked on these chemical plates to test if CC4533 can utilize these chemicals as the sole carbon source for energy and growth. 1% stocks of the following chemicals were tested: cyclohexyl chloride, phenanthrene, napthalene, benzoic acid and phenyl acetate. 2% (v/v) stocks of fresh and used car motor oil 10W30 were also tested. Chemical doses used are given in mL in the file name. medium plates were imaged after two weeks of growth at room temperature (22C) [file f1000research-9-27904-s0003.tgz › CC45330.5mLnapthalene.jpg]

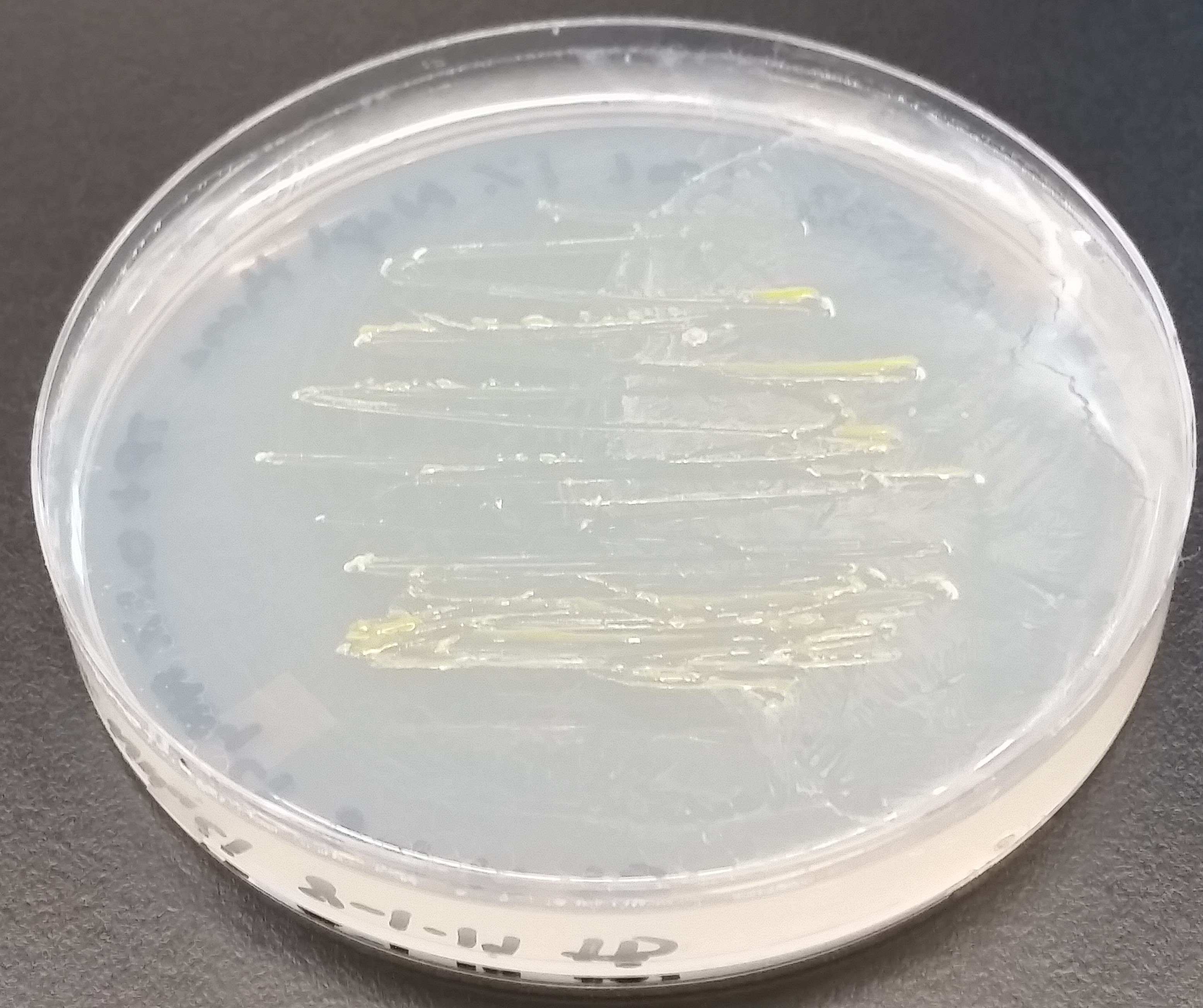

Supplement: Tests using Tris-Phosphate medium (TP) to see if hydrocarbons, aromatic compounds and polyhydroxyalkanoates can be used by the bacterium CC4533 (Sphingobium yanoikuyae PR86 strain variant, partial 16S rRNA sequence; GenBank Accession # MN633285.1) as the sole carbon source. — The file contains 21 images of TP (Tris-Phosphate) medium plates containing different alternative carbon sources. Bacterium CC4533 (Sphingobium yanoikuyae PR86 strain variant) was streaked on these chemical plates to test if CC4533 can utilize these chemicals as the sole carbon source for energy and growth. 1% stocks of the following chemicals were tested: cyclohexyl chloride, phenanthrene, napthalene, benzoic acid and phenyl acetate. 2% (v/v) stocks of fresh and used car motor oil 10W30 were also tested. Chemical doses used are given in mL in the file name. medium plates were imaged after two weeks of growth at room temperature (22C) [file f1000research-9-27904-s0003.tgz › CC45331mLnapthalene.jpg]

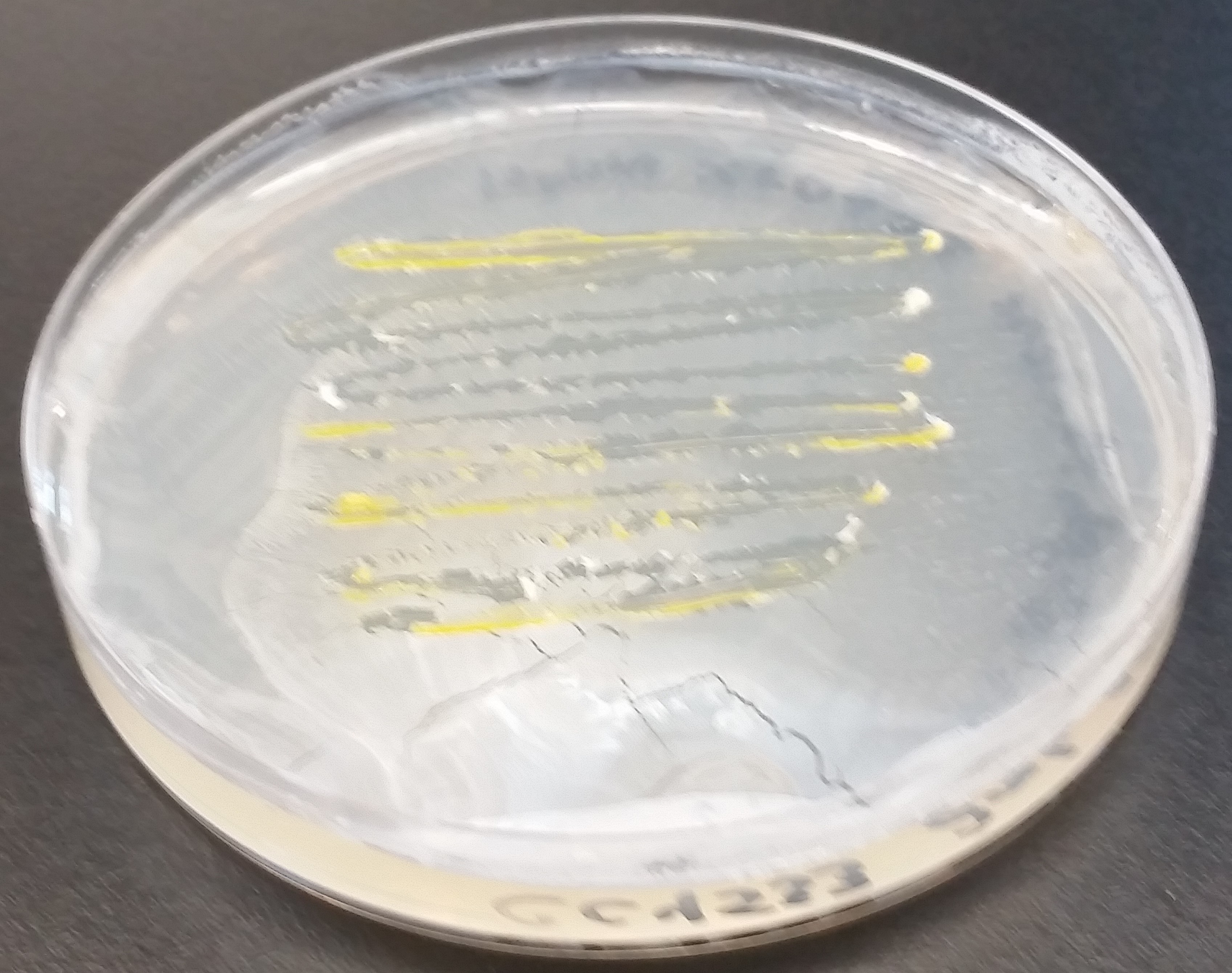

Supplement: Tests using Tris-Phosphate medium (TP) to see if hydrocarbons, aromatic compounds and polyhydroxyalkanoates can be used by the bacterium CC4533 (Sphingobium yanoikuyae PR86 strain variant, partial 16S rRNA sequence; GenBank Accession # MN633285.1) as the sole carbon source. — The file contains 21 images of TP (Tris-Phosphate) medium plates containing different alternative carbon sources. Bacterium CC4533 (Sphingobium yanoikuyae PR86 strain variant) was streaked on these chemical plates to test if CC4533 can utilize these chemicals as the sole carbon source for energy and growth. 1% stocks of the following chemicals were tested: cyclohexyl chloride, phenanthrene, napthalene, benzoic acid and phenyl acetate. 2% (v/v) stocks of fresh and used car motor oil 10W30 were also tested. Chemical doses used are given in mL in the file name. medium plates were imaged after two weeks of growth at room temperature (22C) [file f1000research-9-27904-s0003.tgz › CC45332mLnapthalene.jpg]

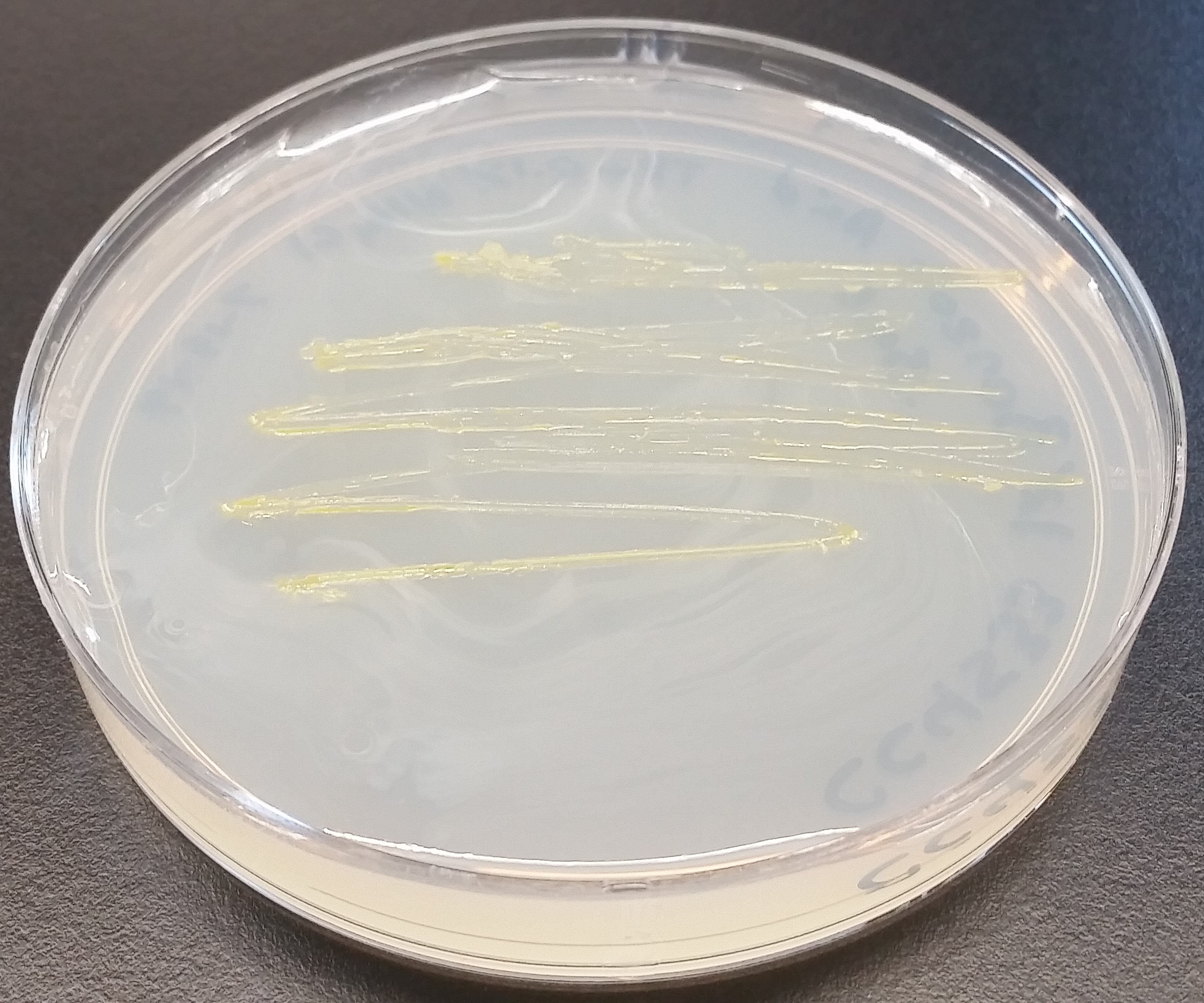

Supplement: Tests using Tris-Phosphate medium (TP) to see if hydrocarbons, aromatic compounds and polyhydroxyalkanoates can be used by the bacterium CC4533 (Sphingobium yanoikuyae PR86 strain variant, partial 16S rRNA sequence; GenBank Accession # MN633285.1) as the sole carbon source. — The file contains 21 images of TP (Tris-Phosphate) medium plates containing different alternative carbon sources. Bacterium CC4533 (Sphingobium yanoikuyae PR86 strain variant) was streaked on these chemical plates to test if CC4533 can utilize these chemicals as the sole carbon source for energy and growth. 1% stocks of the following chemicals were tested: cyclohexyl chloride, phenanthrene, napthalene, benzoic acid and phenyl acetate. 2% (v/v) stocks of fresh and used car motor oil 10W30 were also tested. Chemical doses used are given in mL in the file name. medium plates were imaged after two weeks of growth at room temperature (22C) [file f1000research-9-27904-s0003.tgz › CC45330.5mLbenzoicacid.jpg]

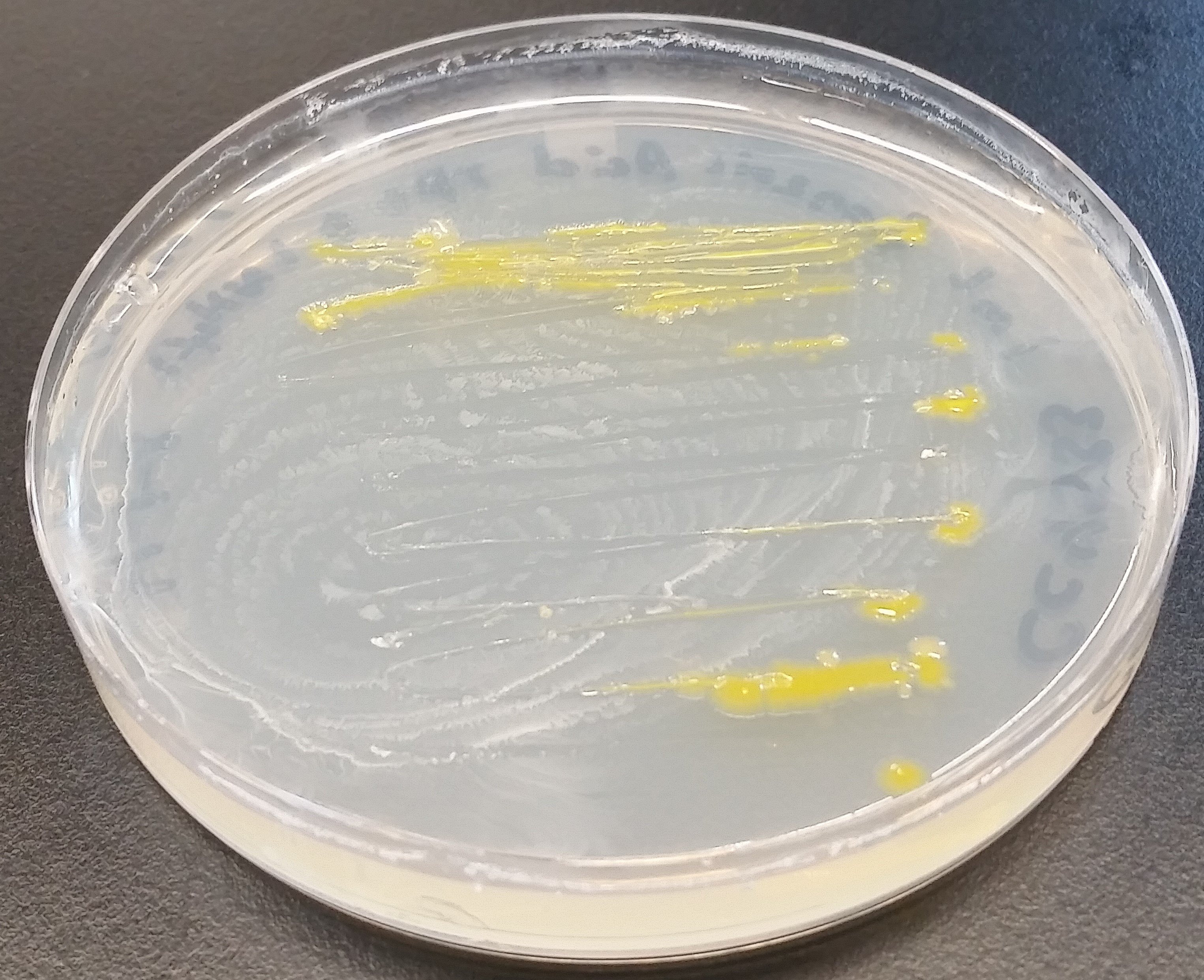

Supplement: Tests using Tris-Phosphate medium (TP) to see if hydrocarbons, aromatic compounds and polyhydroxyalkanoates can be used by the bacterium CC4533 (Sphingobium yanoikuyae PR86 strain variant, partial 16S rRNA sequence; GenBank Accession # MN633285.1) as the sole carbon source. — The file contains 21 images of TP (Tris-Phosphate) medium plates containing different alternative carbon sources. Bacterium CC4533 (Sphingobium yanoikuyae PR86 strain variant) was streaked on these chemical plates to test if CC4533 can utilize these chemicals as the sole carbon source for energy and growth. 1% stocks of the following chemicals were tested: cyclohexyl chloride, phenanthrene, napthalene, benzoic acid and phenyl acetate. 2% (v/v) stocks of fresh and used car motor oil 10W30 were also tested. Chemical doses used are given in mL in the file name. medium plates were imaged after two weeks of growth at room temperature (22C) [file f1000research-9-27904-s0003.tgz › CC45331mLbenzoicacid.jpg]

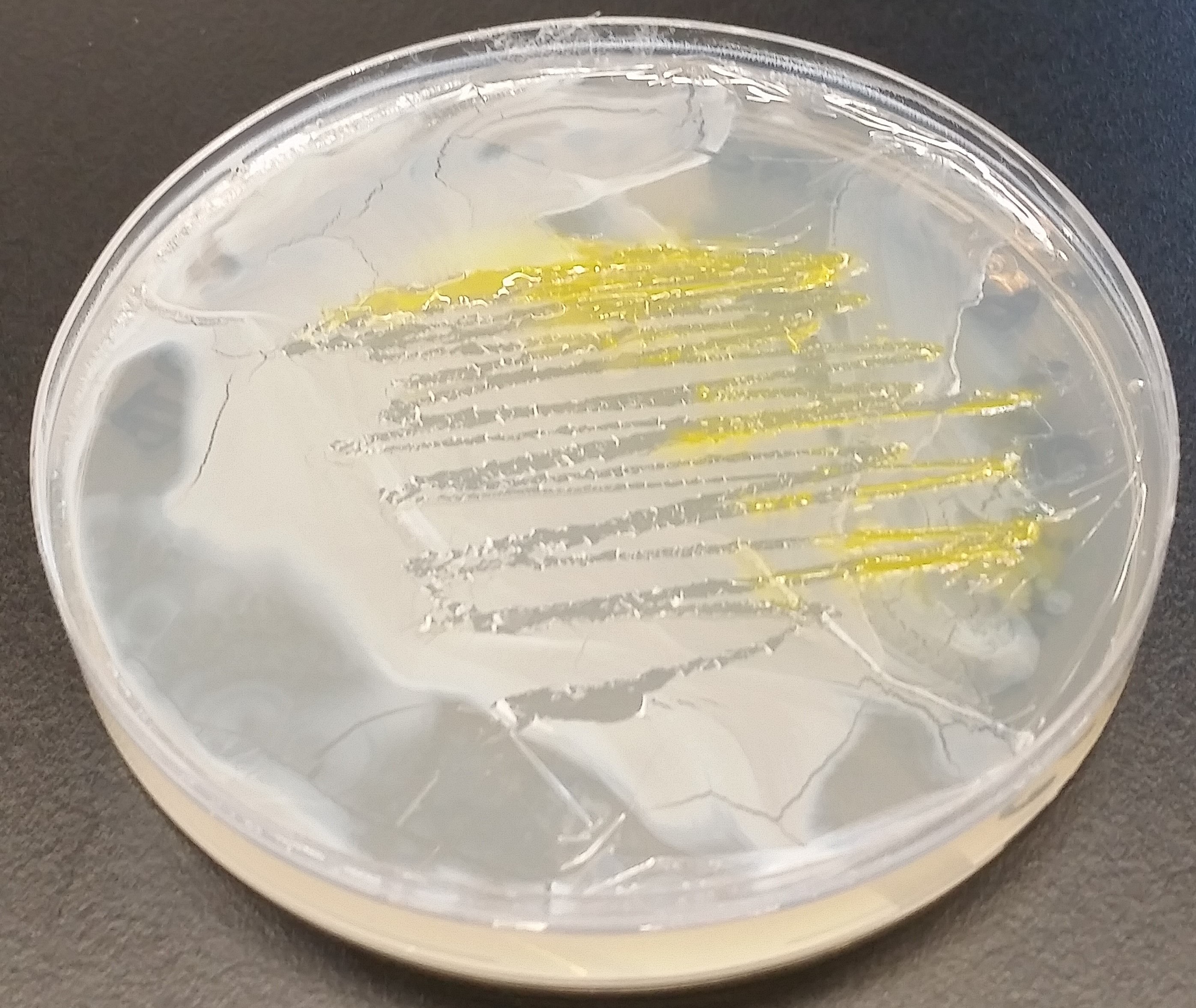

Supplement: Tests using Tris-Phosphate medium (TP) to see if hydrocarbons, aromatic compounds and polyhydroxyalkanoates can be used by the bacterium CC4533 (Sphingobium yanoikuyae PR86 strain variant, partial 16S rRNA sequence; GenBank Accession # MN633285.1) as the sole carbon source. — The file contains 21 images of TP (Tris-Phosphate) medium plates containing different alternative carbon sources. Bacterium CC4533 (Sphingobium yanoikuyae PR86 strain variant) was streaked on these chemical plates to test if CC4533 can utilize these chemicals as the sole carbon source for energy and growth. 1% stocks of the following chemicals were tested: cyclohexyl chloride, phenanthrene, napthalene, benzoic acid and phenyl acetate. 2% (v/v) stocks of fresh and used car motor oil 10W30 were also tested. Chemical doses used are given in mL in the file name. medium plates were imaged after two weeks of growth at room temperature (22C) [file f1000research-9-27904-s0003.tgz › CC45332mLbenzoicacid.jpg]

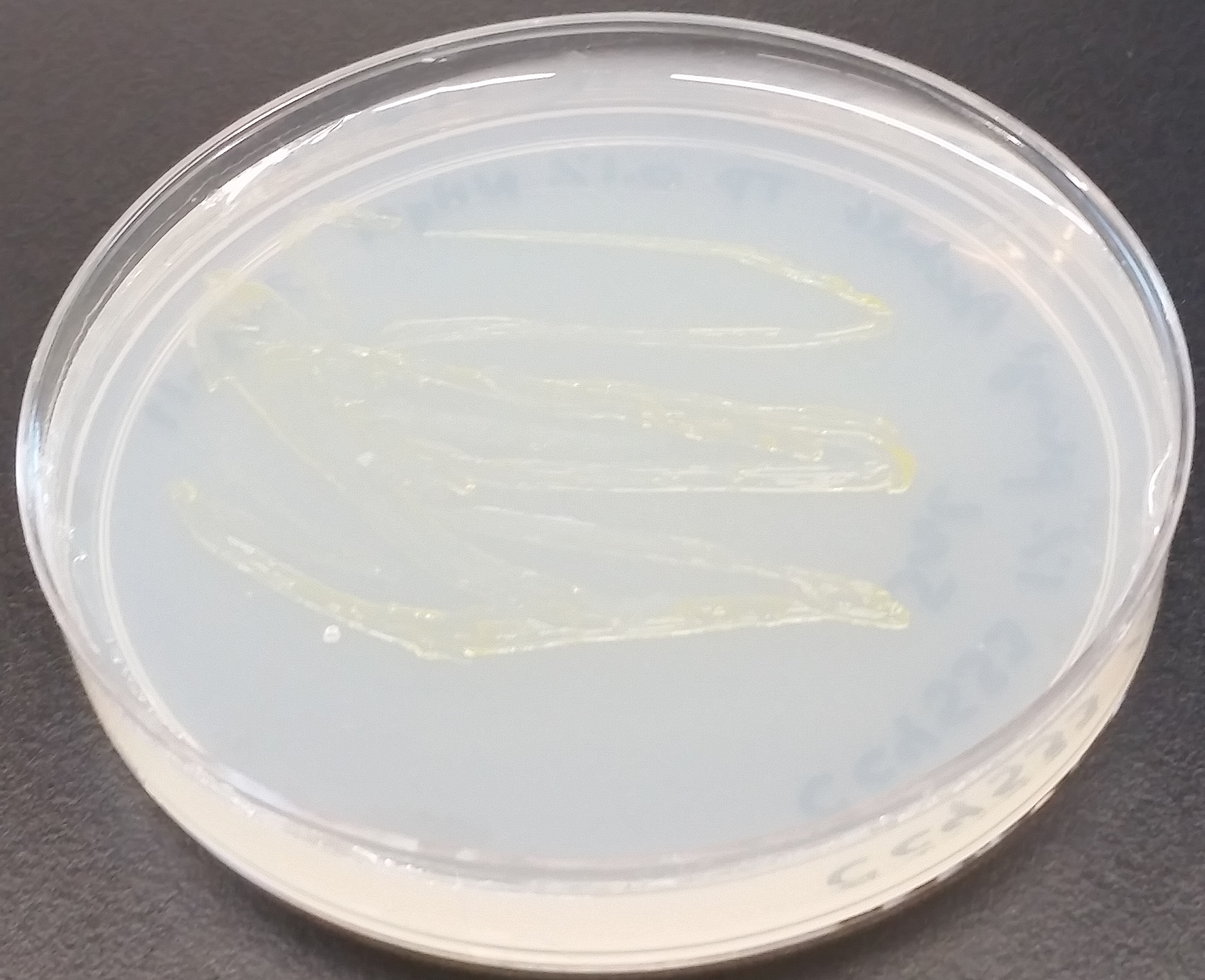

Supplement: Tests using Tris-Phosphate medium (TP) to see if hydrocarbons, aromatic compounds and polyhydroxyalkanoates can be used by the bacterium CC4533 (Sphingobium yanoikuyae PR86 strain variant, partial 16S rRNA sequence; GenBank Accession # MN633285.1) as the sole carbon source. — The file contains 21 images of TP (Tris-Phosphate) medium plates containing different alternative carbon sources. Bacterium CC4533 (Sphingobium yanoikuyae PR86 strain variant) was streaked on these chemical plates to test if CC4533 can utilize these chemicals as the sole carbon source for energy and growth. 1% stocks of the following chemicals were tested: cyclohexyl chloride, phenanthrene, napthalene, benzoic acid and phenyl acetate. 2% (v/v) stocks of fresh and used car motor oil 10W30 were also tested. Chemical doses used are given in mL in the file name. medium plates were imaged after two weeks of growth at room temperature (22C) [file f1000research-9-27904-s0003.tgz › CC45330.5mLphenylacetate.jpg]

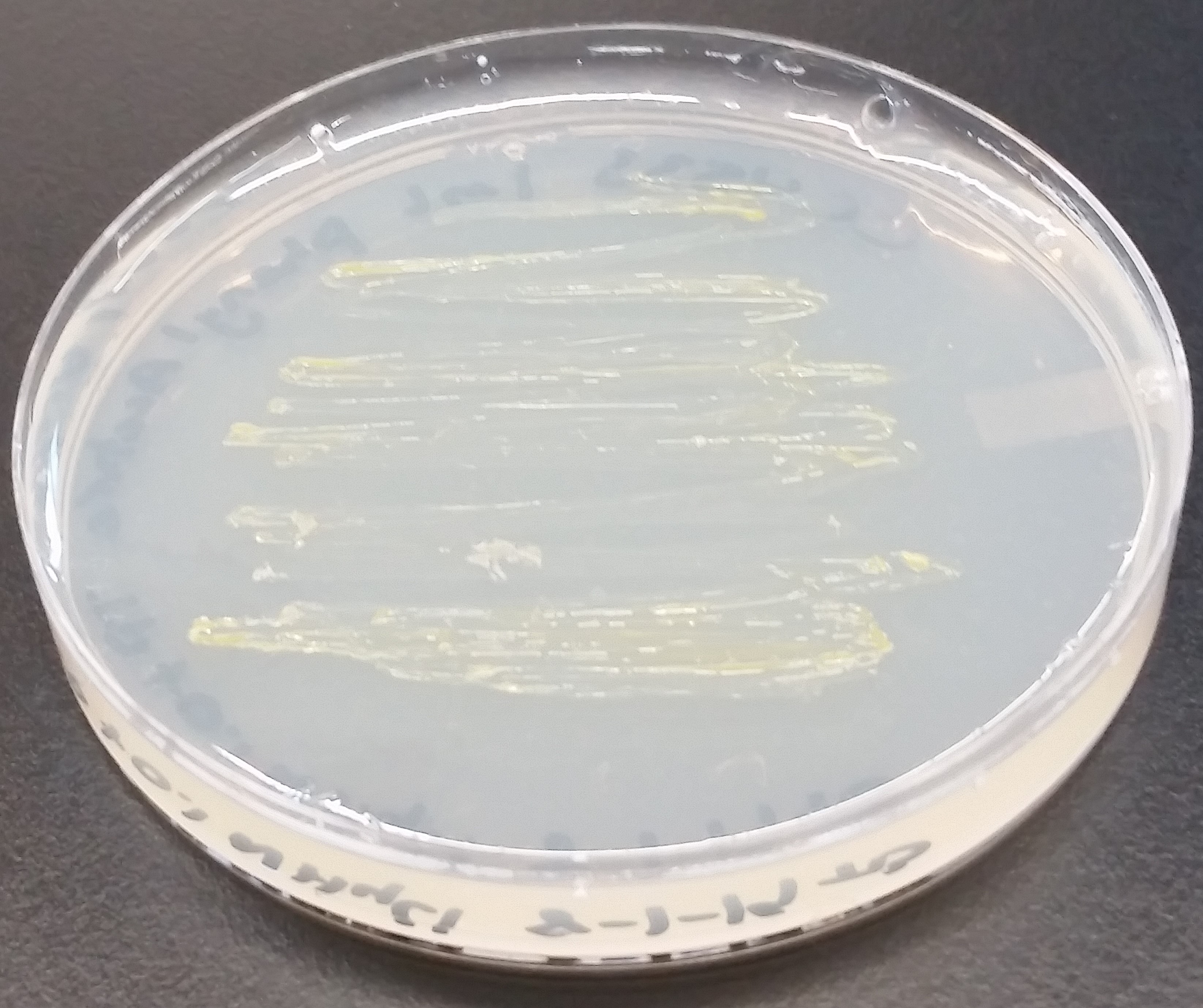

Supplement: Tests using Tris-Phosphate medium (TP) to see if hydrocarbons, aromatic compounds and polyhydroxyalkanoates can be used by the bacterium CC4533 (Sphingobium yanoikuyae PR86 strain variant, partial 16S rRNA sequence; GenBank Accession # MN633285.1) as the sole carbon source. — The file contains 21 images of TP (Tris-Phosphate) medium plates containing different alternative carbon sources. Bacterium CC4533 (Sphingobium yanoikuyae PR86 strain variant) was streaked on these chemical plates to test if CC4533 can utilize these chemicals as the sole carbon source for energy and growth. 1% stocks of the following chemicals were tested: cyclohexyl chloride, phenanthrene, napthalene, benzoic acid and phenyl acetate. 2% (v/v) stocks of fresh and used car motor oil 10W30 were also tested. Chemical doses used are given in mL in the file name. medium plates were imaged after two weeks of growth at room temperature (22C) [file f1000research-9-27904-s0003.tgz › CC45331mLphenylacetate.jpg]

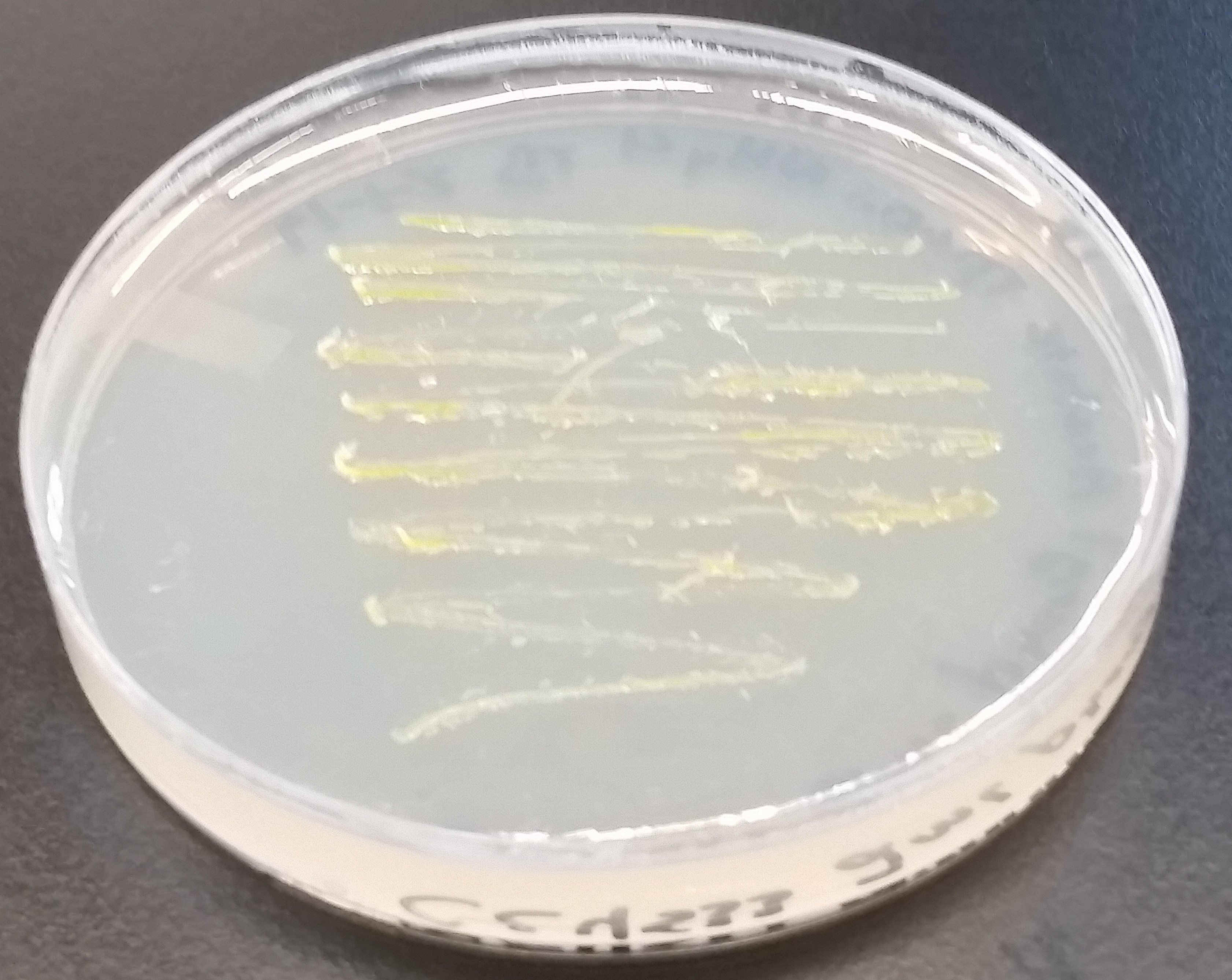

Supplement: Tests using Tris-Phosphate medium (TP) to see if hydrocarbons, aromatic compounds and polyhydroxyalkanoates can be used by the bacterium CC4533 (Sphingobium yanoikuyae PR86 strain variant, partial 16S rRNA sequence; GenBank Accession # MN633285.1) as the sole carbon source. — The file contains 21 images of TP (Tris-Phosphate) medium plates containing different alternative carbon sources. Bacterium CC4533 (Sphingobium yanoikuyae PR86 strain variant) was streaked on these chemical plates to test if CC4533 can utilize these chemicals as the sole carbon source for energy and growth. 1% stocks of the following chemicals were tested: cyclohexyl chloride, phenanthrene, napthalene, benzoic acid and phenyl acetate. 2% (v/v) stocks of fresh and used car motor oil 10W30 were also tested. Chemical doses used are given in mL in the file name. medium plates were imaged after two weeks of growth at room temperature (22C) [file f1000research-9-27904-s0003.tgz › CC45332mLphenylacetate.jpg]
