# Supplementary material for: Isolation and characterization of a novel Sphingobium yanoikuyae strain variant that uses biohazardous saturated hydrocarbons and aromatic compounds as sole carbon sources
Source: F1000Res. 2020 Jul 24;9:767. [Version 1] doi: 10.12688/f1000research.25284.1 (PMC7477647; doi:10.12688/f1000research.25284.1)
Supplement: Peer review history

# Peer review: Reviewer response for version 1

**DOI:** 10.5256/f1000research.27904.r68970

**Published:** 2020-09-07

**Authors:** Arun Karnwal

**Affiliations:** 1 Department of Microbiology, School of Bioengineering and Biosciences, Lovely Professional University, Phagwara, Punjab, India

The manuscript is well written, has important data related to be used in future in bioremediation of hazardous saturated hydrocarbons using *Sphingobium yanoikuyae* and should be of great interest to the readers.

 Introduction: it is written very well and comprises the basic information regarding bacterial species, method applied during research.

 Methods: methodology used for characterisation of bacterial strain is appropriate i.e. microscopic, macroscopic and molecular.

 Results and Discussion are presented properly and discussed with appropriate earlier work.

 Overall, it is an important study, and should be considered for indexing.

Is the work clearly and accurately presented and does it cite the current literature?

Yes

If applicable, is the statistical analysis and its interpretation appropriate?

Yes

Are all the source data underlying the results available to ensure full reproducibility?

Yes

Is the study design appropriate and is the work technically sound?

Yes

Are the conclusions drawn adequately supported by the results?

Yes

Are sufficient details of methods and analysis provided to allow replication by others?

Yes

Reviewer Expertise:

Environment Microbiology, Agricultural Microbiology

I confirm that I have read this submission and believe that I have an appropriate level of expertise to confirm that it is of an acceptable scientific standard.

# Peer review: Reviewer response for version 1

**DOI:** 10.5256/f1000research.27904.r68971

**Published:** 2020-09-03

**Authors:** David Dewez

**Affiliations:** 1 Laboratory of Environmental and Analytical Biochemistry of Contaminants, Department of Chemistry, University of Quebec at Montreal, Montreal, QC, Canada

This research was well performed, and the paper explains in detail the obtained results and the outcome.

 This work provides original results and contributes significantly to the domain of environmental microbiology. The paper is a little bit long, but the quality is not affected. Overall, the structure of this article is good, and this research work is well supported by an extensive bibliography.

 However, I do not recommend using the term “dose”, since in toxicology domain it is use for the quantity absorbed by an organism per his weight (for example in µg/Kg). It may bring confusion for readers. In addition, the label of Figure 2-D should be corrected for 100 µg/mL instead of 50 µg/mL, and the label of Figure 17-B should be corrected for 2 mL instead of 0.5 mL.  

 Finally, I approve the publication of this research article.

Is the work clearly and accurately presented and does it cite the current literature?

Yes

If applicable, is the statistical analysis and its interpretation appropriate?

Yes

Are all the source data underlying the results available to ensure full reproducibility?

Yes

Is the study design appropriate and is the work technically sound?

Yes

Are the conclusions drawn adequately supported by the results?

Yes

Are sufficient details of methods and analysis provided to allow replication by others?

Yes

Reviewer Expertise:

Environmental toxicology and chemistry

I confirm that I have read this submission and believe that I have an appropriate level of expertise to confirm that it is of an acceptable scientific standard.
